# Pharmacological and procedural strategies to prevent post-ERCP pancreatitis: an umbrella review of meta-analyses of randomized controlled trials

**DOI:** 10.1186/s12916-026-05115-0

**Published:** 2026-08-06

**Authors:** Mengruo Jiang, Liqi Sun, Lisi Peng, Deyu Zhang, Chao Liu, Lixiang Wang, Yuwei Sun, Zhendong Jin, Zhaoshen Li, Saif Ullah, Haojie Huang

**Affiliations:** 1 Department of Gastroenterology, National Clinical Research Center for Digestive Diseases, Changhai Hospital, Navy Medical University, Shanghai, China; 2 Department of Gastroenterology, 72nd Group Army Hospital, Huzhou University, Huzhou, Zhejiang China; 3 National Key Laboratory of Immunity and Inflammation, Naval Medical University, Shanghai, China

**Keywords:** Endoscopic retrograde cholangiopancreatography, Post-endoscopic retrograde cholangiopancreatography pancreatitis, Pharmacological interventions, Procedural technical modifications

## Abstract

**Background:**

Post-endoscopic retrograde cholangiopancreatography (ERCP) pancreatitis (PEP) is the most common complication following ERCP, and its prevention remains a major concern for biliopancreatic endoscopists. This umbrella review aims to assess existing meta-analytic evidence on the effectiveness of pharmacological interventions (PIs) and procedural technical modifications (PTMs) in reducing the incidence and severity of PEP.

**Methods:**

We searched multiple databases to identify meta-analyses of randomized controlled trials (RCTs) that assessed the impacts of PIs or PTMs on the incidence and severity of PEP, from database inception to September 24, 2024. Evidence credibility was classified based on pre-specified criteria as: convincing (Class I), highly suggestive (Class II), suggestive (Class III), weak (Class IV), or non-significant evidence. Furthermore, the GRADE approach was employed to assess the quality of the evidence, categorizing it into four levels: high, moderate, low, and very low.

**Results:**

A review of 47 meta-analyses (137,391 participants; 25 on PIs, 22 on PTMs) identified five PIs with suggestive evidence for PEP prevention: indomethacin (odds ratio [OR] 0.52, 95% confidence interval [CI] 0.39–0.71), epinephrine (risk ratio [RR] 0.34, 95% CI 0.18–0.62), nafamostat mesylate (RR 0.50, 0.38–0.66), NSAIDs (RR 0.65, 0.52–0.81), and aggressive hydration (OR 0.40, 0.26–0.63). For PTMs, one intervention was classified as highly suggestive evidence: pancreatic stent placement (RR 0.37, 0.27–0.51), while guidewire-assisted cannulation was suggestive (RR 0.51, 0.36–0.72). For high-risk patients, both indomethacin (RR 0.46, 0.32–0.65) and pancreatic stent placement (RR 0.41, 0.30–0.56) were suggestive. No interventions exceeded Class III evidence for preventing moderate to severe PEP.

**Conclusions:**

Pancreatic stent placement and NSAIDs, such as indomethacin, have been confirmed as effective measures for PEP prevention in both unselected and high-risk patients.

**Graphical abstract:**

**Figure:**
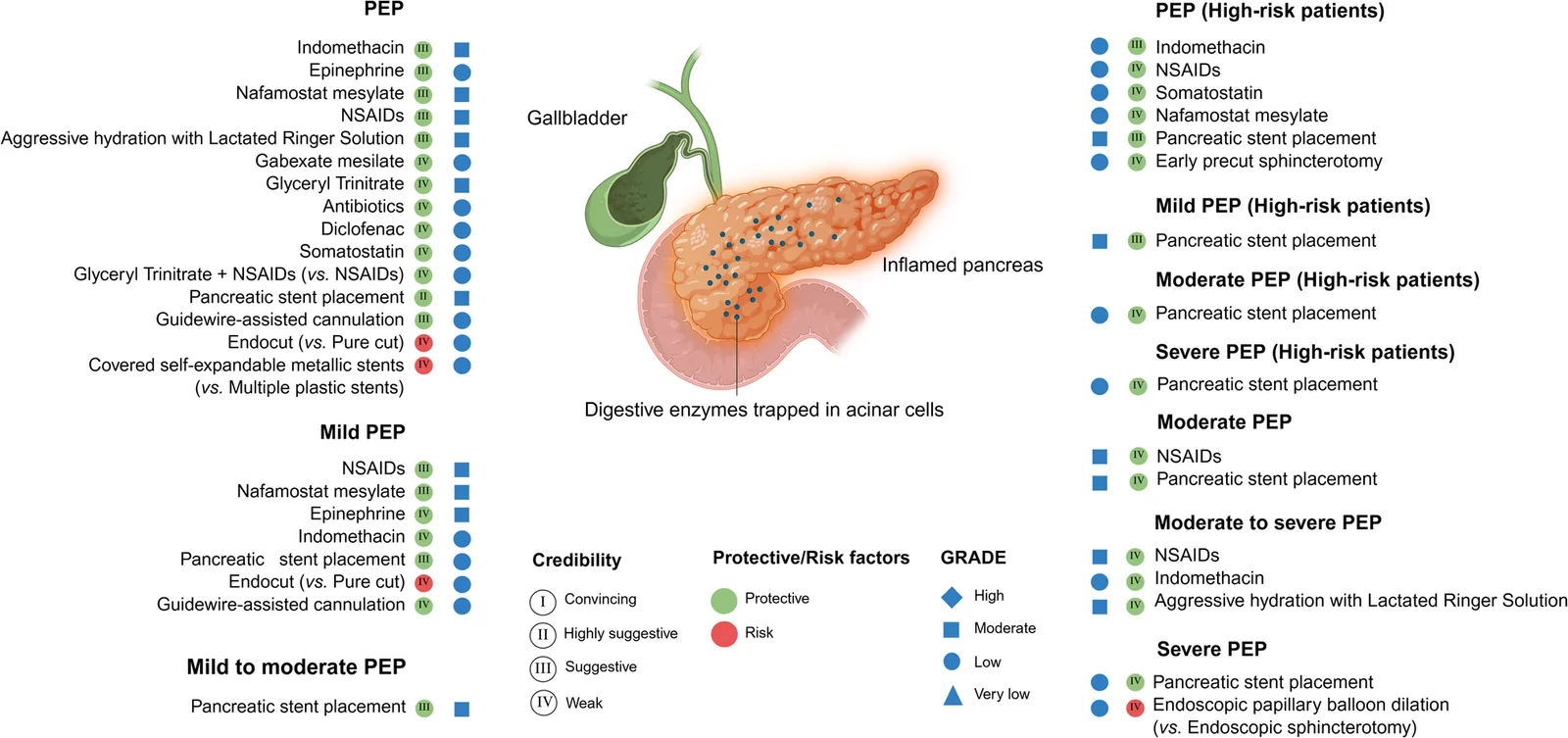

**Supplementary Information:**

The online version contains supplementary material available at https://doi.org/10.1186/s12916-026-05115-0.

## Background

Since its introduction in 1968 [1], endoscopic retrograde cholangiopancreatography (ERCP) has become an essential therapeutic and diagnostic modality for managing a wide range of pancreaticobiliary disorders [2–4]. Despite its clinical utility, ERCP is associated with a relatively high risk of adverse events (AEs), the most common of which is post-endoscopic retrograde cholangiopancreatography pancreatitis (PEP), occurring in approximately 7% of cases [5–7]. While the majority of PEP cases are mild, a subset progresses to moderate or severe pancreatitis, necessitating intensive medical management or invasive interventions, with a reported mortality rate ranging from 0.3% to 0.6% [8]. As such, the prevention of PEP remains a major clinical concern and research priority for biliopancreatic endoscopists.

Considerable research has been directed at identifying both patient-related and procedural risk factors for PEP. Patient-related risk factors, such as suspected sphincter of Oddi dysfunction, female sex, and a history of pancreatitis or previous PEP, are largely non-modifiable and thus present limited opportunities for intervention [9, 10]. In contrast, pharmacological interventions (PIs) and procedural technical modifications (PTMs) represent modifiable factors that can be leveraged to reduce the risk and severity of PEP. These strategies can be broadly categorized into PIs, such as non-steroidal anti-inflammatory drugs (NSAIDs) [11, 12], and PTMs, including pancreatic duct stenting [13] and guidewire-assisted cannulation [14].

Over the past decade, numerous randomized controlled trials (RCTs) have been conducted to evaluate the efficacy of various PIs and PTMs in PEP prevention, and their findings have been synthesized through multiple meta-analyses and guidelines. While guidelines (ESGE 2020 [6], ASGE 2023 [15]) provide essential, consensus-driven recommendations for PEP prevention, they are inherently selective and often focus on a limited set of interventions deemed to have the strongest supporting evidence at the time of publication. Similarly, conventional pairwise meta-analyses typically evaluate a single intervention or a narrow comparison. Consequently, there remains a lack of a comprehensive, systematic, and hierarchical synthesis that concurrently evaluates and grades the strength of evidence for previously studied PIs and PTMs across different patient populations (e.g., unselected vs. high-risk) and PEP severity outcomes. Therefore, we conducted an umbrella review (UR) of meta-analyses of RCTs to assess the effectiveness of PIs and PTMs in preventing PEP. Our aim was to critically evaluate and classify the strength of the current evidence base, thereby guiding clinical practice and informing future research directions in the prevention of this significant ERCP-related complication.

## Methods

### Reporting guidelines and protocol registration

This study was reported following the AMSTAR (Assessing the Methodological Quality of Systematic Reviews) [16] and PRISMA (Preferred Reporting Items for Systematic Reviews and Meta-Analyses) guidelines [17]. This UR was prospectively registered with PROSPERO (CRD42024627420), and the registration is available online.

### Literature search

Two researchers (J.M.R. and S.L.Q.) independently searched PubMed, Web of Science, Embase, and the Cochrane Library from inception to September 24, 2024, for meta-analyses examining PIs or PTMs that affect the incidence or severity of PEP. The detailed search strategy for each database is presented in Additional file 1: Table S1. Searches were supplemented by a manual search of reference lists from eligible studies.

### Eligibility criteria

The selection of potentially relevant meta-analyses was made by inspection of titles and abstracts by two reviewers independently (J.M.R. and S.L.Q.). When the titles and abstracts did not provide sufficient information, the full articles were obtained for further eligibility verification. In cases of discrepancies, a third reviewer (P.L.S.) was consulted.

#### Inclusion criteria

Meta-analyses of RCTs, specifically those examining the effects of PIs or PTMs on the incidence of PEP and PEP severity. The severity of PEP was defined according to Cotton’s criteria [18]. Mild PEP was defined as clinical pancreatitis with amylase levels at least three times the normal level more than 24 h post-procedure, necessitating admission or extension of a planned admission to 2–3 days. Moderate PEP required hospitalization for 4–10 days. Severe PEP was characterized by hospitalization exceeding 10 days, complications including hemorrhagic pancreatitis, phlegmon, or pseudocyst formation, or the need for intervention (percutaneous drainage or surgery). Combined severity categories (e.g., mild to moderate, moderate to severe) were derived from these core definitions. A high-risk patient is defined by the presence of at least one definitive patient-related risk factor, such as suspected sphincter of Oddi dysfunction, female sex, age under 60 years, normal serum bilirubin, or a history of pancreatitis/previous PEP. Meanwhile, a high-risk procedure/setting is defined by operational factors encountered during the intervention, which include difficult cannulation (e.g., > 5 insertion attempts or a cannulation time > 5 min), inadvertent and repeated guidewire entry into the pancreatic duct, contrast medium injection into the pancreatic duct, precut sphincterotomy, pancreatic sphincterotomy, or endoscopic papillary balloon dilation in patients with an intact sphincter [6, 19–21]. Under this framework, patients are classified into the high-risk cohort if they present with at least one major patient- or procedure-related factor.

#### Exclusion criteria

(1) Meta-analyses that did not provide sufficient data for re-analysis. (2) Network meta-analyses (NMAs). Our methodology was designed to summarize and grade the credibility of evidence derived from pairwise meta-analyses of direct comparisons. The quantitative analysis method of the umbrella review is not applicable to NMAs. (3) Studies not written in English.

If a study presented meta-analyses on more than one eligible outcome (e.g., incidence of PEP, PEP in high-risk patients, or PEP of different severity), the different outcomes were assessed separately. Whenever more than one meta-analysis existed on the same scientific question, the meta-analysis with the largest number of studies was selected, with all other redundant meta-analyses excluded to ensure mutual exclusivity.

#### Overlapping meta-analyses

To address overlap when multiple meta-analyses existed on the same scientific association, we constructed a citation matrix to map the primary RCTs included in each meta-analysis. We quantified the overlap using the Corrected Covered Area (CCA) method [22]. The degree of overlap was categorized as slight (0–5%), moderate (6–10%), high (11–15%), and very high (> 15%).

### Data extraction

A standardized data extraction form was utilized to collect relevant data. From each eligible meta-analysis, two investigators (J.M.R. and S.L.Q.) abstracted information independently on the first author, year of publication, database, date of searches, number of included studies and participants, risk of bias assessment tool for RCTs, outcome of interest, types of interventions, comparators, and metrics used in the original analyses. Additionally, we extracted individual study-level data for each study within the meta-analysis, such as the number of cases and participants in the intervention or control groups, as well as relative effect measures like odds ratio (OR) or relative risk (RR). For the primary outcome (incidence of PEP), we conducted subgroup analyses stratified by the intervention’s timing, route, dose, regimen, and operator expertise. Any inconsistencies in the data extraction were resolved through discussion with a third reviewer (P.L.S.).

### Quality assessment of methods and evidence

The quality of the included meta-analyses was independently assessed by two reviewers (J.M.R. and S.L.Q.) using AMSTAR-2 (A Measurement Tool to Assess Systematic Reviews) [16]. Based on these criteria, the qualities of the meta-analyses were rated as high, moderate, low, or critically low. In cases of disagreement, a third reviewer (P.L.S.) was consulted.

The quality of evidence was evaluated using the Grading of Recommendations, Assessment, Development, and Evaluation (GRADE) framework [23, 24], which categorizes evidence into four levels: high, moderate, low, and very low. Initially, we rated the quality of evidence from RCTs as high. However, the quality of evidence rating could be reduced by factors such as risk of bias, inconsistency, indirectness, a lack of accuracy, or publication bias.

### Data analysis

For each intervention-outcome pair, we recalculated the summary effect size, 95% confidence interval (CI), and p-value using inverse variance random effects methods, with the statistical threshold set at p-value < 0.05, 10⁻³, and 10⁻⁶. Additionally, we checked whether the summary effect of the random effects meta-analysis and the effect of its largest component study (the study with the smallest standard error) were concordant in terms of statistical significance. Effect size measures for the association between each PI or PTM and PEP incidence included OR, RR, and risk difference (RD). In our study, the OR and RR were applicable for further analyses. Therefore, studies reporting RD or systematic reviews without pooled results were recalculated to obtain the RR using data from the original RCTs. Furthermore, to address the potential inconsistency in the analysis results caused by the mixed use of OR and RR, we have supplemented a sensitivity analysis using RR as the single effect size. For meta-analyses that included both RCTs and non-RCTs, we removed the non-RCTs and reanalyzed the data from RCTs with inverse variance random effects methods to assume the existence of real differences across studies. RCTs in which both the intervention and control groups had zero cases were also excluded from the summary effect size analysis.

The heterogeneity between studies was assessed using the I² metric with a 95% CI. I² > 50% was considered indicative of high heterogeneity. Additionally, we calculated 95% prediction intervals for the summary random-effects estimates. The 95% prediction intervals further account for heterogeneity between studies and indicate where the true effects are for 95% of the studies from the population of studies that are synthesized or similar studies that might be conducted in the future. Prediction intervals excluding the null value suggest that the association is likely to persist in future studies.

To verify whether small studies tend to yield higher effect sizes than large studies, we performed Egger’s regression asymmetry test, and a p-value of 0.10 or less was considered evidence of small-study effects bias. We assessed excess significance bias by evaluating whether the observed number of studies with nominally statistically significant results (positive studies, p-value < 0.05) in each meta-analysis was larger than the expected number. We used a χ² test, with a p-value ≤ 0.10 indicating the presence of excess significance bias. Statistical analyses were conducted using Stata version 16 and R version 4.4.2. The complete analysis code supporting the findings of this study has been deposited in the Open Science Framework (OSF) repository and is available at https://osf.io/8jdrb/overview?view_only=a1243851359944b7888a876ad3db9d36.

### Determining the credibility of evidence

We applied a previously proposed UR methodology to assess the strength of evidence [25]. Based on data from our re-analyses—including p-values from random-effects models, sample sizes, statistical significance of the largest study, heterogeneity (I²), small-study effects, excess significance bias, and the 95% prediction interval—we categorized each re-meta-analyzed result into one of the following classes: convincing (Class I), highly suggestive (Class II), suggestive (Class III), weak (Class IV), or non-significant evidence. The evidence classifications based on the criteria are outlined in Table 1.

**Table 1:** Classification of evidence in meta-analyses for standardized effect sizes

**Suggestive evidence (Class III)** • >1000 cases • *p*<10^−^³ for random effect models • Class II was not met
**Highly suggestive evidence (Class II)** • >1000 cases • *p*<10^−^⁶ for random effect models • Largest study with a statistically significant effect • Class I was not met
**Convincing evidence (Class I)** • >1000 cases • *p*<10^−^⁶ for random effect models • Low to moderate between-studies heterogeneity (I²<50%) • 95% prediction interval excluding the null • Largest study with a statistically significant effect • No small-study effects • No excess significance bias
**Weak evidence (Class IV)** • *p*<0.05 • Class III was not met
**Non-significant evidence** • *p*>0.05

## Results

### Literature search

**Fig. 1:**
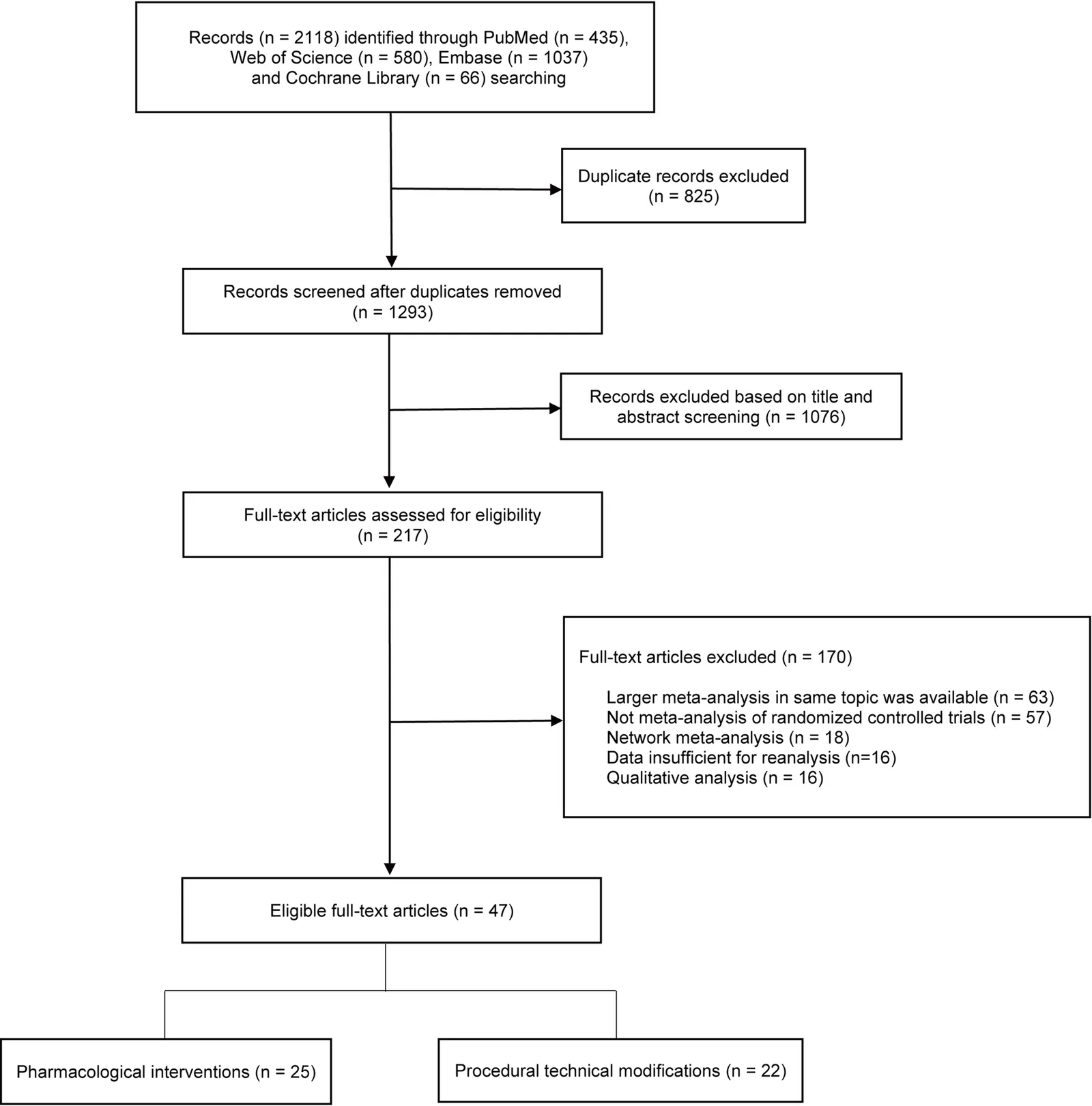
Preferred Reporting Items for Systematic Reviews and Meta-Analyses (PRISMA) flowchart

From the database inception to September 24, 2024, 47 meta-analyses met the inclusion criteria for this UR, comprising 25 studies examining the effects of PIs [11, 19, 20, 26–47] and 22 investigating the effects of PTMs [14, 21, 48–67] on the incidence and severity of PEP. The study selection flowchart is presented in Fig. 1. Details of the excluded records and reasons for exclusion are provided in Additional file 1: Table S2.

### Characteristics of included studies

This UR included 47 meta-analyses, encompassing 540 RCTs with a total of 137,391 participants. The included studies were published between 2007 and 2024. Methodological considerations revealed that most RCTs assessed the risk of bias in RCTs using tools like risk of bias (ROB), ROB 2, and the Jadad score.

PIs examined included NSAIDs (not drug-specific), indomethacin, diclofenac, topical administration of epinephrine, nafamostat mesylate, somatostatin, gabexate mesylate, glyceryl trinitrate, antioxidants, antibiotics, allopurinol, ulinastatin, octreotide, corticosteroids, aggressive hydration with Lactated Ringer Solution, and various drug combinations. For PTMs, interventions such as pancreatic stent placement, guidewire-assisted cannulation, the double-guidewire technique, endoscopic sphincterotomy, endoscopic papillary large balloon dilation, endoscopic papillary balloon dilation (EPBD), early precut sphincterotomy, needle knife fistulotomy, needle-knife precut, and modes of electric current (e.g., endocut, pure cut, blend) were included. In terms of outcomes, except for overall PEP incidence, the PEP severities, including mild, moderate, severe, and combined categories (mild to moderate, moderate to severe), were further analyzed. Special attention was given to high-risk populations. The median number of involved RCT studies and participants was 10 (range from 3 to 26) and 2,632, respectively. A detailed summary of each meta-analysis is outlined in Table 2.

**Table 2:** Characteristics of 47 systematic reviews and meta-analyses on the prevention of PEP through pharmacological interventions or procedural technical modifications

Source	Outcome	Intervention	Comparator	No. of primary studies	No. of participants in meta-analysis	Metrics	Search databases and time	Quality assessment tool for RCTs	AMSTAR-2 rating
**Pharmacological interventions**									
Cao, W. L. et al, 2014	PEP, Mild PEP,Moderate PEP, Severe PEP	Allopurinol	Placebo	6	1974	RR	PubMed, Web of Science, Embase, Cochrane Library (May 2014)	RoB	Low
Chen, Z. et al, 2024	Mild PEP,Moderate to severe PEP	Epinephrine orEpinephrine + NSAIDs	Placebo/No treatment orNSAIDs	10	5683	RR	PubMed, Web of Science, Embase, Cochrane Library (November 2022)	RoB	Critically low
Cheng, X. et al, 2022	PEP, Severe PEP	Aggressive hydration + rectal NSAIDs	rectal NSAIDs	3	1110	OR	PubMed, Web of Science, Embase, Cochrane Library, ClinicalTrials.gov, CNKI, CQVIP, Wanfang Data (September 2021)	RoB	Critically low
Chiu, Y. J. et al, 2021	PEP	Gabexate mesilate	Placebo/No treatment	13	3718	RR	PubMed, Embase, Cochrane Library (NR)	RoB	Low
Ding, J. et al, 2013	PEP,Moderate to severe PEP	Glyceryl Trinitrate	Placebo/Physiological saline/Vitamin C/No intervention	12	2649	RR	PubMed (May 2013), Embase (May 2013), Cochrane Library (Apr 2013), ISI Web of Knowledge (May 2013)	Jadad	Low
El Hage Chehade, N. et al, 2023	PEP	Epinephrine + NSAIDs	NSAIDs	6	4016	OR	Cochrane Library, Medline/PubMed, Scopus (May 2021)	RoB	Low
El Kurdi, B. et al, 2024	PEP,Moderate to severe PEP	NSAIDs or Indomethacin or Diclofenac	No NSAIDs	25	7688	OR	PubMed, Embase, Cochrane Library (January 2000-February 2020)	RoB and Jadad	Low
Fuentes-Orozco, C. et al, 2015	PEP, Mild PEP,Moderate PEP, Severe PEP	Antioxidants	Placebo/No intervention	12	3110	RR	NR	NR	Critically low
Hou, Y. C. et al, 2017	Mild PEP	NSAIDs	Placebo/Glycerin/No treatment	16	6458	RR	PubMed, Embase, Cochrane Library (August 2016)	RoB	Critically low
Iqbal, U. et al, 2020	PEP	Epinephrine	Placebo/No treatment	8	4123	RR	PubMed, Embase, Cochrane Library (August 2019)	Jadad	Moderate
Lyu, Y. et al, 2018	PEP (High-risk patients)	NSAIDs or Diclofenac	Placebo	21	6134	RR	PubMed, Web of Science, Embase, Cochrane Library, ClinicalTrials.gov (August 1990-August 2017)	RoB and Jadad	Low
Merchan, M. F. S. et al, 2022	PEP	Antibiotics	Non-antibiotics	10	1757	RD	Embase, Cochrane Library, Medline, ClinicalTrials.gov, LILACS (February 2022)	RoB 2	Low
Narumi, K. et al, 2023	PEP, Mild PEP,Moderate to severe PEP	Nafamostat mesylate	Placebo/No treatment	9	3321	RR	PubMed, Web of Science, CENTRAL, Ichushi Web (October 2023)	RoB 2	Low
Serrano, J. P. R. et al, 2020	PEP,Moderate PEP, Severe PEP	NSAIDs or Diclofenac	Placebo SN/Placebo lactated Ringer’s solution/Ceftazidime/Glycerin/Glyceryl trinitrate/Saline solution /No treatment	26	8143	RD	Embase, Cochrane Library, Medline/PubMed (December 2019)	Jadad	Moderate
Wan, J. et al, 2017	Mild PEP,PEP (High-risk patients)	Indomethacin	Placebo	7	3013	RR	PubMed, Embase, Cochrane Library (June 2016)	RoB	Low
Wang, G. et al, 2018	PEP,PEP (High-risk patients)	Somatostatin	Placebo/No treatment	15	4643	OR	Web of Science, Embase, Medline/PubMed,CENTRAL (NR)	RoB	Low
Wang, R. C. et al, 2021	Moderate PEP	Aggressive hydration with Lactated Ringer Solution	Standard hydration with Lactated Ringer Solution	7	1278	OR	PubMed, Web of Science, Embase, Cochrane Library (January 2009-January 2019)	RoB and Jadad	Low
Wu, D. et al, 2017	Moderate to severe PEP, PEP (High-risk patients)	Aggressive hydration with Lactated Ringer Solution	Standard hydration with Lactated Ringer Solution	3	722	OR	PubMed, Web of Science, Embase, Cochrane Library, Scopus, ClinicalTrials.gov (July 2016)	RoB	Low
Wu, M. et al, 2021	PEP	Aggressive hydrationwith Lactated Ringer Solution	Standard hydrationwith Lactated Ringer Solution	10	2200	OR	PubMed, Web of Science, Embase, Cochrane Library, Scopus, ClinicalTrials.gov, CNKI, CQVIP, WanFang Data (April 2019)	RoB	Low
Xie, Y. et al, 2023	Moderate PEP, Severe PEP,PEP (High-risk patients)	Nafamostat mesylate	Placebo/No treatment	8	3210	RR	PubMed, Web of Science, Embase, CENTRAL, Scopus (August 2022)	RoB and Jadad	Moderate
Yuhara, H. et al, 2014	PEP	Ulinastatin	Placebo	26	9908	RR	PubMed (September 2012)	Jadad	Critically low
Zhang, X. et al, 2024	PEP, Non-mild PEP	Glyceryl Trinitrate + NSAIDs	NSAIDs	10	3240	RR	PubMed, Web of Science, Embase, Cochrane Library (September 2022)	Jadad	Low
Zhang, Y. et al, 2009	PEP	Octreotide	Placebo	18	3983	OR	Embase, Cochrane Library, Medline, CENTRAL, Science Citation Index (March 2008)	Jadad	Low
Zheng, M. et al, 2008	PEP, Severe PEP	Corticosteroid	Placebo	7	2632	OR	Embase (January 1966-June 2007), Cochrane Library (Issue 2, 2007), Medline (January 1966-June 2007), CBMdisc (January 1978-June 2007)	Jadad	Low
Zheng, M. H. et al, 2008	Severe PEP	Gabexate mesilate	Placebo/No treatment	7	2883	OR	Embase (January 1966-July 2007), Cochrane Library (Issue 2, 2007), Medline (January 1966-July 2007), CBMdisc (January 1978-July 2007)	Jadad	Low
**Procedural technical modifications**									
Aziz, Muhammad et al, 2022	PEP	0.035-inch guidewire	0.025-inch guidewire	3	1079	RR	Embase, Web of Science, Medline/PubMed, CENTRAL, Global Index Medicus (November 2021)	RoB	Low
Bicudo de Oliveira, L. et al, 2024	PEP, Mild PEP,Moderate PEP, Severe PEP	Endocut	Pure cut	4	987	RD	Embase, Cochrane Library, Medline, Google Scholar, LILACS (June 2023)	RoB 2	Critically low
Choudhary, A. et al, 2014	PEP	Early precut sphincterotomy	Persistent cannulation attempts	14	4580	OR	Embase, Medline, CENTRAL (1966-June 2013)	Jadad	Low
da Cruz Portela, Joaquim Coelho et al, 2019	Mild PEP (High-risk patients), Moderate PEP (High-risk patients), Severe PEP (High-risk patients)	Pancreatic stent placement	No Pancreatic stent placement	12	3310	RD	Embase, Medline/PubMed, CENTRAL, LILACS (October 2016)	Jadad	Low
de Clemente Junior, C. C. et al, 2018	PEP	Endoscopic sphincterotomy associated with balloon dilation	Endoscopic sphincterotomy	11	1824	RD	Embase, Cochrane Library, Medline/PubMed, LILACS, CAPES database (July 2017)	Jadad	Low
Fan, J. H. et al, 2015	Mild PEP, Severe PEP	Pancreatic stent placement	No Pancreatic stent placement	15	1606	OR	Embase, Cochrane Library, Medline (May 2013)	Modified tool based on RoB	Low
Funari, M. P. et al, 2020	Mild PEP, Moderate PEP	Endocut	Blend	11	1791	RD	Embase, Medline, Scopus, CINAHL, CENTRAL, LILACS (September 2019)	RoB	Moderate
Li, D. F. et al, 2019	PEP	Endocut	Blend	3	594	RR	PubMed, Cochrane Library (July 2018)	RoB and Jadad	Critically low
Liu, P. et al, 2019	PEP	Endoscopic papillary large balloon dilation	Endoscopic papillary large balloon dilation + Endoscopic sphincterotomy	7	976	OR	Embase, Cochrane Library, Medline (July 2018)	RoB	Critically low
Lyu, Y. et al, 2023	PEP (Patients with difficult cannulation)	Needle-knife precut	Transpancreatic sphincterotomy	17	2340	OR	PubMed, Web of Science, Embase, ClinicalTrials.gov, CENTRAL (January 1990-April 2022)	Jadad	Low
Martins, O. C. et al, 2024	PEP	Endoscopic sphincterotomy	Non-Endoscopic sphincterotomy	7	1022	OR	PubMed, Embase, Cochrane Library (December 2023)	RoB 2	Moderate
Matsubayashi, C. O. et al, 2020	Severe PEP	Endoscopic papillary balloon dilation	Endoscopic sphincterotomy	25	3476	RD	Embase, Medline/PubMed, CENTRAL (June 2019)	Modified tool based on RoB	Moderate
Mazaki, T. et al, 2014	Mild to Moderate PEP, PEP (High-risk patients)	Pancreatic stent placement	No Pancreatic stent placement	14	1541	RR	Cochrane Library, Medline, ProQuest (January 1974-December 2012)	Modified tool based on RoB	Low
Mutneja, H. R. et al, 2021	PEP	Needle knife fistulotomy	Conventional cannulation technique	4	582	OR	Embase, Medline, CINAHL, Google Scholar, CENTRAL (March 2020)	RoB	Low
Tang, Z. et al, 2018	PEP (Patients with difficult biliary access)	Early precut sphincterotomy	Persistent cannulation attempts	7	999	RR	PubMed, Web of Science, Embase, ScienceDirect (January 2017)	RoB	Low
Tringali, Alberto et al, 2019	PEP	Endoscopic papillary balloon dilation	Endoscopic sphincterotomy	25	3360	OR	Embase, Cochrane Library, Medline, CENTRAL (October 2017)	RoB	Low
Tse, F. et al, 2022	PEP, Mild PEP,Moderate PEP, Severe PEP	Guidewire-assisted cannulation	Contrast-assisted cannulation	15	4426	RR	Embase, Medline, ClinicalTrials.gov, CINAHL, CENTRAL, WHO ICTRP (February 2021)	RoB	High
Tse, F. et al, 2017	Mild PEP (Patients with difficult biliary cannulation), Moderate PEP (Patients with difficult biliary cannulation), Severe PEP (Patients with difficult biliary cannulation)	Double-guidewire technique	Other methods (Persistent attempts with conventional cannulation techniques, Precut sphincterotomy, Pancreatic duct stent placement)	7	577	RR	Embase (1974-April 2016), Medline (1946-April 2016), CENTRAL (Issue 3, 2016), CINAHL (1982-April 2016)	RoB	High
Verma, Dharmendra et al, 2007	PEP, Mild PEP,Moderate PEP, Severe PEP	Pure cut	Blend	4	804	NR	PubMed (January 1995-December 2005)	CONSORT	Critically low
Wang, L. et al, 2024	PEP (Patients with difficult biliary cannulation)	Double-guidewire technique	Other methods (Standard cannulation techniques, Pancreatic stent-assisted technique and Transpancreatic sphincterotomy technique)	20	2008	RR	PubMed, Embase, Cochrane Library, CNKI, Wanfang Data (December 2023)	RoB	Low
Yang, H. et al, 2022	PEP	Covered self-expandable metallic stents	Multiple plastic stents	6	444	OR	PubMed, Web of Science, Cochrane Library(January 2008-December 2021)	RoB and Jadad	Low
Zhu, G. et al, 2015	PEP, Moderate PEP	Pancreatic stent placement	No Pancreatic stent placement	14	1469	RR	PubMed, Embase (NR)	Jadad	Critically low

NSAIDs: Non-steroidal anti-inflammatory drugs

**Fig. 2:**
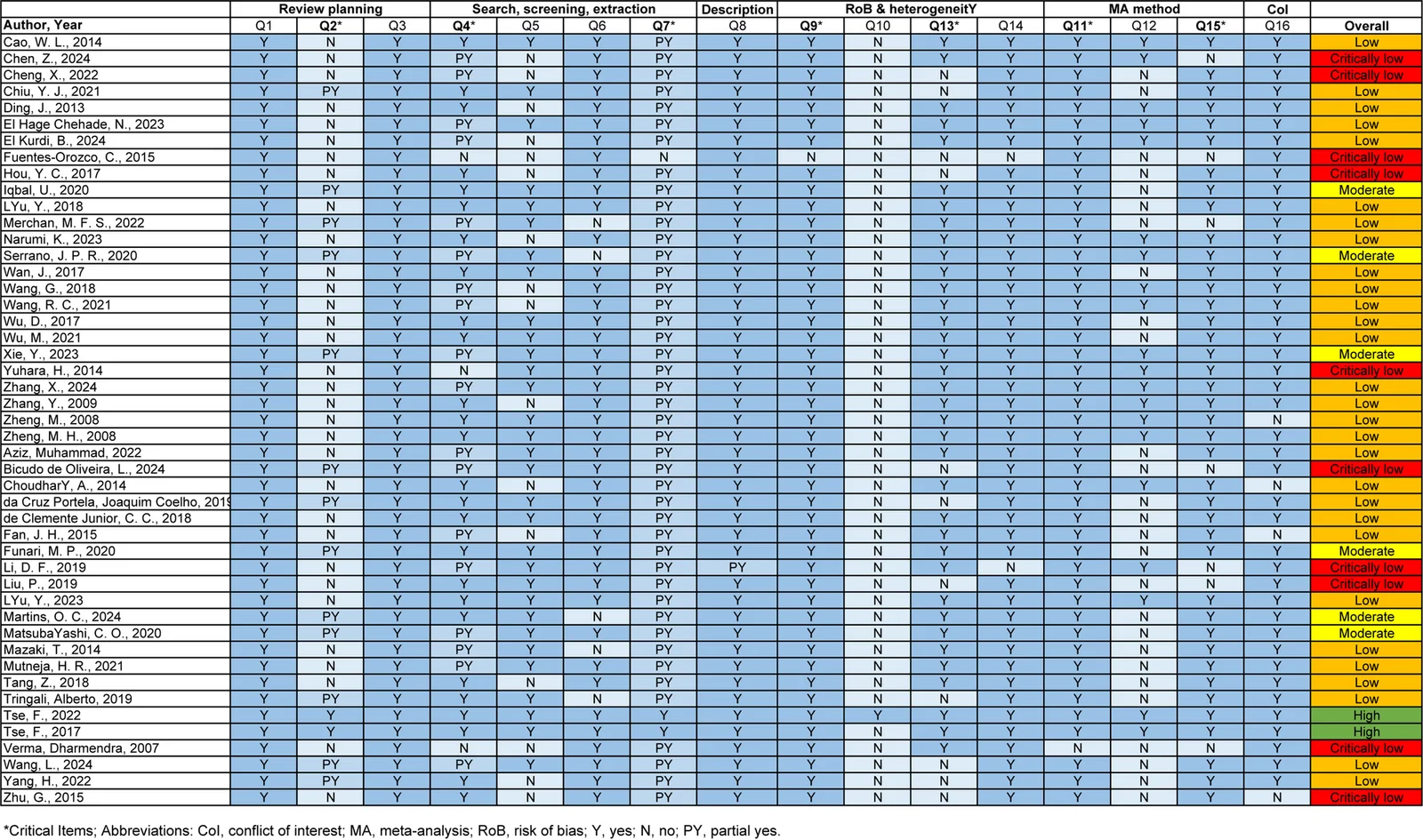
Methodological quality according to AMSTAR-2. High quality indicates zero or one non-critical weakness, while moderate quality demonstrates more than one non-critical weakness. Low quality indicates at least one critical flaw, with or without non-critical weaknesses, and critically low quality shows more than one critical flaw, with or without non-critical weaknesses. Details about the AMSTAR-2 can be found in the previously published study [16]

The results from the AMSTAR 2 revealed that 4% (2/47) had high quality, 13% (6/47) had moderate quality, 62% (29/47) had low quality, and 21% (10/47) had critically low quality. Detailed results of the AMSTAR 2 assessment for each study are presented in Fig. 2.

Based on the 47 articles included in this UR, 91 associations were identified. Overlapping meta-analyses were reported in 64 of these associations, with Additional file 1: Table S3 providing a citation matrix to assess the extent of this overlap. All 64 associations exhibited a high degree of overlap, as evidenced by CCA values exceeding 15% for each. According to our eligibility criteria, among the 64 associations with overlapping meta-analyses, only the single meta-analysis that included the largest number of studies was retained as the representative dataset for each association. Forest plots of the 91 associations between pharmacological interventions or procedural modifications and the incidence of PEP are presented in Additional file 1: Figs. S1-S91. Detailed re-analysis results and evidence credibility assessments for these 91 associations are provided in Table 3 and Additional file 1: Table S4. Among these, the strength of association was highly suggestive for one meta-analysis, suggestive for 13, weak for 25, and non-significant for 52 others. Additionally, the credibility of the original evidence remains unchanged when using RR as the single effect size, with detailed results provided in Additional file 1: Table S5. Furthermore, an assessment of the certainty of evidence using the GRADE framework revealed that 29% (26/91) of the associations were supported by moderate-quality evidence, while 47% (43/91) and 24% (22/91) were supported by low- and very-low-quality evidence, respectively. A detailed assessment for each association is presented in Additional file 1: Table S6.

### Incidence of PEP

In total, 31 meta-analyses examining the effects of PIs or PTMs on the incidence of PEP were based on 271 original RCTs, involving a total of 70,903 participants. Among the 31 interventions, 15 (48%) showed statistically significant results with a p-value < 0.05, 7 (23%) had p-values < 10⁻³, and 1 (3%) had a p-value < 10⁻⁶. Seven (23%) interventions exhibited high heterogeneity (I² > 50%). Only 2 interventions (6%) had a 95% prediction interval that excluded the null, and the largest study with a statistically significant effect was identified in 14 (45%) of the interventions. Of the 15 statistically significant interventions, 13 (87%) involved more than 1,000 participants, and 11 (73%) showed no small-study effects or excess significance bias.

**Fig. 3:**
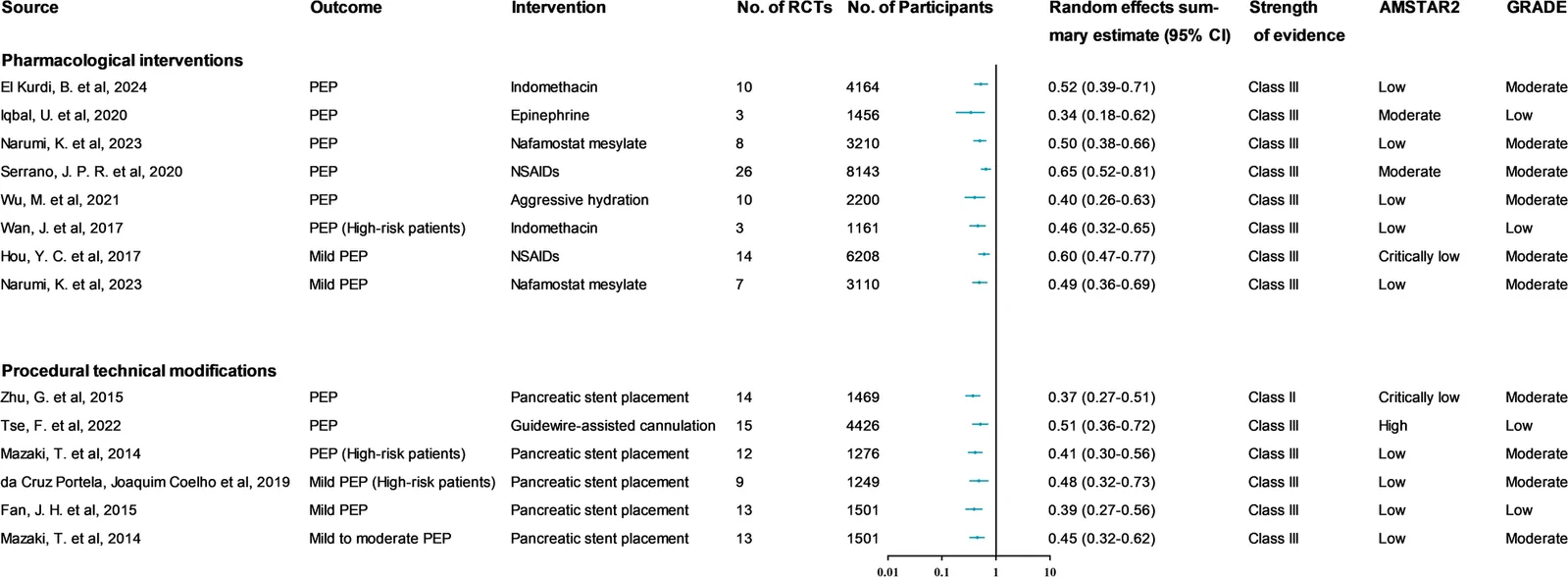
Forest plot of the association between pharmacological interventions or procedural technical modifications and the risk of PEP, with random effects summary estimates, strength of evidence, and AMSTAR-2 and GRADE quality assessments. Estimates are equivalent odds ratios or risk ratios, with corresponding 95% confidence intervals. A previously proposed umbrella review methodology was used to assess the strength of evidence [25]. PEP: Post endoscopic retrograde cholangiopancreatography pancreatitis; NSAIDs: nonsteroidal anti-inflammatory drugs; RCTs: randomized controlled trials

For PIs, five interventions were rated as suggestive evidence (Class III; Table 3; Fig. 3): indomethacin (OR 0.52, 95% CI 0.39–0.71), epinephrine (RR 0.34, 95% CI 0.18–0.62), nafamostat mesylate (RR 0.50, 95% CI 0.38–0.66), NSAIDs (RR 0.65, 95% CI 0.52–0.81), and aggressive hydration with Lactated Ringer Solution (OR 0.40, 95% CI 0.26–0.63). Notably, the 95% prediction intervals for both NSAIDs (95% PI: 0.28–1.50) and aggressive hydration (95% PI: 0.14–1.12) crossed the null value of 1.0, suggesting potential variation and uncertainty regarding their generalizability in future clinical settings.

For PTMs, one intervention was rated as highly suggestive evidence (Class II; Table 3; Fig. 3): pancreatic stent placement (RR 0.37, 95% CI 0.27–0.51). Notably, while this association demonstrated robust statistical credibility, the underlying meta-analysis by Zhu et al. (2015) was rated as critically low quality by AMSTAR 2 due to procedural omissions. To validate this finding, a re-analysis using data from Mazaki et al. (2014) was performed, confirming that the association robustly remained classified as Class II evidence (Additional file 1: Table S7). Additionally, one intervention was rated as suggestive evidence (Class III; Table 3; Fig. 3): guidewire-assisted cannulation (RR 0.51, 95% CI 0.36–0.72). Importantly, the 95% prediction interval for guidewire-assisted cannulation (0.21–1.26) encompassed the null threshold of 1.0, indicating potential inconsistency in its real-world efficacy.

Subgroup analysis revealed that the timing and route of NSAID administration significantly affect the incidence of PEP. Administering NSAIDs either before (RR 0.55, 95% CI 0.38–0.79) or after ERCP (RR 0.64, 95% CI 0.48–0.86) reduced the risk of PEP, with similar results observed for indomethacin. Diclofenac was effective only when administered before ERCP (RR 0.32, 95% CI 0.16–0.63). Apart from rectal NSAIDs (RR 0.57, 95% CI 0.44–0.75) and rectal diclofenac (RR 0.37, 95% CI 0.14–0.94), other routes of administration did not show any improvement in the occurrence of PEP. The analysis of nafamostat mesylate indicated that both the 20 mg (RR 0.50, 95% CI 0.35–0.73) and 50 mg (RR 0.43, 95% CI 0.26–0.72) doses were effective against PEP. For hydration regimens, only regimen A (RR 0.21, 95% CI 0.10–0.43) significantly reduced the incidence of PEP. Additionally, operator expertise during guidewire-assisted cannulation had a substantial impact on PEP rates. ERCP performed by experienced endoscopists (RR 0.34, 95% CI 0.20–0.58) exhibited a stronger preventive effect compared to procedures involving trainees (RR 0.93, 95% CI 0.60–1.46). Detailed results are provided in Additional file 1: Table S8.

### Incidence of PEP in high-risk patients

In total, 16 meta-analyses assessed the incidence of PEP for high-risk patients based on 98 original RCTs, including a total of 14,616 participants. Of the 16 interventions, 9 (56%) showed statistically significant results with a p-value < 0.05, 3 (19%) had p-values < 10⁻³, and 1 (6%) had a p-value < 10⁻⁶. Two (13%) interventions exhibited high heterogeneity (I² > 50%). The 95% prediction interval excluded the null hypothesis in only 3 (19%) of the interventions, and the largest study with a statistically significant effect was identified in 3 (19%) interventions. Among the 9 statistically significant interventions, 7 (78%) included over 1,000 participants, and 5 (56%) showed no evidence of small-study effects or excess significance bias.

In the context of PIs to prevent PEP in high-risk patients, indomethacin (RR 0.46, 95% CI 0.32–0.65) was classified as suggestive evidence (Class III; Table 3; Fig. 3). Concurrently, a markedly wide 95% prediction interval spanning the null value (0.05–4.13) was observed for this intervention, indicating a low level of statistical precision for future applications. Regarding PTMs, pancreatic stent placement was classified as suggestive evidence for preventing PEP (RR 0.41, 95% CI 0.30–0.56) or mild PEP (RR 0.48, 95% CI 0.32–0.73) in high-risk patients (Class III; Table 3; Fig. 3).

### Incidence of mild PEP

In total, 12 meta-analyses evaluating the effects of PIs or PTMs on the incidence of mild PEP included 83 original RCTs, with a total of 28,252 participants. Of the 12 interventions, 7 (58%) were statistically significant with a p-value < 0.05, 3 (25%) had p-values < 10⁻³, and 1 (8%) had a p-value < 10⁻⁶. None of the interventions exhibited high heterogeneity (I² > 50%). In 2 (17%) interventions, the 95% prediction interval excluded the null effect, and in 4 (33%), the largest study with a statistically significant effect was identified. Among the 7 statistically significant interventions, 6 (86%) involved more than 1,000 participants, and 4 (57%) showed no evidence of small study effects or excess significance bias.

For PIs, two interventions were classified as suggestive evidence (Class III; Table 3; Fig. 3): NSAIDs (RR 0.60, 95% CI 0.47–0.77) and nafamostat mesylate (RR 0.49, 95% CI 0.36–0.69). Among PTMs, pancreatic stent placement (OR 0.39, 95% CI 0.27–0.56) was classified as suggestive evidence (Class III; Table 3; Fig. 3).

**Table 3:** Association between pharmacological interventions or procedural technical modifications and the incidence of PEP, graded as highly suggestive evidence (Class II), suggestive evidence (Class III), or weak evidence (Class IV)

Author, year	Intervention	Comparator	Outcome	Studies (Participants)	Effect metrics	Random effects summary estimate (95% Cl)	Random effects p value	I^2^ (%)	95% prediction interval	Random effects estimate of the largest study (95% Cl)	Egger's test p value	Signifcant studies			AMSTAR2
												Observed	Expected	p value	
**Highly suggestive (Class II)**															
Zhu, G. et al, 2015	Pancreatic stent placement	No Pancreatic stent placement	PEP	14 (1469)	RR	0.37 (0.27-0.51)	5.6 x 10^-10^	0%	0.26-0.52	0.52 (0.29-0.92)	0.001	6	5.54	0.79	Critically low
**Suggestive (Class III)**															
**Pharmacological prevention**															
El Kurdi, B. et al, 2024	Indomethacin	No NSAIDs	PEP	10 (4164)	OR	0.52 (0.39-0.71)	2.9 x 10^-5^	38.6%	0.25-1.12	0.49 (0.30-0.81)	0.56	6	9.16	NP	Low
Iqbal, U. et al, 2020	Epinephrine	Placebo/No treatment	PEP	3 (1456)	RR	0.34 (0.18-0.62)	3.1 x 10^-4^	0%	0.01-15.99	0.30 (0.15-0.63)	0.80	1	2.98	NP	Moderate
Narumi, K. et al, 2023	Nafamostat mesylate	Placebo/No treatment	PEP	8 (3210)	RR	0.50 (0.38-0.66)	1.1 x 10^-6^	0%	0.35-0.71	0.35 (0.20-0.62)	0.89	4	7.61	NP	Low
Serrano, J. P. R. et al, 2020	NSAIDs	Placebo SN/Placebo lactated Ringer’s solution/Ceftazidime/Glycerin/Glyceryl trinitrate/Saline solution /No treatment	PEP	26 (8143)	RR	0.65 (0.52-0.81)	1.1 x 10^-4^	52.9%	0.28-1.50	0.54 (0.35-0.84)	0.19	11	19.13	NP	Moderate
Wu, M. et al, 2021	Aggressive hydration with Lactated Ringer Solution	Standard hydration with Lactated Ringer Solution	PEP	10 (2200)	OR	0.40 (0.26-0.63)	5.2 x 10^-5^	30.5%	0.14-1.12	0.37 (0.18, 0.76)	0.53	5	7.81	NP	Low
Wan, J. et al, 2017	Indomethacin	Placebo	PEP (High-risk patients)	3 (1161)	RR	0.46 (0.32-0.65)	6.9 x 10^-6^	3.9%	0.05-4.13	0.54 (0.35-0.84)	0.05	3	2.49	1.00	Low
Hou, Y. C. et al, 2017	NSAIDs	Placebo/Glycerin/No treatment	Mild PEP	14 (6208)	RR	0.60 (0.47-0.77)	7.1 x 10^-5^	18.9%	0.35-1.02	0.47 (0.29-0.78)	0.68	3	12.05	NP	Critically low
Narumi, K. et al, 2023	Nafamostat mesylate	Placebo/No treatment	Mild PEP	7 (3110)	RR	0.49 (0.36-0.69)	2.6 x 10^-5^	0%	0.32-0.76	0.46 (0.25-0.84)	0.63	3	6.32	NP	Low
Procedural technical modifications															
Tse, F. et al, 2022	Guidewire-assisted cannulation	Contrast-assisted cannulation	PEP	15 (4426)	RR	0.51 (0.36-0.72)	1.3 x 10^-4^	35.5%	0.21-1.26	1.23 (0.61-2.50)	0.04	4	2.44	0.29	High
Mazaki, T. et al, 2014	Pancreatic stent placement	No Pancreatic stent placement	PEP (High-risk patients)	12 (1276)	RR	0.41 (0.30-0.56)	2.5 x 10^-8^	0%	0.29-0.59	0.65 (0.38-1.10)	0.000	6	2.65	0.03	Low
da Cruz Portela, Joaquim Coelho et al, 2019	Pancreatic stent placement	No Pancreatic stent placement	Mild PEP (High-risk patients)	9 (1249)	RR	0.48 (0.32-0.73)	5.3 x 10^-4^	0%	0.29-0.79	0.52 (0.26-1.02)	0.25	1	4.44	NP	Low
Fan, J. H. et al, 2015	Pancreatic stent placement	No Pancreatic stent placement	Mild PEP	13 (1501)	OR	0.39 (0.27-0.56)	6.5 x 10^-7^	0%	0.26-0.60	0.63 (0.35-1.15)	0.004	4	3.41	0.75	Low
Mazaki, T. et al, 2014	Pancreatic stent placement	No Pancreatic stent placement	Mild to moderate PEP	13 (1501)	RR	0.45 (0.32-0.62)	8.0 x 10^-7^	0%	0.31-0.64	0.67 (0.39-1.14)	0.003	4	2.77	0.49	Low
**Weak (Class lV)**															
**Pharmacological prevention**															
Chiu, Y. J. et al, 2021	Gabexate mesilate	Placebo/No treatment	PEP	12 (3662)	RR	0.56 (0.37-0.86)	7.8 x 10^-3^	40.8%	0.18-1.70	1.20 (0.66-2.18)	0.02	3	1.71	0.40	Low
Ding, J. et al, 2013	Glyceryl Trinitrate	Placebo/Physiological saline/Vitamin C/No intervention	PEP	11 (2395)	RR	0.68 (0.51-0.90)	6.5 x 10^-3^	5.1%	0.46-1.01	0.63 (0.35-1.11)	0.77	1	4.47	NP	Low
Merchan, M. F. S. et al, 2022	Antibiotics	Non-antibiotics	PEP	4 (698)	RR	0.51 (0.27-0.97)	3.9 x 10^-2^	0%	0.13-2.13	0.69 (0.26-1.80)	0.64	1	1.05	NP	Low
Serrano, J. P. R. et al, 2020	Diclofenac	Placebo SN/Placebo lactated Ringer’s solution/Ceftazidime/Glycerin/Glyceryl trinitrate/Saline solution /No treatment	PEP	14 (3501)	RR	0.70 (0.49-0.98)	3.8 x 10^-2^	59.6%	0.23-2.08	0.60 (0.38-0.93)	0.08	5	7.40	NP	Moderate
Wang, G. et al, 2018	Somatostatin	Placebo/No treatment	PEP	14 (4593)	OR	0.60 (0.41-0.89)	1.1 x 10^-2^	55.8%	0.18-2.03	0.51 (0.28-0.92)	0.16	5	11.43	NP	Low
Zhang, X. et al, 2024	Glyceryl Trinitrate + NSAIDs	NSAIDs	PEP	3 (1254)	RR	0.53 (0.36-0.78)	1.4 x 10^-3^	0%	0.04-6.62	0.59 (0.37-0.95)	0.30	2	1.92	1.00	Low
Lyu, Y. et al, 2018	NSAIDs	Placebo	PEP (High-risk patients)	6 (1439)	RR	0.54 (0.31-0.94)	2.8 x 10^-2^	62.8%	0.10-2.79	0.54 (0.35-0.84)	0.63	4	3.89	1.00	Low
Wang, G. et al, 2018	Somatostatin	Placebo/No treatment	PEP (High-risk patients)	3 (1114)	OR	0.62 (0.41-0.93)	2.3 x 10^-2^	0%	0.04-9.18	0.56 (0 30-1.03)	NA	0	1.92	NP	Low
Xie, Y. et al, 2023	Nafamostat mesylate	Placebo/No treatment	PEP (High-risk patients)	6 (1294)	RR	0.57 (0.35-0.94)	2.7 x 10^-2^	40.6%	0.16-2.07	0.99 (0.53-1.87)	0.25	2	0.30	0.03	Moderate
Chen, Z. et al, 2024	Epinephrine	Placebo/No treatment	Mild PEP	2 (1311)	RR	0.26 (0.11-0.60)	1.8 x 10^-3^	0%	NA	0.27 (0.11-0.66)	NA	1	2.00	NP	Critically low
Wan, J. et al, 2017	Indomethacin	Placebo	Mild PEP	7 (3013)	RR	0.61 (0.40-0.93)	2.2 x 10^-2^	50.0%	0.19-1.95	0.45 (0.25-0.81)	0.53	3	6.56	NP	Low
Serrano, J. P. R. et al, 2020	NSAIDs	Placebo SN/Placebo lactated Ringer’s solution/Ceftazidime/Glycerin/Glyceryl trinitrate/Saline solution /No treatment	Moderate PEP	11 (4284)	RR	0.62 (0.44-0.89)	9.5 x 10^-3^	3.5%	0.40-0.99	0.63 (0.33-1.17)	0.92	1	7.18	NP	Moderate
El Kurdi, B. et al, 2024	NSAIDs	No NSAIDs	Moderate to severe PEP	19 (6281)	OR	0.57 (0.40-0.81)	1.7 x 10^-3^	0%	0.40-0.84	0.48 (0.24-0.95)	0.75	1	16.63	NP	Low
El Kurdi, B. et al, 2024	Indomethacin	No NSAIDs	Moderate to severe PEP	8 (3608)	OR	0.50 (0.30-0.81)	5.4 x 10^-3^	0%	0.27-0.93	0.48 (0.24-0.95)	0.71	1	7.57	NP	Low
Wu, D. et al, 2017	Aggressive hydration with Lactated Ringer Solution	Standard hydration with Lactated Ringer Solution	Moderate to severe PEP	3 (722)	OR	0.38 (0.25-0.59)	8.2 x 10^-6^	0%	0.02-5.91	0.37 (0.21-0.68)	0.22	2	2.46	NP	Low
**Procedural technical modifications**															
Bicudo de Oliveira, L. et al, 2024	Endocut	Pure cut	PEP	4 (987)	RR	3.00 (1.40-6.45)	4.9 x 10^-3^	0%	0.56-16.04	2.61 (1.04-6.57)	0.12	1	3.31	NP	Critically low
Yang, H. et al, 2022	Covered self-expandable metallic stents	Multiple plastic stents	PEP	4 (1154)	OR	3.17 (1.18-8.52)	2.2 x 10^-2^	11.6%	0.23-44.37	7.08 (1.81-27.70)	0.80	1	4.00	NP	Low
Tang, Z. et al, 2018	Early precut sphincterotomy	Persistent cannulation attempts	PEP (Patients with difficult biliary access)	7 (999)	RR	0.57 (0.36-0.92)	2.1 x 10^-2^	0%	0.31-1.06	0.45 (0.22-0.91)	0.92	2	4.38	NP	Low
da Cruz Portela, Joaquim Coelho et al, 2019	Pancreatic stent placement	No Pancreatic stent placement	Moderate PEP (High-risk patients)	8 (1129)	RR	0.35 (0.17-0.72)	4.8 x 10^-3^	0%	0.14-0.89	0.48 (0.15-1.56)	0.22	0	4.53	NP	Low
da Cruz Portela, Joaquim Coelho et al, 2019	Pancreatic stent placement	No Pancreatic stent placement	Severe PEP (High-risk patients)	7 (932)	RR	0.31 (0.10-0.97)	4.5 x 10^-2^	0%	0.07-1.39	0.14 (0.01-2.53)	0.27	0	6.98	NP	Low
Bicudo de Oliveira, L. et al, 2024	Endocut	Pure cut	Mild PEP	4 (987)	RR	2.53 (1.24-5.16)	1.0 x 10^-2^	0%	0.53-12.14	2.52 (0.97-6.52)	0.29	0	3.24	NP	Critically low
Tse, F. et al, 2022	Guidewire-assisted cannulation	Contrast-assisted cannulation	Mild PEP	11 (3306)	RR	0.47 (0.26-0.83)	9.8 x 10^-3^	49.0%	0.10-2.24	1.44 (0.63-3.31)	0.08	3	4.50	NP	High
Zhu, G. et al, 2015	Pancreatic stent placement	No Pancreatic stent placement	Moderate PEP	8 (1087)	RR	0.37 (0.17-0.79)	1.0 x 10^-2^	0%	0.14-0.96	0.52 (0.16-1.72)	0.08	0	3.78	NP	Critically low
Fan, J. H. et al, 2015	Pancreatic stent placement	No Pancreatic stent placement	Severe PEP	8 (1009)	OR	0.25 (0.08-0.75)	1.3 x 10^-2^	0%	0.06-0.96	0.12 (0.01-2.37)	0.000	0	7.92	NP	Low
Matsubayashi, C. O. et al, 2020	Endoscopic papillary balloon dilation	Endoscopic sphincterotomy	Severe PEP	6 (1019)	RR	3.32 (1.07-10.29)	3.7 x 10^-2^	0%	0.67-16.47	2.00 (0.19-21.62)	0.33	0	4.04	NP	Moderate

*NA* not available

*NP* not pertinent (because the number of expecied significan studies was larger than the number of observed significant studies)

### Incidence of moderate PEP, or severe PEP

Only two interventions were rated as weak evidence for preventing moderate PEP, while two PTMs were classified as weak evidence for preventing severe PEP, as detailed in Additional file 1: Results S1 and Results S2.

### Incidence of mild to moderate PEP or moderate to severe PEP

There was 1 meta-analysis that evaluated the effect of interventions on the incidence of mild to moderate PEP and 9 meta-analyses that assessed the incidence of moderate to severe PEP. The 9 meta-analyses, which focused on the effects of PIs on moderate to severe PEP, were based on 60 original RCTs involving 22,902 participants. Of the 9 interventions assessed, 3 (33%) showed statistically significant results with a p-value < 0.05, and 1 (11%) demonstrated a p-value < 10⁻³. None of the interventions exhibited high heterogeneity (I² > 50%). Only 2 (22%) interventions had a 95% prediction interval that excluded the null, and the largest study with a statistically significant effect was identified in 3 (33%) of the interventions. Among the 3 statistically significant interventions, 2 (67%) involved more than 1,000 participants, and all (100%) showed no evidence of small-study effects or excess significance bias.

For the prevention of mild to moderate PEP, pancreatic stent placement (RR 0.45, 95% CI 0.32–0.62) was classified as suggestive evidence (Class III; Table 3; Fig. 3).

## Discussion

This UR provides a comprehensive synthesis of meta-analytic evidence evaluating the effectiveness of PIs and PTMs in preventing PEP across various clinical settings (Graphical abstract). Although the pathophysiology of PEP is not yet fully elucidated, two predominant mechanisms have been implicated: (1) bile reflux into the pancreatic duct due to transient ampullary obstruction during ERCP and (2) direct mechanical injury or edema of the ampulla from endoscopic instrumentation [68, 69]. Most current preventive strategies are designed to address these underlying mechanisms. However, individual RCTs and meta-analyses often focus on specific interventions, making it difficult to determine the most effective approach. The graded evidence from this UR supports a shift from a one-size-fits-all approach to a risk-stratified, potentially combined prevention strategy. For average-risk patients, a high-evidence pharmacological measure (e.g., rectal indomethacin) forms the cornerstone. In contemporary clinical practice, a high-risk setting involves either patient-inherent factors (such as a history of PEP or suspected SOD) or procedural challenges (such as difficult cannulation, repeated pancreatic duct access, or precut sphincterotomy). For these high-risk patients and settings, our data support a combined multimodal strategy: prophylactic pancreatic stent placement (Class II/III) should be considered on a foundation of pharmacological prophylaxis (indomethacin, Class III) to combat both patient-related susceptibility and procedure-induced mechanical trauma. In cases of anticipated or encountered difficult cannulation, guidewire-assisted cannulation (Class III) adds a procedural layer of protection. Notably, the 95% prediction interval for guidewire-assisted cannulation (0.21–1.26) crosses the null, highlighting that its clinical generalizability is constrained by variations in operator expertise. Our findings may provide key evidence to inform future updates of clinical guidelines.

Compared with other interventions, prophylactic pancreatic stent placement demonstrated a higher level of evidence credibility for preventing both overall and severe PEP in unselected and high-risk patients. Despite this, its routine use in clinical practice has declined over the past decade due to concerns about technical complexity, procedure-related risks, and inconsistent outcomes [12, 70, 71]. Importantly, failed stent placement may increase the risk of PEP beyond baseline levels [72]. Nevertheless, based on our findings, and particularly given its role in reducing severe PEP, prophylactic stenting may be justified even in cases where initial ductal access is not established, especially in high-risk patients. This highlights the need to revisit clinical criteria for selecting candidates for stent placement. Furthermore, this clinical selection must be balanced against methodological reality, as this headline Class II evidence is derived from a meta-analysis with a critically low AMSTAR 2 rating, indicating compromised reporting transparency despite robust empirical data. Therefore, these high-level evidence findings should be interpreted with caution, and pancreatic stenting should be deployed as a selective, risk-stratified strategy rather than a universal mandate.

NSAIDs represent another widely endorsed preventive strategy. Both indomethacin and diclofenac have been investigated extensively, though their efficacy varies depending on the patient risk profile and administration protocols [11, 73]. In our review, indomethacin showed suggestive evidence for preventing both mild and moderate-to-severe PEP, while diclofenac demonstrated only weak evidence. However, for NSAIDs as a broader category, the 95% prediction interval crosses the null (95% PI = 0.28–1.50). Similarly, for indomethacin in high-risk patients, the prediction interval is markedly wide (95% PI = 0.05–4.13). These statistical caveats imply that their protective benefits could be absent or significantly attenuated in certain future clinical cohorts due to underlying clinical heterogeneity. This gap underscores the need for continued education and system-level interventions to improve uptake.

Therefore, balancing the clinical roles of these procedural and pharmacological strategies is crucial. While our umbrella review assigns a high evidence credibility classification to prophylactic pancreatic stent placement (Class II/III), we must distinguish statistical grading from real-world clinical execution. In contemporary ERCP practice, rectal NSAIDs (such as indomethacin) represent the true routine foundation of PEP prophylaxis because of their simplicity, broad safety profile, low cost, and lack of technical barriers. Conversely, pancreatic stenting is inherently limited by its high dependence on advanced technical expertise, and failed attempts can paradoxically exacerbate pancreatic injury. Therefore, rather than serving as a universal preventive measure, pancreatic stenting should be deployed selectively as part of a risk-stratified, multimodal strategy. Specifically, stenting should be layered on top of foundational pharmacological prophylaxis in high-risk procedural scenarios, such as extended or difficult cannulation, repeated accidental guidewire passage into the pancreatic duct, contrast injection into the pancreatic duct, or the utilization of transpancreatic techniques.

Aggressive intravenous hydration is another key preventive measure, particularly in the United States, where it is administered in more than 80% of ERCP procedures [74]. However, optimal hydration protocols, including the type of fluid, infusion volume, and timing, remain subjects of ongoing investigation. Moreover, the risk of fluid overload and the extended duration required in emergency ERCP settings must be carefully considered when applying this intervention. Importantly, aggressive hydration with Lactated Ringer’s solution also exhibited a 95% prediction interval spanning the null (95% PI = 0.14–1.12). This suggests that its real-world clinical benefit might be inconsistent unless standardized, optimal hydration protocols are strictly and universally implemented.

Nafamostat mesylate, approved in South Korea and Japan for the treatment of acute pancreatitis [75], has also emerged as a potential preventive agent for PEP. Nonetheless, its clinical use warrants caution due to AEs such as hyperkalemia [76] and phlebitis [77], which necessitate close monitoring. While nafamostat mesylate demonstrated preventive efficacy with suggestive evidence (Class III) in our re-analysis, the global generalizability of such region-specific agents—including ulinastatin and gabexate mesylate—warrants considerable caution. Their lack of formal endorsement by Western regulatory agencies (e.g., the FDA or EMA) or clinical guidelines (e.g., those of the ASGE or ESGE) inherently limits their worldwide applicability, restricting their routine clinical deployment predominantly to East Asian countries.

Topical epinephrine administration may reduce papillary edema by decreasing capillary permeability and relaxing the sphincter of Oddi [78]. In contrast, thermal injury from electrosurgical cutting (Endocut) can exacerbate edema at the major duodenal papilla [79, 80]. The combined use of topical epinephrine with Endocut may offer a synergistic effect in patients at risk of coagulopathy, but further RCTs are needed to validate this approach.

Somatostatin and its analogs, known for their inhibitory effect on pancreatic enzyme secretion, may also exert anti-inflammatory and cytoprotective effects [81]. Although a multicenter RCT conducted by Bai et al. [82] showed positive outcomes, subsequent trials have reported conflicting results [83, 84]. Due to the overall low level of supporting evidence, the role of somatostatin in routine PEP prevention remains uncertain and requires further clarification.

Endoscopic papillary balloon dilation (EPBD) was identified as a risk factor for severe PEP, likely due to compartment syndrome caused by post-dilation hemorrhage and edema in patients with an intact sphincter of Oddi [85, 86]. The duration of EPBD is a clinically relevant variable, with current guidelines recommending at least 2 min of dilation [87]. Shorter durations have been associated with a higher incidence of PEP [88, 89], but whether longer durations reduce the risk remains an open question.

Some NMAs also provided results on optimal interventions to prevent PEP. Njel et al. [90] concluded that pancreatic stent placement is the most effective strategy for preventing PEP, with 29 trials and 7,862 participants included. Only the efficacy of rectal NSAIDs, aggressive hydration, and pancreatic stent placement was analyzed in this NMA. Another NMA conducted by Park et al. [91] concluded that rectal NSAID-based combination regimens-particularly those combined with aggressive hydration or nitrates-are more effective than single-modality approaches for preventing PEP. Compared with URs, NMAs can be used to assess the relative effectiveness of different interventions for PEP prevention and rank them accordingly. By contrast, URs are more suitable for the comprehensive integration of evidence on all evaluated interventions and the clear classification of evidence strength levels. Due to methodological limitations, we excluded NMAs from our analysis, which may have restricted our ability to draw comparative inferences about interventions that have not been directly compared in pairwise meta-analyses.

Our evidence-graded findings align with key recommendations from major guidelines [6, 15]. Our analysis extends beyond current guidance by identifying suggestive evidence for other interventions (e.g., nafamostat, epinephrine, aggressive hydration) that are not prominently featured in existing guidelines. Importantly, both our review and the guidelines highlight a consistent lack of convincing (Class I) evidence for any single strategy, underscoring the need for more robust trials. Our systematic credibility assessment offers a nuanced evidence hierarchy that may inform future guideline updates. Moreover, the quality of evidence in this umbrella review was assessed using both the Credibility and GRADE tools, thereby enhancing the objectivity of the findings.

While this review focused on efficacy, the safety profiles of interventions are crucial for clinical decision-making. Rectal NSAIDs may pose risks of renal impairment or gastrointestinal bleeding in vulnerable patients, and aggressive hydration can lead to volume overload [92]. Pancreatic stent placement is technically challenging and carries risks such as stent-induced ductal injury, migration, or occlusion. Failed stent placement may paradoxically increase PEP risk [72]. A comprehensive risk-benefit assessment, considering patient-specific factors, is therefore essential when selecting prophylactic strategies.

Despite applying rigorous inclusion criteria and standardized credibility assessments, several limitations of this UR should be acknowledged. First, although we included only meta-analyses based on RCTs, residual bias from the original studies cannot be entirely excluded. Methodological details such as randomization, blinding, and sample size justification were not uniformly reported and fall outside the scope of this review to verify. Second, the efficacy of PEP prevention may vary depending on specific variables such as dosage, timing, and combination of PIs, as well as procedural nuances in PTMs [38, 93–96]. Third, we excluded systematic reviews that did not perform quantitative synthesis, which may limit the comprehensiveness of our evaluation. Fourth, most supportive evidence was graded as only Class II or III, warranting cautious interpretation. Fifth, a critical internal inconsistency must be noted as the single intervention that reached Class II evidence, namely pancreatic stent placement, was based on a meta-analysis with a critically low AMSTAR 2 quality rating. This scientific paradox highlights that a high level of data credibility does not necessarily equate to ideal methodological conduct in the systematic review process. Although a validation re-analysis using an alternative review by Mazaki et al. 2014 yielded identical Class II results, readers should remain cognizant of this limitation when translating these findings into clinical guidelines. Furthermore, we must acknowledge the temporal heterogeneity of the included evidence as an important nuance in clinical translation. A substantial portion of the primary RCTs synthesized in older meta-analyses was completed prior to the global implementation of rectal NSAIDs and contemporary combined prophylactic strategies. Certain historical interventions may appear markedly significant in these pooled data largely because they were tested against a baseline lacking today’s advanced, highly effective preventive measures. Because modern ERCP practice relies on a multi-pronged, risk-stratified standard of care, the relative clinical efficacy and absolute risk reduction demonstrated by these older standalone strategies may be significantly attenuated today.

## Conclusions

This UR highlights pancreatic stent placement as an effective intervention for PEP prevention, supported by highly suggestive evidence in the general population and suggestive evidence in high-risk patients. Indomethacin and several additional strategies—including epinephrine, nafamostat mesylate, NSAIDs, aggressive hydration, and guidewire-assisted cannulation—demonstrated suggestive pooled efficacy across different patient groups. However, because the 95% prediction intervals for these Class III interventions cross the null or are markedly wide, their true clinical generalizability remains uncertain, and their protective benefits may be inconsistent across varying clinical settings due to underlying heterogeneity (such as variations in NSAID dosing, hydration volumes, and operator expertise). Overall, the current evidence base primarily consists of Class II–III evidence, with no interventions achieving Class I evidence. To strengthen clinical guidelines and optimize prevention strategies for PEP, future high-quality, well-powered RCTs with strict, standardized intervention protocols are essential to definitively narrow these prediction intervals and resolve these clinical uncertainties.

## Supplementary Information

Below is the link to the electronic supplementary material.

Supplementary Material 1: Additional file 1: Results S1-S2, Table S1-S8, Figs. Figures S1-S91.

Supplementary Material 2

Supplementary Material 3

## Data Availability

All data generated or analyzed during this study is included in this published article and its supplementary information files. As this umbrella review used only study-level summary statistics from published studies, no individual patient data was involved and no restrictions on data access apply.

## Abbreviations

AEs: adverse events
AP: acute pancreatitis
CI: confidence interval
ERCP: post-endoscopic retrograde cholangiopancreatography
EPBD: endoscopic papillary balloon dilation
OR: odds ratio
PEP: post-endoscopic retrograde cholangiopancreatography pancreatitis
PIs: pharmacological interventions
PTMs: procedural technical modifications
RCTs: randomized controlled trials
RR: risk ratio
RD: risk difference
ROB: risk of bias
UR: umbrella review
