# Supplementary material for: Pharmacological and procedural strategies to prevent post-ERCP pancreatitis: an umbrella review of meta-analyses of randomized controlled trials
Source: BMC Med. 2026 Aug 6;24:613. doi: 10.1186/s12916-026-05115-0 (PMC13729418; doi:10.1186/s12916-026-05115-0)
Supplement: Supplementary file 2 [file 12916_2026_5115_MOESM2_ESM.docx]

**Additional file 1**

**Results S1**. Incidence of moderate PEP. (Page 2)

**Results S2.** Incidence of severe PEP. (Page 2)

**Table S1.** Search strategy. (Page 3)

**Table S2.** List of excluded studies, with reasons for exclusion after full-text reading. (Pages 4-19)

**Table S3.** Corrected Covered Area Assessment of 64 associations. (Pages 20-75)

**Table S4.** Association between pharmacological interventions or procedural technical modifications and the incidence of PEP, graded as not significant. (Pages 76-79)

**Table S5.** Association between pharmacological interventions or procedural technical modifications and the incidence of PEP: a standardized analysis using relative risk. (Pages 80-86)

**Table S6.** Quality assessment of evidence using the GRADE framework. (Pages 87-105)

**Table S7.** Re-evaluation of the evidence credibility for the association between pancreatic stent placement and the incidence of PEP using a meta-analysis with higher methodological quality (Mazaki et al., 2014). (Page 106)

**Table S8.** Subgroup Analysis of timing, route, dose, regimen, and operator expertise in highly suggestive and suggestive evidence. (Pages 107-108)

**Figures S1-S91.** Forest plots of 91 associations between pharmacological interventions or procedural technical modifications and the incidence of PEP. (Pages 109-136)

**Results S1**

**Incidence of moderate PEP**

In total, 10 meta-analyses evaluating the effects of PIs and PTMs on the incidence of moderate PEP included 59 original RCTs with a total of 18,696 participants. Among the 10 interventions, only 2 (20%) were statistically significant with p-values < 0.05, and none had a p-value < 10⁻³. One intervention (10%) exhibited high heterogeneity (I² > 50%). The 95% prediction interval excluded the null in only 2 interventions (20%), and the largest study of all interventions showed no statistically significant effect. Both statistically significant interventions included more than 1,000 participants, and one (50%) showed no evidence of small study effects or excess significance bias.

Only two interventions were rated as weak evidence for preventing moderate PEP (Class IV; Table 2; Figure 3): NSAIDs (RR 0.62, 95% CI 0.44-0.89) and pancreatic stent placement (RR 0.37, 95% CI 0.17-0.79). The other interventions demonstrated no significant results.

**Results S2**

**Incidence of severe PEP**

In total, 12 meta-analyses assessed the impact of PIs and PTMs on the incidence of severe PEP, including 57 original RCTs with a total of 19,734 participants. Of the 12 interventions assessed, only 2 (17%) showed statistically significant results with p-values < 0.05, and none had p-values < 10⁻³. No interventions exhibited high heterogeneity (I² > 50%). The 95% prediction interval excluded the null in just 1 (8%) intervention, and the largest study of all interventions showed no statistically significant effect. Both statistically significant interventions involved over 1,000 participants, and 1 (50%) showed no small-study effects or excess significance bias.

Among the interventions, only two PTMs were graded as weak evidence (Class IV; Table 2; Figure 3): pancreatic stent placement (OR 0.25, 95% CI 0.08-0.75) and EPBD (vs. endoscopic sphincterotomy, RR 3.32, 95% CI 1.07-10.29). All the other interventions showed no significant results.

**Table S1.** Search strategy.

| **PubMed** | |
| --- | --- |
| #1 | "cholangiopancreatography, endoscopic retrograde"[MeSH Terms] OR ("cholangiopancreatography"[All Fields] AND "endoscopic"[All Fields] AND "retrograde"[All Fields]) OR "endoscopic retrograde cholangiopancreatography"[All Fields] OR ("endoscopic"[All Fields] AND "retrograde"[All Fields] AND "cholangiopancreatography"[All Fields]) OR ("cholangiopancreatography, endoscopic retrograde"[MeSH Terms] OR ("cholangiopancreatography"[All Fields] AND "endoscopic"[All Fields] AND "retrograde"[All Fields]) OR "endoscopic retrograde cholangiopancreatography"[All Fields] OR "ercp"[All Fields]) |
| #2 | "pancreas"[MeSH Terms] OR "pancreas"[All Fields] OR "pancreatic"[All Fields] OR "pancreatitides"[All Fields] OR "pancreatitis"[MeSH Terms] OR "pancreatitis"[All Fields] |
| #3 | "systematic review"[Publication Type] OR "systematic reviews as topic"[MeSH Terms] OR "systematic review"[All Fields] OR "meta analysis"[Publication Type] OR "meta analysis as topic"[MeSH Terms] OR "meta analysis"[All Fields] |
| #4 | Combine: #1 AND #2 AND #3 |
| **Web of Science** | |
| #1 | TS=(endoscopic retrograde cholangiopancreatography OR ERCP) |
| #2 | TS=(pancreatitis) |
| #3 | TS=(systematic review OR meta-analysis) |
| #4 | Combine: #1 AND #2 AND #3 |
| **Embase** | |
| #1 | 'endoscopic retrograde cholangiopancreatography'/exp OR 'endoscopic retrograde cholangiopancreatography' OR (endoscopic AND retrograde AND ('cholangiopancreatography'/exp OR cholangiopancreatography)) OR 'ercp'/exp OR ercp |
| #2 | 'pancreatitis'/exp OR pancreatitis |
| #3 | 'systematic review'/exp OR 'systematic review' OR (systematic AND ('review'/exp OR review)) OR 'meta analysis'/exp OR 'meta analysis' |
| #4 | Combine: #1 AND #2 AND #3 |
| **Cochrane Library** | |
| #1 | (endoscopic retrograde cholangiopancreatography OR ERCP):ti,ab,kw |
| #2 | (pancreatitis):ti,ab,kw |
| #3 | (systematic review OR meta-analysis):ti,ab,kw |
| #4 | Combine: #1 AND #2 AND #3 |

September 24th, 2024

**Table S2.** List of excluded studies, with reasons for exclusion after full-text reading.

| **Author** | **Year** | **Title** | **Reason for exclusion** |
| --- | --- | --- | --- |
| Afghani, E. et.al | 2014 | 5-Fr vs. 3-Fr pancreatic stents for the prevention of post-ERCP pancreatitis in high-risk patients: a systematic review and network meta-analysis | Network meta-analysis |
| Ahmad, D. et.al | 2014 | The effect of indomethacin in the prevention of post-endoscopic retrograde cholangiopancreatography pancreatitis: a meta-analysis | Larger meta-analysis in same topic was available |
| Ahmad, Wiqas et.al | 2020 | Pharmacological Prevention of Post-Endoscopic Retrograde Cholangiopancreatography Pancreatitis: Where Do We Stand Now? | Not meta-analysis of randomized controlled trials |
| Akbar, U. A. et.al | 2023 | Preventive Saline Irrigation of the Bile Duct to Reduce the Rate of Residual Common Bile Duct Stones Without Intraductal Ultrasonography: A Systematic Review and Meta-Analysis | Not meta-analysis of randomized controlled trials |
| Akshintala, Venkata S. et.al | 2012 | A Network Meta-Analysis Evaluating the Efficacy of Pharmacologic Prophylaxis for Post-ERCP Pancreatitis | Network meta-analysis |
| Akshintala, V. S. et.al | 2013 | Pancreatic stents and pharmacologic agents for the prevention of post-ERCP pancreatitis in high risk patients: A systematic review and network meta-analysis | Network meta-analysis |
| Akshintala, V. S. et.al | 2013 | Rectal NSAIDs are more effective than pancreatic stents in preventing post-ERCP pancreatitis among high-risk patients: A systematic review and network meta-analysis | Network meta-analysis |
| Akshintala, V. S. et.al | 2023 | Incidence, severity, and mortality of post-ERCP pancreatitis: an updated systematic review and meta-analysis of 145 randomized controlled trials | Qualitative analysis of PEP, not quantitative analysis |
| Akshintala, V. S. et.al | 2014 | 5 Fr pancreatic stents are superior to rectal nsaids and 3 Fr pancreatic stents in preventing post-ERCP pancreatitis in high risk patients: A systematic review and network meta-analysis | Network meta-analysis |
| Aljohani, S. et.al | 2021 | Aggressive Hydration With Ringer's Lactate in the Prevention of Post-ERCP Pancreatitis: A Meta-Analysis | Not meta-analysis of randomized controlled trials |
| Alomari, M. et.al | 2021 | The Incidence of Endoscopic Retrograde Cholangiopancreatography-Related Complications in Patients With Liver Transplant: A Meta-Analysis and Systematic Review | Not meta-analysis of randomized controlled trials |
| Alsakarneh, S. et.al | 2024 | Needle-Knife Fistulotomy Versus Needle-Knife Papillotomy in Difficult Biliary Cannulation: A Systematic Review and Meta-Analysis | Not meta-analysis of randomized controlled trials |
| Alsakarneh, S. et.al | 2024 | Applicability of Child-Turcotte-Pugh Score in Anticipating Post-ERCP Adverse Events in Patients With Cirrhosis: A Systematic Review and Meta-analysis | Not meta-analysis of randomized controlled trials |
| Andriulli, A. et.al | 2003 | Antisecretory vs. antiproteasic drugs in the prevention of post-ERCP pancreatitis: the evidence-based medicine derived from a meta-analysis study | Larger meta-analysis in same topic was available |
| Andriulli, A. et.al | 2007 | Pancreatic duct stents in the prophylaxis of pancreatic damage after endoscopic retrograde cholangiopancreatography: a systematic analysis of benefits and associated risks | Not meta-analysis of randomized controlled trials |
| Andriulli, A. et.al | 2007 | Prophylactic administration of somatostatin or gabexate does not prevent pancreatitis after ERCP: an updated meta-analysis | 9 RCTs were included to compare the effects of somatostatin versus placebo, and 5 RCTs compared gabexate mesilate versus placebo, on the incidence of PEP. However, larger meta-analysis in same topic was available. |
| Andriulli, A. et.al | 2000 | Pharmacologic treatment can prevent pancreatic injury after ERCP: a meta-analysis | Not meta-analysis of randomized controlled trials |
| Aparício, Dpds et.al | 2017 | Endoscopic approach for management of biliary strictures in liver transplant recipients: A systematic review and meta-analysis | Not meta-analysis of randomized controlled trials |
| Aziz, M. et.al | 2020 | Rectal indomethacin with topical epinephrine versus indomethacin alone for preventing Post-ERCP pancreatitis - A systematic review and meta-analysis | Larger meta-analysis in same topic was available |
| Bai, Y. et.al | 2008 | Prophylactic corticosteroids do not prevent post-ERCP pancreatitis: a meta-analysis of randomized controlled trials | Larger meta-analysis in same topic was available |
| Bai, Y. et.al | 2008 | Meta-analysis: allopurinol in the prevention of postendoscopic retrograde cholangiopancreatography pancreatitis | Larger meta-analysis in same topic was available |
| Bai, Y. et.al | 2008 | Prophylactic octreotide administration does not prevent post-endoscopic retrograde cholangiopancreatography pancreatitis: a meta-analysis of randomized controlled trials | Larger meta-analysis in same topic was available |
| Bai, Y. et.al | 2009 | Glyceryl trinitrate for prevention of pancreatitis after endoscopic retrograde cholangiopancreatography: a meta-analysis of randomized, double-blind, placebo-controlled trials | Larger meta-analysis in same topic was available |
| Bang, U. C. et.al | 2009 | Meta-analysis: Nitroglycerin for prevention of post-ERCP pancreatitis | Larger meta-analysis in same topic was available |
| Brand, M. et.al | 2010 | Antibiotic prophylaxis for patients undergoing elective endoscopic retrograde cholangiopancreatography | Larger meta-analysis in same topic was available |
| Cai, Y. L. et.al | 2024 | Endotherapy for the Proximal Migration of Pancreatic Stents: A Systematic Review | Not meta-analysis of randomized controlled trials |
| Cennamo, V. et.al | 2009 | Can a wire-guided cannulation technique increase bile duct cannulation rate and prevent post-ERCP pancreatitis?: A meta-analysis of randomized controlled trials | Larger meta-analysis in same topic was available |
| Cennamo, V. et.al | 2010 | Can early precut implementation reduce endoscopic retrograde cholangiopancreatography-related complication risk? Meta-analysis of randomized controlled trials | Larger meta-analysis in same topic was available |
| Chen, B. et.al | 2010 | A meta-analysis for the effect of prophylactic GTN on the incidence of post-ERCP pancreatitis and on the successful rate of cannulation of bile ducts | Larger meta-analysis in same topic was available |
| Chen, J. et.al | 2018 | Assessing Quality of Precut Sphincterotomy in Patients With Difficult Biliary Access: An Updated Meta-analysis of Randomized Controlled Trials | Larger meta-analysis in same topic was available |
| Chen, J. J. et.al | 2014 | Risk factors for post-ERCP pancreatitis: a systematic review of clinical trials with a large sample size in the past 10 years | Not meta-analysis of randomized controlled trials |
| Chen, S. et.al | 2010 | Role of ulinastatin in preventing post-endoscopic retrograde cholangiopancreatography pancreatitis: the Emperor's New Clothes or Aladdin's Magic Lamp? | Larger meta-analysis in same topic was available. In addition, the control group in 2 randomized clinical trials was gabexate, which did not meet our inclusion criteria. |
| Cheung, J. et.al | 2009 | Guidewire versus conventional contrast cannulation of the common bile duct for the prevention of post-ERCP pancreatitis: a systematic review and meta-analysis | Larger meta-analysis in same topic was available |
| Choi, J. H. et.al | 2023 | Combinatorial Effect of Prophylactic Interventions for Post-ERCP Pancreatitis among Patients with Risk Factors: A Network Meta-Analysis | Network meta-analysis |
| Choi, Jin Ho et.al | 2021 | Comparative effectiveness of prophylactic interventions for post-ERCP pancreatitis among patients with risk factors: A network meta-analysis | Network meta-analysis |
| Choudhary, A. et.al | 2011 | Pancreatic stents for prophylaxis against post-ERCP pancreatitis: a meta-analysis and systematic review | Data insufficient for reanalysis |
| Chuang, T. W. et.al | 2023 | Bile Duct Stone Size May Influence the Efficacy of Endoscopic Sphincterotomy With or Without Large-Balloon Dilation: A Meta-Analysis | Qualitative analysis of PEP, not quantitative analysis |
| Cui, P. J. et.al | 2014 | Biliary stenting with or without sphincterotomy for malignant biliary obstruction: a meta-analysis | Larger meta-analysis in same topic was available |
| Dai, H. F. et.al | 2009 | Role of nonsteroidal anti-inflammatory drugs in the prevention of post-ERCP pancreatitis: a meta-analysis | Larger meta-analysis in same topic was available |
| Dasari, Chandra S. et.al | 2020 | No Added Benefit of Papillary Spraying of Epinephrine over Rectal Indomethacin for Prevention of Post-ERCP Pancreatitis -A Systematic Review and Meta-Analysis of Randomized Controlled Trials | Larger meta-analysis in same topic was available |
| Day, L. W. et.al | 2014 | Adverse events in older patients undergoing ERCP: a systematic review and meta-analysis | Not meta-analysis of randomized controlled trials |
| de Moura, E. T. et.al | 2016 | Guide wire-a sisted cannulation versus conventional contrast to prevent pancreatitis. A systematic review and meta-analysis based on randomized control trials | Larger meta-analysis in same topic was available |
| Devani, K. et.al | 2020 | Pancreatic stent is the best tool to prevent post-ERCP pancreatitis in high-risk patients: Is the result from recent network meta-analysis valid? | Network meta-analysis |
| Dhaliwal, A. et.al | 2021 | Choice of sedation in endoscopic retrograde cholangiopancreatography: Is monitored anesthesia care as safe as general anesthesia? a systematic review and meta-analysis | Not meta-analysis of randomized controlled trials |
| Dhaliwal, A. S. et.al | 2022 | A comparison of balloon-versus stent-based approach for dominant strictures in primary sclerosing cholangitis: a meta-analysis | Not meta-analysis of randomized controlled trials |
| Ding, X. et.al | 2012 | Nonsteroidal anti-inflammatory drugs for prevention of post-ERCP pancreatitis: a meta-analysis | Larger meta-analysis in same topic was available |
| Ding, X. et.al | 2015 | Risk factors for post-ERCP pancreatitis: A systematic review and meta-analysis | Not meta-analysis of randomized controlled trials |
| Elmunzer, B. J. et.al | 2008 | A meta-analysis of rectal NSAIDs in the prevention of post-ERCP pancreatitis | Larger meta-analysis in same topic was available |
| Endo, Y. et.al | 2024 | Comparison Between Plastic and Metallic Biliary Stent Placement for Preoperative Patients with Pancreatic Head Cancer: A Systematic Review and Meta-Analysis | Not meta-analysis of randomized controlled trials |
| Facciorusso, A. et.al | 2021 | Chronic use of statins and risk of post-endoscopic retrograde cholangiopancreatography pancreatitis: a systematic review and meta-analysis | Not meta-analysis of randomized controlled trials |
| Fan, Z. et.al | 2022 | Endoscopic treatment of benign biliary stricture using different stents: a systematic review and meta-analysis | Not meta-analysis of randomized controlled trials |
| Fang, D. et.al | 2023 | Endoscopic retrograde stent drainage therapies for malignant biliary obstruction: the distal opening of stent location above or across the duodenal papilla? A systematic review and meta-analysis | Not meta-analysis of randomized controlled trials |
| Feng, Y. et.al | 2017 | Prophylactic rectal indomethacin may be ineffective for preventing post-endoscopic retrograde cholangiopancreatography pancreatitis in general patients: A meta-analysis | Data insufficient for reanalysis |
| Feng, Y. et.al | 2012 | Comparison of endoscopic papillary large balloon dilation and endoscopic sphincterotomy for retrieval of choledocholithiasis: a meta-analysis of randomized controlled trials | Data insufficient for reanalysis |
| Fu, W. et.al | 2021 | What Would Be the Appropriate Number of Clinical ERCP Cases for Trainees to Acquire Basic Competence? A Systematic Review and Meta-Analysis | Not meta-analysis of randomized controlled trials |
| Gammaro Baldavira Ferreira, Marina Tucci et.al | 2021 | Stent versus Balloon Dilation for the Treatment of Dominant Strictures in Primary Sclerosing Cholangitis: A Systematic Review and Meta-Analysis | Not meta-analysis of randomized controlled trials |
| Garewal, D. et.al | 2012 | Sedative techniques for endoscopic retrograde cholangiopancreatography | Qualitative analysis of PEP, not quantitative analysis |
| Garg, R. et.al | 2018 | Pre-endoscopic retrograde cholangiopancreatography (ERCP) administration of rectal indomethacin in unselected patients to reduce post-ERCP pancreatitis: A systematic review and meta-analysis | Data insufficient for reanalysis |
| Giri, S. et.al | 2022 | Covered Self-Expanding Metal Stents Versus Multiple Plastic Stents for Benign Biliary Strictures: An Updated Meta-Analysis of Randomized Controlled Trials | Qualitative analysis of PEP, not quantitative analysis |
| Giri, S. et.al | 2023 | Risk of post-ERCP pancreatitis is increased in patients with end-stage renal disease - a meta-analysis | Not meta-analysis of randomized controlled trials |
| Gong, B. et.al | 2010 | Does precut technique improve selective bile duct cannulation or increase post-ERCP pancreatitis rate? A meta-analysis of randomized controlled trials | Larger meta-analysis in same topic was available |
| Gooshe, M. et.al | 2015 | Antioxidant therapy in acute, chronic and post-endoscopic retrograde cholangiopancreatography pancreatitis: An updated systematic review and meta-analysis | Larger meta-analysis in same topic was available |
| Gu, W. J. et.al | 2013 | Antioxidant supplementation for the prevention of post-endoscopic retrograde cholangiopancreatography pancreatitis: a meta-analysis of randomized controlled trials | Larger meta-analysis in same topic was available |
| Guzmán-Calderón, E. et.al | 2021 | Transpancreatic precut papillotomy versus double-guidewire technique in difficult biliary cannulation: a systematic review and meta-analysis | This article includes only the results of the comparison between the double-guidewire technique and transpancreatic precut papillotomy. |
| He, X. et.al | 2018 | Rectal Indomethacin Is Protective against Pancreatitis after Endoscopic Retrograde Cholangiopancreatography: Systematic Review and Meta-Analysis | Data insufficient for reanalysis |
| Horváth, I. L. et.al | 2024 | Nafamostat Reduces the Incidence of post-ERCP Pancreatitis: A Systematic Review and Meta-Analysis of Randomized Controlled Trials | Data insufficient for reanalysis |
| Hu, J. et.al | 2016 | Role of Somatostatin in Preventing Post-endoscopic Retrograde Cholangiopancreatography (ERCP) Pancreatitis: An Update Meta-analysis | Larger meta-analysis in same topic was available |
| Inamdar, S. et.al | 2017 | Rectal indomethacin is protective against post-ERCP pancreatitis in high-risk patients but not average-risk patients: a systematic review and meta-analysis | Larger meta-analysis in same topic was available |
| Iqbal, U. et.al | 2022 | Safety and Efficacy of Endoscopic Retrograde Cholangiopancreatography in Nonagenarians: A Systematic Review and Meta-Analysis | Not meta-analysis of randomized controlled trials |
| Jayaraj, M. et.al | 2019 | Periampullary Diverticula and ERCP Outcomes: A Systematic Review and Meta-Analysis | Not meta-analysis of randomized controlled trials |
| Jin, P. P. et.al | 2014 | Endoscopic papillary large balloon dilation vs endoscopic sphincterotomy for retrieval of common bile duct stones: a meta-analysis | Data insufficient for reanalysis |
| Kamal, Faisal et.al | 2022 | Metal versus plastic stents in the management of benign biliary strictures: systematic review and meta-analysis of randomized controlled trials | Qualitative analysis of PEP, not quantitative analysis |
| Keswani, R. N. et.al | 2017 | Association Between Endoscopist and Center Endoscopic Retrograde Cholangiopancreatography Volume With Procedure Success and Adverse Outcomes: A Systematic Review and Meta-analysis | Not meta-analysis of randomized controlled trials |
| Kotha, R. et.al | 2024 | Efficacy of Diclofenac and Indomethacin for Prevention of Post-Endoscopic Cholangiopancreatography (ERCP) Pancreatitis: A Systematic Review | Qualitative analysis of PEP, not quantitative analysis |
| Kovács, N. et.al | 2023 | Suprapapillary Biliary Stents Have Longer Patency Times than Transpapillary Stents—A Systematic Review and Meta-Analysis | Not meta-analysis of randomized controlled trials |
| Kubiliun, N. M. et.al | 2015 | Evaluation of Pharmacologic Prevention of Pancreatitis After Endoscopic Retrograde Cholangiopancreatography: A Systematic Review | Qualitative analysis of PEP, not quantitative analysis |
| Landi, F. et.al | 2018 | Endoscopic treatment of anastomotic biliary stricture after adult deceased donor liver transplantation with multiple plastic stents versus self-expandable metal stents: a systematic review and meta-analysis | Not meta-analysis of randomized controlled trials |
| Li, L. et.al | 2017 | Nonsteroidal anti-inflammatory drugs reduce the incidence of post-endoscopic retrograde cholangiopancreatography pancreatitis: a meta-analysis | Not meta-analysis of randomized controlled trials |
| Li, S. et.al | 2012 | Low-dose heparin in the prevention of post endoscopic retrograde cholangiopancreatography pancreatitis: a systematic review and meta-analysis | Not meta-analysis of randomized controlled trials |
| Li, X. et.al | 2014 | Effectiveness of nonsteroidal anti-inﬂammatory drugs in prevention of post-ERCP pancreatitis: a meta-analysis | Larger meta-analysis in same topic was available |
| Liu, L. et.al | 2019 | Nonsteroidal Anti-inflammatory Drugs for Endoscopic Retrograde Cholangiopancreatography Postoperative Pancreatitis Prevention: a Systematic Review and Meta-analysis | Data insufficient for reanalysis |
| Liu, Y. et.al | 2012 | Endoscopic papillary balloon dilatation versus endoscopic sphincterotomy in the treatment for choledocholithiasis: a meta-analysis | Data insufficient for reanalysis |
| Liu, Y. et.al | 2013 | Endoscopic sphincterotomy plus balloon dilation versus endoscopic sphincterotomy for choledocholithiasis: A meta-analysis | Larger meta-analysis in same topic was available |
| Loganathan, P. et.al | 2024 | Trainee Involvement and ERCP Complications: A Systematic Review and Meta-Analysis | Not meta-analysis of randomized controlled trials |
| Lopez-Cossio, Jorge A. et.al | 2024 | The efficacy and safety of endoscopic sphincterotomy in patients with Sphincter of Oddi dysfunction: a systematic review and meta-analysis | Qualitative analysis of PEP, not quantitative analysis |
| Lyu, Y. et.al | 2021 | Comparison of the Efficacy and Safety of Three Endoscopic Methods to Manage Large Common Bile Duct Stones: A Systematic Review and Network Meta-Analysis | Network meta-analysis |
| Lyu, Y. et.al | 2019 | Comparative Efficacy of 9 Major Drugs for Postendoscopic Retrograde Cholangiopancreatography Pancreatitis: A Network Meta-Analysis | Network meta-analysis |
| Mangiavillano, B. et.al | 2019 | Endoscopic biliary self-expandable metallic stent in malignant biliary obstruction with or without sphincterotomy: systematic review and meta-analysis | Not meta-analysis of randomized controlled trials |
| Márta, K. et.al | 2019 | Combination of intravenous hydration and indomethacin suppository is the most effective therapy for the prevention of post-ercp pancreatiti s-a network meta-analysis | Network meta-analysis |
| Masci, E. et.al | 2003 | Risk factors for pancreatitis following endoscopic retrograde cholangiopancreatography: a meta-analysis | Not meta-analysis of randomized controlled trials |
| Mashiana, H. S. et.al | 2018 | Endoscopic retrograde cholangiopancreatography in cirrhosis - a systematic review and meta-analysis focused on adverse events | Not meta-analysis of randomized controlled trials |
| Mashiana, H. S. et.al | 2018 | Comparison of outcomes for supine vs. prone position ERCP: a systematic review and meta-analysis | Not meta-analysis of randomized controlled trials |
| Mazaki, T. et.al | 2010 | Prophylactic pancreatic stent placement and post-ERCP pancreatitis: a systematic review and meta-analysis | Data insufficient for reanalysis |
| Mc Geehan, G. et.al | 2023 | Prophylactic cholecystectomy offers best outcomes following ERCP clearance of common bile duct stones: a meta-analysis | Not meta-analysis of randomized controlled trials |
| Menon, Shyam et.al | 2020 | The role of intrasphincteric botulinum toxin injection in the management of functional biliary pain: a systematic review and meta-analysis | Not meta-analysis of randomized controlled trials |
| Mohseni Salehi Monfared, S. S. et.al | 2009 | Antioxidant therapy in the management of acute, chronic and post-ERCP pancreatitis: a systematic review | Only data on allopurinol and larger meta-analysis in same topic was available |
| Moss, A. C. et.al | 2007 | Do the benefits of metal stents justify the costs? A systematic review and meta-analysis of trials comparing endoscopic stents for malignant biliary obstruction | Qualitative analysis of PEP, not quantitative analysis |
| Mu, P. et.al | 2020 | Does periampullary diverticulum affect ERCP cannulation and post-procedure complications? an up-to-date meta-analysis | Not meta-analysis of randomized controlled trials |
| Mumtaz, K. et.al | 2007 | Endoscopic retrograde cholangiopancreaticography with or without stenting in patients with pancreaticobiliary malignancy, prior to surgery | Qualitative analysis of PEP, not quantitative analysis |
| Natt, N. et.al | 2022 | ERCP-Related Adverse Events in Primary Sclerosing Cholangitis: A Systematic Review and Meta-Analysis | Not meta-analysis of randomized controlled trials |
| Navaneethan, U. et.al | 2014 | Early precut sphincterotomy and the risk of endoscopic retrograde cholangiopancreatography related complications: An updated meta-analysis | This article shares the same literature and conclusions as the included meta-analysis. |
| Njei, B. et.al | 2020 | Comparative effectiveness of pharmacologic and endoscopic interventions for prevention of post-ERCP pancreatitis: a network meta-analysis | Network meta-analysis |
| Obaitan, I. et.al | 2024 | Comparative Risks of Post-ERCP Adverse Events in Patients with Asymptomatic and Symptomatic Choledocholithiasis: A Systematic Review and Meta-Analysis | Not meta-analysis of randomized controlled trials |
| Omata, F. et.al | 2010 | Meta-analysis: somatostatin or its long-acting analogue, octreotide, for prophylaxis against post-ERCP pancreatitis | Data insufficient for reanalysis |
| Park, Tae Young et.al | 2022 | Rectal NSAIDs-based combination modalities are superior to single modalities for prevention of post-endoscopic retrograde cholangiopancreatog-raphy pancreatitis: a network meta-analysis | Network meta-analysis |
| Park, T. Y. et.al | 2023 | Aggressive hydration for preventing post-endoscopic retrograde cholangiopancreatography pancreatitis: trial sequential analysis | Data insufficient for reanalysis |
| Patai, Á et.al | 2017 | Indomethacin and diclofenac in the prevention of post-ERCP pancreatitis: a systematic review and meta-analysis of prospective controlled trials | Not meta-analysis of randomized controlled trials |
| Patel, A. et.al | 2024 | The Influence of Symptomatic Status on Post-endoscopic Retrograde Cholangiopancreatography (ERCP) Complications in Choledocholithiasis: A Systematic Review and Meta-Analysis | Not meta-analysis of randomized controlled trials |
| Pécsi, D. et.al | 2017 | Transpancreatic sphincterotomy has a higher cannulation success rate than needle-knife precut papillotomy - a meta-analysis | Not meta-analysis of randomized controlled trials |
| Pécsi, D. et.al | 2019 | Transpancreatic Sphincterotomy Is Effective and Safe in Expert Hands on the Short Term | Not meta-analysis of randomized controlled trials |
| Puig, I. et.al | 2014 | How and when should NSAIDs be used for preventing post-ERCP pancreatitis? A systematic review and meta-analysis | Larger meta-analysis in same topic was available |
| Qin, W. Y. et.al | 2020 | Covered and uncovered self-expandable metallic stents in the treatment of malignant biliary obstruction | Data insufficient for reanalysis |
| Qin, X. et.al | 2015 | Prophylactic effect of somatostatin in preventing Post-ERCP pancreatitis: an updated meta-analysis | Larger meta-analysis in same topic was available |
| Radadiya, D. et.al | 2022 | Efficacy of Combining Aggressive Hydration With Rectal Indomethacin in Preventing Post-ERCP Pancreatitis: A Systematic Review and Network Meta-Analysis | Network meta-analysis |
| Radadiya, D. et.al | 2019 | Peri-Procedural Aggressive Hydration for Post Endoscopic Retrograde Cholangiopancreatography (ERCP) Pancreatitis Prophylaxsis: Meta-analysis of Randomized Controlled Trials | Larger meta-analysis in same topic was available |
| Ramai, D. et.al | 2024 | Cannulation rates and technical performance evaluation of commericially available single-use duodenoscopes for endoscopic retrograde cholangiopancreatography: A systematic review and meta-analysis | Not meta-analysis of randomized controlled trials |
| Rojas-Victoria, E. J. et.al | 2024 | Effectiveness of the pharmacological therapy to prevent post ERCP acute pancreatitis: a network meta-analysis | Network meta-analysis |
| Rudin, D. et.al | 2007 | Somatostatin and gabexate for post-endoscopic retrograde cholangiopancreatography pancreatitis prevention: meta-analysis of randomized placebo-controlled trials | 6 RCTs were included to compare the effects of somatostatin versus placebo, and 3 RCTs compared gabexate mesilate versus placebo, on the incidence of PEP. However, larger meta-analysis in same topic was available. |
| Rustagi, T. et.al | 2015 | Factors Affecting the Efficacy of Nonsteroidal Anti-inflammatory Drugs in Preventing Post-Endoscopic Retrograde Cholangiopancreatography Pancreatitis: A Systematic Review and Meta-analysis | Larger meta-analysis in same topic was available |
| Sajid, M. S. et.al | 2015 | Systematic review and meta-analysis on the prophylactic role of non-steroidal anti-inflammatory drugs to prevent post-endoscopic retrograde cholangiopancreatography pancreatitis | Larger meta-analysis in same topic was available |
| Serrano, J. P. R. et.al | 2019 | Nonsteroidal anti-inflammatory drugs versus placebo for post-endoscopic retrograde cholangiopancreatography pancreatitis: a systematic review and meta-analysis | Larger meta-analysis in same topic was available |
| Seta, T. et.al | 2011 | Protease inhibitors for preventing complications associated with ERCP: an updated meta-analysis | Data insufficient for reanalysis |
| Sethi, S. et.al | 2014 | A meta-analysis on the role of rectal diclofenac and indomethacin in the prevention of post-endoscopic retrograde cholangiopancreatography pancreatitis | Larger meta-analysis in same topic was available |
| Shao, L. M. et.al | 2009 | Can wire-guided cannulation reduce the risk of post-endoscopic retrograde cholangiopancreatography pancreatitis? A meta-analysis of randomized controlled trials | Larger meta-analysis in same topic was available |
| Shao, L. M. et.al | 2010 | Nitroglycerin in the prevention of post-ERCP pancreatitis: a meta-analysis | Larger meta-analysis in same topic was available |
| Shen, C. et.al | 2017 | Rectal NSAIDs in the prevention of post-endoscopic retrograde cholangiopancreatography pancreatitis in unselected patients: Systematic review and meta-analysis | Larger meta-analysis in same topic was available |
| Shi, H. et.al | 2013 | Carbon dioxide insufflation during endoscopic retrograde cholangiopancreatography: a review and meta-analysis | Qualitative analysis of PEP, not quantitative analysis |
| Shi, N. et.al | 2015 | Rectal indomethacin for the prevention of post-ERCP pancreatitis: A meta-analysis of randomized controlled trials | Larger meta-analysis in same topic was available |
| Shi, Q. Q. et.al | 2022 | Rectal nonsteroidal anti-inflammatory drugs, glyceryl trinitrate, or combinations for prophylaxis of post-endoscopic retrograde cholangiopancreatography pancreatitis: A network meta-analysis | Network meta-analysis |
| Shi, Q. Q. et.al | 2014 | Placement of prophylactic pancreatic stents to prevent post-endoscopic retrograde cholangiopancreatography pancreatitis in high-risk patients: a meta-analysis | Larger meta-analysis in same topic was available |
| Shou-Xin, Y. et.al | 2020 | Rectal nonsteroidal anti-inflammatory drugs and pancreatic stents in preventing post-endoscopic retrograde cholangiopancreatography pancreatitis in high-risk patients: A network meta-analysis | Network meta-analysis |
| Siddappa, P. K. et.al | 2021 | Endoscopic pancreatic duct stenting for pain palliation in selected pancreatic cancer patients: a systematic review and meta-analysis | Not meta-analysis of randomized controlled trials |
| Siiki, A. et.al | 2014 | Covered self-expanding metal stents may be preferable to plastic stents in the treatment of chronic pancreatitis-related biliary strictures: a systematic review comparing 2 methods of stent therapy in benign biliary strictures | Not meta-analysis of randomized controlled trials |
| Siiki, Antti et.al | 2018 | A systematic review of biodegradable biliary stents: promising biocompatibility without stent removal | Not meta-analysis of randomized controlled trials |
| Singh, P. et.al | 2004 | Does prophylactic pancreatic stent placement reduce the risk of post-ERCP acute pancreatitis? A meta-analysis of controlled trials | Not meta-analysis of randomized controlled trials |
| Smeets, X. J. et.al | 2016 | Systematic review: periprocedural hydration in the prevention of post-ERCP pancreatitis | Qualitative analysis of PEP, not quantitative analysis |
| Sofi, A. A. et.al | 2021 | Comparison of clinical outcomes of multiple plastic stents and covered metal stent in refractory pancreatic ductal strictures in chronic pancreatitis- a systematic review and meta-analysis | Not meta-analysis of randomized controlled trials |
| Sofi, A. A. et.al | 2016 | Does endoscopic sphincterotomy reduce the risk of post-endoscopic retrograde cholangiopancreatography pancreatitis after biliary stenting? A systematic review and meta-analysis | Larger meta-analysis in same topic was available |
| Søreide, J. A. et.al | 2019 | Endoscopic retrograde cholangiopancreatography (ERCP): lessons learned from population-based national registries: a systematic review | Qualitative analysis of PEP, not quantitative analysis |
| Sun, H. L. et.al | 2014 | Rectal NSAIDs for the prevention of post-ERCP pancreatitis: a meta-analysis of randomized controlled trials | Larger meta-analysis in same topic was available |
| Sundaralingam, P. et.al | 2015 | Early Precut Sphincterotomy Does Not Increase Risk During Endoscopic Retrograde Cholangiopancreatography in Patients With Difficult Biliary Access: A Meta-analysis of Randomized Controlled Trials | Larger meta-analysis in same topic was available |
| Tarar, Z. I. et.al | 2023 | Safety of endoscopic retrograde cholangiopancreatography (ERCP) in cirrhosis compared to non-cirrhosis and effect of Child-Pugh score on post-ERCP complications: a systematic review and meta-analysis | Not meta-analysis of randomized controlled trials |
| Tari, E. et.al | 2024 | Morphology of the papilla can predict procedural safety and efficacy of ERCP-a systematic review and meta-analysis | Not meta-analysis of randomized controlled trials |
| Teles de Campos, S. et.al | 2023 | Impact of center and endoscopist ERCP volume on ERCP outcomes: a systematic review and meta-analysis | Not meta-analysis of randomized controlled trials |
| Tse, F. et.al | 2016 | Pancreatic duct guidewire placement for biliary cannulation for the prevention of post-endoscopic retrograde cholangiopancreatography (ERCP) pancreatitis | This article was sourced from the Cochrane Database of Systematic Reviews, and the formal study conclusions were published in journals and included in the subsequent analysis. |
| Tse, F. et.al | 2012 | Guidewire-assisted cannulation of the common bile duct for the prevention of post-endoscopic retrograde cholangiopancreatography (ERCP) pancreatitis | Larger meta-analysis in same topic was available |
| Tse, F. et.al | 2013 | Guide wire-assisted cannulation for the prevention of post-ERCP pancreatitis: a systematic review and meta-analysis | Larger meta-analysis in same topic was available |
| Vadalà di Prampero, S. F. et.al | 2016 | Endoscopic and pharmacological treatment for prophylaxis against postendoscopic retrograde cholangiopancreatography pancreatitis: a meta-analysis and systematic review | This article presents a comprehensive meta-analysis on endoscopic and pharmacological treatments for preventing PEP. However, there are updated meta-analyses available for each specific topic. |
| van Geenen, E. J. et.al | 2013 | Lack of consensus on the role of endoscopic retrograde cholangiography in acute biliary pancreatitis in published meta-analyses and guidelines: a systematic review | Qualitative analysis of PEP, not quantitative analysis |
| Vanella, G. et.al | 2024 | Fully covered versus partially covered self-expandable metal stents for palliation of distal malignant biliary obstruction: a systematic review and meta-analysis | Not meta-analysis of randomized controlled trials |
| Xiang, Zhi-yong et.al | 2023 | The Incidence of Long-Term Cholangitis after Endoscopic Papillary Balloon Dilation Versus Endoscopic Sphincterotomy: a Meta-analysis | Larger meta-analysis in same topic was available |
| Yaghoobi, M. et.al | 2018 | Rectal Indomethacin Prevents Moderate to Severe Post-ERCP Pancreatitis and Death and Should Be Used Before the Procedure: A Meta-Analysis of Aggregate Subgroup Data | Larger meta-analysis in same topic was available |
| Yaghoobi, M. et.al | 2013 | Meta-analysis: rectal indomethacin for the prevention of post-ERCP pancreatitis | Larger meta-analysis in same topic was available |
| Yang, C. et.al | 2017 | Rectal nonsteroidal anti-inflammatory drugs administration is effective for the prevention of post-ERCP pancreatitis: An updated meta-analysis of randomized controlled trials | Larger meta-analysis in same topic was available |
| Yang, H. et.al | 2023 | Meta-analysis on clinical outcomes of suprapapillary versus transpapillary stent insertion in malignant biliary obstruction | Not meta-analysis of randomized controlled trials |
| Yang, J. et.al | 2020 | Rectal Nonsteroidal Anti-Inflammatory Drugs for Endoscopic Retrograde Cholangiopancreatography Postoperative Pancreatitis Prevention: A Network Meta-Analysis | Network meta-analysis |
| Yang, X. M. et.al | 2013 | Endoscopic sphincterotomy plus large-balloon dilation vs endoscopic sphincterotomy for choledocholithiasis: a meta-analysis | Larger meta-analysis in same topic was available |
| Yu, G. et.al | 2015 | Nafamostat mesilate for prevention of post-ERCP pancreatitis: a meta-analysis of prospective, randomized, controlled trials | Larger meta-analysis in same topic was available |
| Yu, L. M. et.al | 2018 | Use of NSAIDs via the Rectal Route for the Prevention of Pancreatitis after ERCP in All-Risk Patients: An Updated Meta-Analysis | Larger meta-analysis in same topic was available |
| Yu, S. et.al | 2021 | Rectal indomethacin and diclofenac are equally efficient in preventing pancreatitis following endoscopic retrograde cholangiopancreatography in average-risk patients | Larger meta-analysis in same topic was available |
| Zeng, C. et.al | 2022 | Prevention of pancreatitis after stent implantation for distal malignant biliary strictures: systematic review and meta-analysis | Not meta-analysis of randomized controlled trials |
| Zhang, W. Y. et.al | 2017 | Efficacy and safety of carbon dioxide insufflation versus air insufflation for endoscopic retrograde cholangiopancreatography: A meta-analysis update | Qualitative analysis of PEP, not quantitative analysis |
| Zhang, Z. F. et.al | 2017 | Aggressive Hydration With Lactated Ringer Solution in Prevention of Postendoscopic Retrograde Cholangiopancreatography Pancreatitis: A Meta-analysis of Randomized Controlled Trials | Larger meta-analysis in same topic was available |
| Zhang, Z. F. et.al | 2010 | Preventive effect of ulinastatin and gabexate mesylate on post-endoscopic retrograde cholangiopancreatography pancreatitis | Data insufficient for reanalysis |
| Zheng, M. et.al | 2008 | Meta-analysis of prophylactic allopurinol use in post-endoscopic retrograde cholangiopancreatography pancreatitis | Larger meta-analysis in same topic was available |
| Zheng, M. et.al | 2007 | Gabexate in the prophylaxis of post-ERCP pancreatitis: a meta-analysis of randomized controlled trials | Larger meta-analysis in same topic was available |
| Zheng, M. H. et.al | 2009 | Effectiveness and tolerability of NSAIDs in the prophylaxis of pancreatitis after endoscopic retrograde cholangiopancreatography: A systematic review and meta-analysis | Data insufficient for reanalysis |
| Zhou, B. et.al | 2023 | Risk factors for post-retrograde cholangiopancreatography pancreatitis in patients with common bile duct stones: A meta-analysis | Not meta-analysis of randomized controlled trials |
| Zhu, K. et.al | 2017 | Prophylactic ulinastatin administration for preventing post-endoscopic retrograde cholangiopancreatography pancreatitis: A meta-analysis | Not meta-analysis of randomized controlled trials |
| Zorrón Pu, L. et.al | 2015 | Endoscopic stenting for inoperable malignant biliary obstruction: A systematic review and meta-analysis | Qualitative analysis of PEP, not quantitative analysis |

**Table S3.** Corrected Covered Area Assessment of 64 associations.

**(1)**

| **Topic:** Effect of allopurinol (***vs.*** placebo) on the incidence of PEP. | | | |
| --- | --- | --- | --- |
| **Systematic Reviews** | **Bai Y, 2008** | **Zheng M, 2008** | **Cao WL, 2014** |
| **Primary Studies** |  |  |  |
| Abbasinazari, 2011 |  |  | • |
| Martinez-Torres, 2009 |  |  | • |
| Romagnuolo, 2008 | • |  | • |
| Katsinelos, 2005 | • | • | • |
| Mosler, 2005 | • | • | • |
| Budzynska, 2001 | • | • | • |
| Badra, 2000 |  | • |  |
| Budzynska, 1997 |  | • |  |
| Clemens, 1991 |  | • |  |
| **Total** | **4** | **6** | **6** |
| **Grand Total (N)** | **16** | | |
| **Rows (r)** | **9** | | |
| **Columns (c)** | **3** | | |
| **CCA** | **38.89%** | | |
| **Degree of overlap** | **Very High overlap** | | |

**(2)**

| **Topic:** Effect of allopurinol (***vs.*** placebo) on the incidence of mild PEP. | | |
| --- | --- | --- |
| **Systematic Reviews** | **Bai Y, 2008** | **Cao WL, 2014** |
| **Primary Studies** |  |  |
| Abbasinazari, 2011 |  | • |
| Martinez-Torres, 2009 |  | • |
| Romagnuolo, 2008 | • | • |
| Katsinelos, 2005 | • | • |
| Mosler, 2005 | • | • |
| Budzynska, 2001 | • | • |
| **Total** | **4** | **6** |
| **Grand Total (N)** | **10** | |
| **Rows (r)** | **6** | |
| **Columns (c)** | **2** | |
| **CCA** | **66.67%** | |
| **Degree of overlap** | **Very High overlap** | |

**(3)**

| **Topic:** Effect of allopurinol (***vs.*** placebo) on the incidence of moderate PEP. | | |
| --- | --- | --- |
| **Systematic Reviews** | **Bai Y, 2008** | **Cao WL, 2014** |
| **Primary Studies** |  |  |
| Abbasinazari, 2011 |  | • |
| Romagnuolo, 2008 | • | • |
| Katsinelos, 2005 | • | • |
| Mosler, 2005 | • | • |
| Budzynska, 2001 | • | • |
| **Total** | **4** | **5** |
| **Grand Total (N)** | **9** | |
| **Rows (r)** | **5** | |
| **Columns (c)** | **2** | |
| **CCA** | **80.00%** | |
| **Degree of overlap** | **Very High overlap** | |

**(4)**

| **Topic:** Effect of allopurinol (***vs.*** placebo) on the incidence of severe PEP. | | | |
| --- | --- | --- | --- |
| **Systematic Reviews** | **Bai Y, 2008** | **Zheng M, 2008** | **Cao WL, 2014** |
| **Primary Studies** |  |  |  |
| Romagnuolo, 2008 | • |  | • |
| Katsinelos, 2005 | • | • | • |
| Mosler, 2005 | • | • | • |
| Budzynska, 2001 | • | • | • |
| Budzynska, 1997 |  | • |  |
| **Total** | **4** | **4** | **4** |
| **Grand Total (N)** | **12** | | |
| **Rows (r)** | **5** | | |
| **Columns (c)** | **3** | | |
| **CCA** | **70.00%** | | |
| **Degree of overlap** | **Very High overlap** | | |

**(5)**

| **Topic:** Effect of epinephrine + NSAIDs (***vs.*** NSAIDs) on the incidence of mild PEP. | | | |
| --- | --- | --- | --- |
| **Systematic Reviews** | **Aziz M, 2020** | **Dasari CS, 2020** | **Chen Z, 2024** |
| **Primary Studies** |  |  |  |
| Hatami, 2018 | • | • | • |
| Kamal, 2019 |  | • |  |
| Luo, 2019 | • | • | • |
| Dar, 2021 |  |  | • |
| Romano-Munive, 2021 |  |  | • |
| **Total** | **2** | **3** | **4** |
| **Grand Total (N)** | **9** | | |
| **Rows (r)** | **5** | | |
| **Columns (c)** | **3** | | |
| **CCA** | **40.00%** | | |
| **Degree of overlap** | **Very High overlap** | | |

**(6)**

| **Topic:** Effect of epinephrine + NSAIDs (***vs.*** NSAIDs) on the incidence of moderate to severe PEP. | | | |
| --- | --- | --- | --- |
| **Systematic Reviews** | **Aziz M, 2020** | **Dasari CS, 2020** | **Chen Z, 2024** |
| **Primary Studies** |  |  |  |
| Hatami, 2018 | • | • | • |
| Kamal, 2019 | • | • |  |
| Luo, 2019 | • | • | • |
| Dar, 2021 |  |  | • |
| Romano-Munive, 2021 |  |  | • |
| **Total** | **3** | **3** | **4** |
| **Grand Total (N)** | **10** | | |
| **Rows (r)** | **5** | | |
| **Columns (c)** | **3** | | |
| **CCA** | **50.00%** | | |
| **Degree of overlap** | **Very High overlap** | | |

**(7)**

| **Topic:** Effect of gabexate mesilate (***vs.*** placebo/no treatment) on the incidence of PEP. | | | | | | |
| --- | --- | --- | --- | --- | --- | --- |
| **Systematic Reviews** | **Rudin D, 2007** | **Andriulli A, 2007** | **Zheng MH, 2007** | **Zheng MH, 2008** | **Chiu YJ, 2021** | **Yuhara H, 2014** |
| **Primary Studies** |  |  |  |  |  |  |
| Meng, 2007 |  |  |  |  | • |  |
| Cavallini, 1996 | • | • | • | • | • | • |
| Manes, 2007 |  |  |  | • | • | • |
| Lee, 2007 |  |  |  |  | • |  |
| Yokohata, 2003 |  |  |  |  | • | • |
| Benvenuti, 2006 |  | • |  | • | • |  |
| Min, 2008 |  |  |  |  | • |  |
| Xiong, 2006 |  | • | • | • | • | • |
| Masci, 2003 |  |  |  |  | • |  |
| Andriulli, 2004 | • | • | • | • | • | • |
| Andriulli, 2002 | • | • | • | • | • | • |
| Shimizu, 1979 |  |  |  |  | • |  |
| Yoo, 2012 |  |  |  |  |  | • |
| **Total** | **3** | **5** | **4** | **6** | **12** | **7** |
| **Grand Total (N)** | **37** | | | | | |
| **Rows (r)** | **13** | | | | | |
| **Columns (c)** | **6** | | | | | |
| **CCA** | **36.92%** | | | | | |
| **Degree of overlap** | **Very High overlap** | | | | | |

**(8)**

| **Topic:** Effect of glyceryl trinitrate (***vs.*** placebo/physiological saline/vitamin C/no intervention) on the incidence of PEP. | | | | | |
| --- | --- | --- | --- | --- | --- |
| **Systematic Reviews** | **Bang UC, 2009** | **Bai Y, 2009** | **Chen B, 2010** | **Shao LM, 2010** | **Ding J, 2013** |
| **Primary Studies** |  |  |  |  |  |
| Beauchant M, 2008 | • | • | • | • | • |
| Bhatia V, 2011 |  |  |  |  | • |
| Chen XW, 2012 |  |  |  |  | • |
| Hao JY, 2009 |  | • | • |  | • |
| Kaffes AJ, 2006 | • | • | • | • | • |
| Moretó M, 2003 | • | • | • | • | • |
| Nashaat E, 2010 |  |  |  |  | • |
| Nøjgaard C, 2009 | • | • | • |  | • |
| Sudhindran, 2001 | • | • | • | • | • |
| Talwar A, 2005 |  | • |  |  | • |
| Wehrmann, 2001 |  | • | • |  | • |
| **Total** | **5** | **8** | **7** | **4** | **11** |
| **Grand Total (N)** | **35** | | | | |
| **Rows (r)** | **11** | | | | |
| **Columns (c)** | **5** | | | | |
| **CCA** | **54.55%** | | | | |
| **Degree of overlap** | **Very High overlap** | | | | |

**(9)**

| **Topic:** Effect of glyceryl trinitrate (***vs.*** placebo/physiological saline/vitamin C/no intervention) on the incidence of moderate to severe PEP. | | |
| --- | --- | --- |
| **Systematic Reviews** | **Shao LM, 2010** | **Ding J, 2013** |
| **Primary Studies** |  |  |
| Moretó M, 2003 | • | • |
| Kaffes AJ, 2006 | • | • |
| Beauchant M, 2008 | • | • |
| Nøjgaard C, 2009 |  | • |
| Chen XW, 2012 |  | • |
| **Total** | **3** | **5** |
| **Grand Total (N)** | **8** | |
| **Rows (r)** | **5** | |
| **Columns (c)** | **2** | |
| **CCA** | **60.00%** | |
| **Degree of overlap** | **Very High overlap** | |

**(10)**

| **Topic:** Effect of epinephrine + NSAIDs (***vs.*** NSAIDs) on the incidence of PEP. | | | | | |
| --- | --- | --- | --- | --- | --- |
| **Systematic Reviews** | **Aziz M, 2020** | **Dasari CS, 2020** | **Iqbal U, 2020** | **El Hage Chehade N, 2023** | **Chen Z, 2024** |
| **Primary Studies** |  |  |  |  |  |
| Hatami, 2018 | • | • | • | • | • |
| Kamal, 2019 | • | • | • | • | • |
| Luo, 2019 | • | • | • | • | • |
| Alipour, 2020 |  |  |  |  | • |
| Dar, 2021 |  |  |  | • | • |
| Romano-Munive, 2021 |  |  |  | • | • |
| **Total** | **3** | **3** | **3** | **5** | **6** |
| **Grand Total (N)** | **20** | | | | |
| **Rows (r)** | **6** | | | | |
| **Columns (c)** | **5** | | | | |
| **CCA** | **58.33%** | | | | |
| **Degree of overlap** | **Very High overlap** | | | | |

**(11)**

| **Topic:** Effect of NSAIDs (***vs.*** no NSAIDs) on the incidence of moderate to severe PEP. | | | | | | | | | | | |
| --- | --- | --- | --- | --- | --- | --- | --- | --- | --- | --- | --- |
| **Systematic Reviews** | **Elmunzer BJ, 2008** | **Ding X, 2012** | **Li X, 2014** | **Puig I, 2014** | **Sethi S, 2014** | **Sun HL, 2014** | **Shen C, 2017** | **Yang C, 2017** | **Yu LM, 2018** | **Hou YC, 2017** | **El Kurdi B, 2024** |
| **Primary Studies** |  |  |  |  |  |  |  |  |  |  |  |
| Abu-Safieh, 2014 |  |  |  |  |  |  |  |  |  |  | • |
| Andrade-Dávila, 20150 |  |  |  |  |  |  |  | • | • | • | • |
| Cheon, 2007 |  | • | • | • |  |  |  |  |  |  | • |
| Dobronte, 2012 |  |  |  |  |  |  |  |  |  | • | • |
| Dobronte, 2014 |  |  |  |  |  |  | • | • | • | • | • |
| Elmunzer, 2012 |  | • | • | • | • | • |  | • | • | • | • |
| Hauser, 2016 |  |  |  |  |  |  |  |  |  |  | • |
| Ishiwatari, 2016 |  |  |  |  |  |  |  |  |  |  | • |
| Katoh, 2019 |  |  |  |  |  |  |  |  |  |  | • |
| Katsinelos, 2011 |  |  |  |  | • |  |  |  |  |  | • |
| Levenick, 2016 |  |  |  |  |  |  | • | • | • | • | • |
| Lua, 2015 |  |  |  |  |  |  |  |  |  | • | • |
| Mansour, 2016 |  |  |  |  |  |  |  | • |  |  | • |
| Murray, 2003 | • | • | • | • | • | • |  | • | • | • | • |
| Otuska, 2012 |  | • | • | • | • | • | • | • | • | • | • |
| Park, 2014 |  |  |  |  |  |  |  |  |  |  | • |
| Patai, 2015 |  |  |  |  |  |  | • | • | • | • | • |
| Sotoudehmanesh, 2007 | • | • | • | • | • | • | • | • | • | • | • |
| Ucar, 2016 |  |  |  |  |  |  | • |  |  | • | • |
| Elmi, 2010 |  | • |  |  |  |  |  |  |  |  |  |
| Alabd, 2013 |  |  |  |  |  |  |  |  |  | • |  |
| Luo, 2016 |  |  |  |  |  |  |  |  |  | • |  |
| **Total** | **2** | **6** | **5** | **5** | **5** | **4** | **6** | **9** | **8** | **13** | **19** |
| **Grand Total (N)** | **82** | | | | | | | | | | |
| **Rows (r)** | **22** | | | | | | | | | | |
| **Columns (c)** | **11** | | | | | | | | | | |
| **CCA** | **27.27%** | | | | | | | | | | |
| **Degree of overlap** | **Very High overlap** | | | | | | | | | | |

**(12)**

| **Topic:** Effect of indomethacin (***vs.*** no NSAIDs) on the incidence of PEP. | | | | | | | | | | | | | | | | | |
| --- | --- | --- | --- | --- | --- | --- | --- | --- | --- | --- | --- | --- | --- | --- | --- | --- | --- |
| **Systematic Reviews** | **Yaghoobi M,**  **2013** | **Ahmad D,**  **2014** | **Sethi S,**  **2014** | **Rustagi T,**  **2015** | **Sajid MS,**  **2015** | **Shi N,**  **2015** | **Inamdar S,**  **2017** | **Shen C,**  **2017** | **Yang C,**  **2017** | **Yu LM,**  **2018** | **Yaghoobi M,**  **2018** | **Serrano JPR,**  **2019** | **Yu S,**  **2021** | **Serrano JPR,**  **2020** | **El Kurdi B,**  **2024** | **Lyu Y,**  **2018** | **Wan J,**  **2017** |
| **Primary Studies** |  |  |  |  |  |  |  |  |  |  |  |  |  |  |  |  |  |
| Montano Loza, 2007 | • | • | • | • | • | • | • | • | • | • | • | • | • |  | • | • | • |
| Sotoudehmanesh, 2007 | • | • | • | • | • | • | • | • | • | • | • |  | • | • | • | • | • |
| Katsinelos, 2011 |  |  |  |  |  |  |  |  |  |  |  |  |  |  | • |  |  |
| Dobronte, 2012 | • | • |  | • | • |  | • | • | • |  | • |  | • |  | • | • |  |
| Elmunzer, 2012 | • | • | • | • | • | • | • |  | • | • | • | • |  | • | • | • | • |
| Dobronte, 2014 |  |  |  |  | • |  | • | • | • | • | • | • | • | • | • | • | • |
| Andrade-Davila, 2015 |  |  |  |  |  |  | • |  | • | • | • | • |  | • | • | • | • |
| Patai, 2015 |  |  |  |  |  |  | • | • | • | • | • | • | • | • | • | • | • |
| Hosseini, 2016 |  |  |  |  |  |  |  |  |  |  |  |  | • |  | • | • |  |
| Levenick, 2016 |  |  |  |  |  |  | • | • | • | • | • | • | • | • | • | • | • |
| Elmi, 2010 |  |  |  | • |  |  |  |  |  |  |  |  |  |  |  |  |  |
| Montano Loza, 2006 |  |  |  |  | • |  |  |  |  |  |  |  |  |  |  |  |  |
| Mousalreza, 2016 |  |  |  |  |  |  |  |  |  |  |  | • |  | • |  |  |  |
| Li, 2019 |  |  |  |  |  |  |  |  |  |  |  |  |  | • |  |  |  |
| **Total** | **4** | **4** | **3** | **5** | **6** | **3** | **8** | **6** | **8** | **7** | **8** | **7** | **7** | **8** | **10** | **9** | **7** |
| **Grand Total (N)** | **110** | | | | | | | | | | | | | | | | |
| **Rows (r)** | **14** | | | | | | | | | | | | | | | | |
| **Columns (c)** | **17** | | | | | | | | | | | | | | | | |
| **CCA** | **42.86%** | | | | | | | | | | | | | | | | |
| **Degree of overlap** | **Very High overlap** | | | | | | | | | | | | | | | | |

**(13)**

| **Topic:** Effect of indomethacin (***vs.*** no NSAIDs) on the incidence of moderate to severe PEP. | | | | | |
| --- | --- | --- | --- | --- | --- |
| **Systematic Reviews** | **Ahmad D, 2014** | **Shi N, 2015** | **Yaghoobi M, 2018** | **El Kurdi B, 2024** | **Wan J, 2017** |
| **Primary Studies** |  |  |  |  |  |
| Sotoudehmanesh, 2007 | • | • | • | • | • |
| Katsinelos, 2011 |  |  |  | • |  |
| Dobronte, 2012 | • |  | • | • |  |
| Elmunzer, 2012 | • | • | • | • | • |
| Dobronte, 2014 |  |  | • | • | • |
| Andrade-Davila, 2015 |  |  | • | • | • |
| Patai, 2015 |  |  | • | • | • |
| Levenick, 2016 |  |  | • | • | • |
| **Total** | **3** | **2** | **7** | **8** | **6** |
| **Grand Total (N)** | **26** | | | | |
| **Rows (r)** | **8** | | | | |
| **Columns (c)** | **5** | | | | |
| **CCA** | **56.25%** | | | | |
| **Degree of overlap** | **Very High overlap** | | | | |

**(14)**

| **Topic:** Effect of antioxidants (***vs.*** placebo/no intervention) on the incidence of PEP. | | | | |
| --- | --- | --- | --- | --- |
| **Systematic Reviews** | **Mohseni Salehi Monfared SS, 2009** | **Gu WJ, 2013** | **Gooshe M, 2015** | **Fuentes-Orozco C, 2015** |
| **Primary Studies** |  |  |  |  |
| Wollschläger, 1999 |  | • |  | • |
| Budzyúska, 2001 | • | • | • | • |
| Lavy, 2004 |  | • | • | • |
| Katsinelos, 2005 | • | • | • | • |
| Katsinelos, 2005 |  | • | • | • |
| Mosler, 2005 | • | • | • | • |
| Milewski, 2006 |  | • | • | • |
| Kapetanos, 2007 |  | • | • | • |
| Romagnuolo, 2008 | • | • | • | • |
| Martinez-Torres, 2009 |  | • | • | • |
| Abbasinazari, 2011 |  | • | • | • |
| Alavi Nejad, 2013 |  |  |  | • |
| Kapetanos, 2009 |  |  | • |  |
| **Total** | **4** | **11** | **11** | **12** |
| **Grand Total (N)** | **38** | | | |
| **Rows (r)** | **13** | | | |
| **Columns (c)** | **4** | | | |
| **CCA** | **64.10%** | | | |
| **Degree of overlap** | **Very High overlap** | | | |

**(15)**

| **Topic:** Effect of antioxidants (***vs.*** placebo/no intervention) on the incidence of mild PEP. | | |
| --- | --- | --- |
| **Systematic Reviews** | **Gu WJ, 2013** | **Fuentes-Orozco C, 2015** |
| **Primary Studies** |  |  |
| Budzyúska, 2001 | • | • |
| Lavy, 2004 | • | • |
| Katsinelos, 2005 | • | • |
| Katsinelos, 2005 | • | • |
| Mosler, 2005 | • | • |
| Kapetanos, 2007 | • | • |
| Romagnuolo, 2008 | • | • |
| Martinez-Torres, 2009 |  | • |
| Abbasinazari, 2011 | • | • |
| **Total** | **8** | **9** |
| **Grand Total (N)** | **17** | |
| **Rows (r)** | **9** | |
| **Columns (c)** | **2** | |
| **CCA** | **88.89%** | |
| **Degree of overlap** | **Very High overlap** | |

**(16)**

| **Topic:** Effect of antioxidants (***vs.*** placebo/no intervention) on the incidence of moderate PEP. | | |
| --- | --- | --- |
| **Systematic Reviews** | **Gu WJ, 2013** | **Fuentes-Orozco C, 2015** |
| **Primary Studies** |  |  |
| Budzyúska, 2001 | • | • |
| Lavy, 2004 | • | • |
| Katsinelos, 2005 | • | • |
| Katsinelos, 2005 | • | • |
| Mosler, 2005 | • | • |
| Kapetanos, 2007 | • | • |
| Romagnuolo, 2008 | • | • |
| Abbasinazari, 2011 | • | • |
| **Total** | **8** | **8** |
| **Grand Total (N)** | **16** | |
| **Rows (r)** | **8** | |
| **Columns (c)** | **2** | |
| **CCA** | **100.00%** | |
| **Degree of overlap** | **Very High overlap** | |

**(17)**

| **Topic:** Effect of antioxidants (***vs.*** placebo/no intervention) on the incidence of severe PEP. | | |
| --- | --- | --- |
| **Systematic Reviews** | **Gu WJ, 2013** | **Fuentes-Orozco C, 2015** |
| **Primary Studies** |  |  |
| Budzyúska, 2001 | • | • |
| Lavy, 2004 | • | • |
| Katsinelos, 2005 | • | • |
| Mosler, 2005 | • | • |
| Kapetanos, 2007 | • | • |
| Romagnuolo, 2008 | • | • |
| **Total** | **6** | **6** |
| **Grand Total (N)** | **12** | |
| **Rows (r)** | **6** | |
| **Columns (c)** | **2** | |
| **CCA** | **100.00%** | |
| **Degree of overlap** | **Very High overlap** | |

**(18)**

| **Topic:** Effect of NSAIDs (***vs.*** placebo/glycerin/no treatment) on the incidence of mild PEP. | | | | | | |
| --- | --- | --- | --- | --- | --- | --- |
| **Systematic Reviews** | **Li X, 2014** | **Sun HL, 2013** | **Yu LM, 2018** | **Serrano JPR, 2019** | **Serrano JPR, 2020** | **Hou YC, 2017** |
| **Primary Studies** |  |  |  |  |  |  |
| Alabd, 2013 |  |  |  |  |  | • |
| Andrade-Davila, 2015 |  |  | • | • | • | • |
| Dobronte, 2012 |  |  |  |  |  | • |
| Dobronte, 2014 |  |  | • | • | • | • |
| Elmunzer, 2012 | • | • | • | • | • | • |
| Hosseini, 2016 |  |  |  |  |  | • |
| Levenick, 2016 |  |  | • | • | • | • |
| Lua, 2015 |  |  |  | • | • | • |
| Luo, 2016 |  |  |  |  |  | • |
| Murray, 2003 | • | • | • |  |  | • |
| Otsuka, 2012 | • | • | • | • | • | • |
| Patai, 2015 |  |  | • | • | • | • |
| Sotoudehmanesh, 2007 | • | • | • |  |  | • |
| Ucar, 2016 |  |  |  | • | • | • |
| Montano, 2007 | • |  | • |  |  |  |
| Cheon, 2007 | • |  |  | • | • |  |
| Hauser, 2016 |  |  |  | • | • |  |
| Ishiwatari, 2016 |  |  |  | • | • |  |
| Leerhoy, 2016 |  |  |  | • | • |  |
| Mansour, 2016 |  |  |  | • | • |  |
| Park, 2014 |  |  |  | • | • |  |
| **Total** | **6** | **4** | **9** | **14** | **14** | **14** |
| **Grand Total (N)** | **61** | | | | | |
| **Rows (r)** | **21** | | | | | |
| **Columns (c)** | **6** | | | | | |
| **CCA** | **38.10%** | | | | | |
| **Degree of overlap** | **Very High overlap** | | | | | |

**(19)**

| **Topic:** Effect of epinephrine (***vs.*** placebo/no treatment) on the incidence of PEP. | | |
| --- | --- | --- |
| **Systematic Reviews** | **Iqbal U, 2020** | **Chen Z, 2024** |
| **Primary Studies** |  |  |
| Chaitanya, 2016 | • |  |
| Matsushita, 2009 | • | • |
| Xu, 2011 | • | • |
| **Total** | **3** | **2** |
| **Grand Total (N)** | **5** | |
| **Rows (r)** | **3** | |
| **Columns (c)** | **2** | |
| **CCA** | **66.67%** | |
| **Degree of overlap** | **Very High overlap** | |

**(20)**

| **Topic:** Effect of NSAIDs (***vs.*** placebo) on the incidence of PEP in high-risk patients. | | | | | |
| --- | --- | --- | --- | --- | --- |
| **Systematic Reviews** | **Sethi S, 2014** | **Inamdar S, 2017** | **Yang C, 2017** | **Yu LM, 2018** | **Lyu Y, 2018** |
| **Primary Studies** |  |  |  |  |  |
| Andrade-Dávila, 2015 |  | • | • | • | • |
| Cheon, 2007 |  |  |  |  | • |
| Elmunze, 2012 | • | • | • | • | • |
| Khoshbaten, 2008 | • |  | • | • | • |
| Lua, 2015 |  |  |  |  | • |
| Murray, 2003 | • |  | • | • | • |
| Patai, 2015 |  | • |  |  |  |
| **Total** | **3** | **3** | **4** | **4** | **6** |
| **Grand Total (N)** | **20** | | | | |
| **Rows (r)** | **7** | | | | |
| **Columns (c)** | **5** | | | | |
| **CCA** | **46.43%** | | | | |
| **Degree of overlap** | **Very High overlap** | | | | |

**(21)**

| **Topic:** Effect of antibiotics (***vs.*** non-antibiotics) on the incidence of PEP. | | |
| --- | --- | --- |
| **Systematic Reviews** | **Brand M, 2010** | **Merchan MFS, 2022** |
| **Primary Studies** |  |  |
| Brandes JW, 1981 | • | • |
| Niederau C, 1994 | • | • |
| Räty S, 2001 | • | • |
| Spickan J, 2002 | • | • |
| **Total** | **4** | **4** |
| **Grand Total (N)** | **8** | |
| **Rows (r)** | **4** | |
| **Columns (c)** | **2** | |
| **CCA** | **100.00%** | |
| **Degree of overlap** | **Very High overlap** | |

**(22)**

| **Topic:** Effect of nafamostat mesylate (***vs.*** placebo/no treatment) on the incidence of PEP. | | | | |
| --- | --- | --- | --- | --- |
| **Systematic Reviews** | **Yu G, 2015** | **Narumi K, 2023** | **Xie Y, 2023** | **Yuhara H, 2014** |
| **Primary Studies** |  |  |  |  |
| Matsumoto T, 2021 |  | • | • |  |
| Ohuchida J, 2015 | • | • | • |  |
| Park'Y, 2014 |  | • | • |  |
| Kwon YH, 2012 | • | • | • |  |
| Park KT, 2011 | • | • | • | • |
| Yoo kS, 2011 | • | • | • | • |
| Moon SW, 2010 | • | • | • |  |
| Choi CW, 2009 | • | • | • | • |
| Yamashita, 2010 | • |  |  | • |
| Ohuchida, 2011 |  |  |  | • |
| **Total** | **7** | **8** | **8** | **5** |
| **Grand Total (N)** | **28** | | | |
| **Rows (r)** | **10** | | | |
| **Columns (c)** | **4** | | | |
| **CCA** | **60.00%** | | | |
| **Degree of overlap** | **Very High overlap** | | | |

**(23)**

| **Topic:** Effect of nafamostat mesylate (***vs.*** placebo/no treatment) on the incidence of mild PEP. | | | |
| --- | --- | --- | --- |
| **Systematic Reviews** | **Yu G, 2015** | **Narumi K, 2023** | **Xie Y, 2023** |
| **Primary Studies** |  |  |  |
| Matsumoto T, 2021 |  | • | • |
| Ohuchida J, 2015 |  | • | • |
| Park JY, 2014 |  | • | • |
| Kwon YH, 2012 | • | • | • |
| Park KT, 2011 | • | • | • |
| Yoo KS, 2011 | • | • | • |
| Choi CW, 2009 | • | • | • |
| **Total** | **4** | **7** | **7** |
| **Grand Total (N)** | **18** | | |
| **Rows (r)** | **7** | | |
| **Columns (c)** | **3** | | |
| **CCA** | **78.57%** | | |
| **Degree of overlap** | **Very High overlap** | | |

**(24)**

| **Topic:** Effect of NSAIDs (***vs.*** placebo SN/placebo lactated Ringer’s solution/ceftazidime/glycerin/glyceryl trinitrate/saline solution/no treatment) on the incidence of PEP. | | | | | | | | | | | | | | | | | | |
| --- | --- | --- | --- | --- | --- | --- | --- | --- | --- | --- | --- | --- | --- | --- | --- | --- | --- | --- |
| **Systematic Reviews** | **Elmunzer BJ, 2008** | **Dai HF,**  **2009** | **Ding X, 2012** | **Li X,**  **2014** | **Puig I,**  **2014** | **Sethi S,**  **2014** | **Sun HL,**  **2013** | **Rustagi T,**  **2015** | **Sajid MS,**  **2015** | **Shen C,**  **2017** | **Yang C,**  **2017** | **Yu LM,**  **2018** | **Serrano JPR,**  **2019** | **Yu S,**  **2021** | **Serrano JPR,**  **2020** | **Hou YC,**  **2017** | **El Kurdi B,**  **2024** | **Lyu Y,**  **2018** |
| **Primary Studies** |  |  |  |  |  |  |  |  |  |  |  |  |  |  |  |  |  |  |
| Andrade, 2015 |  |  |  |  |  |  |  |  |  |  | • | • | • |  | • | • | • | • |
| Bathia, 2011 |  |  | • |  |  |  |  | • |  |  |  |  | • |  | • |  | • | • |
| Cheon, 2007 |  | • | • | • | • |  |  | • | • |  |  |  | • |  | • |  | • | • |
| Dobronte, 2014 |  |  |  |  |  |  |  |  | • | • | • | • | • | • | • | • | • | • |
| Elmunzer, 2012 |  |  | • | • | • | • | • | • | • |  | • | • | • |  | • | • | • | • |
| Hauser, 2016 |  |  |  |  |  |  |  |  |  |  |  |  | • |  | • |  | • |  |
| Ishiwatari, 2016 |  |  |  |  |  |  |  |  |  |  |  |  | • |  | • |  | • |  |
| Kato K, 2017 |  |  |  |  |  |  |  |  |  |  |  |  |  |  | • |  |  | • |
| Kato T, 2019 |  |  |  |  |  |  |  |  |  |  |  |  |  |  | • |  | • |  |
| Koshbaten, 2008 | • | • | • | • | • | • | • | • | • |  | • | • | • |  | • | • |  | • |
| Leerhoy, 2016 |  |  |  |  |  |  |  |  |  |  |  |  | • |  | • |  |  |  |
| Levenick, 2016 |  |  |  |  |  |  |  |  |  | • | • | • | • | • | • | • | • | • |
| Li, 2019 |  |  |  |  |  |  |  |  |  |  |  |  |  |  | • |  |  |  |
| Lua, 2015 |  |  |  |  |  |  |  |  |  |  |  |  | • |  | • | • | • | • |
| Mansour, 2016 |  |  |  |  |  |  |  |  |  |  | • |  | • |  | • |  | • | • |
| Masjerdizadeh, 2017 |  |  |  |  |  |  |  |  |  |  |  |  |  |  | • |  |  |  |
| Montaño, 2007 | • | • | • | • | • | • | • | • | • | • | • | • | • | • | • | • | • | • |
| Mousalreza, 2016 |  |  |  |  |  |  |  |  |  |  |  |  | • |  | • |  |  |  |
| Murray, 2003 | • | • | • | • | • | • | • | • | • |  | • | • | • |  | • | • | • | • |
| Otsuka, 2012 |  |  | • | • | • | • | • | • | • | • | • | • | • |  | • | • | • | • |
| Park, 2014 |  |  |  |  |  |  |  |  | • |  |  |  | • |  | • |  | • | • |
| Patai, 2015 |  |  |  |  |  |  |  |  |  | • | • | • | • | • | • | • | • | • |
| Quadros, 2016 |  |  |  |  |  |  |  |  |  |  |  |  | • |  | • |  |  |  |
| Senol, 2009 |  |  | • | • | • |  |  | • | • |  |  |  | • |  | • |  | • |  |
| Sotoudehmanesh, 2007 | • | • | • | • | • | • | • | • | • | • | • | • |  | • | • | • | • | • |
| Ucar, 2016 |  |  |  |  |  |  |  |  |  | • |  |  | • | • | • | • | • | • |
| Elmi, 2010 |  |  | • |  |  |  |  | • |  |  |  |  |  |  |  |  |  |  |
| Dobronte, 2012 |  |  |  |  | • |  | • | • | • | • | • |  |  | • |  | • | • | • |
| Katsinelos, 2011 |  |  |  |  |  | • |  |  |  |  |  |  |  |  |  |  | • |  |
| Montaño, 2006 |  | • |  |  |  |  |  |  | • |  |  |  |  |  |  |  |  |  |
| Zhao, 2014 |  |  |  |  |  |  |  |  | • |  |  |  |  |  |  |  |  |  |
| Arain, 2013 |  |  |  |  |  |  |  |  |  | • |  |  |  | • |  |  |  |  |
| Shafique MS, 2016 |  |  |  |  |  |  |  |  |  |  |  | • |  | • |  |  |  |  |
| Hosseini, 2016 |  |  |  |  |  |  |  |  |  |  |  |  |  | • |  | • | • | • |
| Alabd, 2013 |  |  |  |  |  |  |  |  |  |  |  |  |  |  |  | • |  |  |
| Luo, 2016 |  |  |  |  |  |  |  |  |  |  |  |  |  |  |  | • |  |  |
| Abu-Safieh, 2014 |  |  |  |  |  |  |  |  |  |  |  |  |  |  |  |  | • | • |
| de Quadros, 2016 |  |  |  |  |  |  |  |  |  |  |  |  |  |  |  |  | • | • |
| **Total** | **4** | **6** | **10** | **8** | **9** | **7** | **7** | **11** | **13** | **9** | **12** | **11** | **21** | **10** | **26** | **16** | **24** | **21** |
| **Grand Total (N)** | **225** | | | | | | | | | | | | | | | | | |
| **Rows (r)** | **38** | | | | | | | | | | | | | | | | | |
| **Columns (c)** | **18** | | | | | | | | | | | | | | | | | |
| **CCA** | **28.95%** | | | | | | | | | | | | | | | | | |
| **Degree of overlap** | **Very High overlap** | | | | | | | | | | | | | | | | | |

**(25)**

| **Topic:** Effect of NSAIDs (***vs.*** placebo SN/placebo lactated Ringer’s solution/ceftazidime/glycerin/glyceryl trinitrate/saline solution/no treatment) on the incidence of moderate PEP. | | |
| --- | --- | --- |
| **Systematic Reviews** | **Serrano JPR, 2019** | **Serrano JPR, 2020** |
| **Primary Studies** |  |  |
| Andrade, 2015 | • | • |
| Cheon, 2007 | • | • |
| Elmunzer, 2012 | • | • |
| Hauser, 2016 | • | • |
| Ishiwatari, 2016 | • | • |
| Leerhoy, 2016 | • | • |
| Mansour, 2016 | • | • |
| Park, 2014 | • | • |
| Patai, 2015 | • | • |
| Quadros, 2016 | • | • |
| Ucar, 2016 | • | • |
| **Total** | **11** | **11** |
| **Grand Total (N)** | **22** | |
| **Rows (r)** | **11** | |
| **Columns (c)** | **2** | |
| **CCA** | **100.00%** | |
| **Degree of overlap** | **Very High overlap** | |

**(26)**

| **Topic:** Effect of NSAIDs (***vs.*** placebo SN/placebo lactated Ringer’s solution/ceftazidime/glycerin/glyceryl trinitrate/saline solution/no treatment) on the incidence of severe PEP. | | |
| --- | --- | --- |
| **Systematic Reviews** | **Serrano JPR, 2019** | **Serrano JPR, 2020** |
| **Primary Studies** |  |  |
| Cheon, 2007 | • | • |
| Dobrönte, 2014 | • | • |
| Elmunzer, 2012 | • | • |
| Hauser, 2016 | • | • |
| Ishiwatari, 2016 | • | • |
| Leerhoy, 2016 | • | • |
| Patai, 2015 | • | • |
| **Total** | **7** | **7** |
| **Grand Total (N)** | **14** | |
| **Rows (r)** | **7** | |
| **Columns (c)** | **2** | |
| **CCA** | **100.00%** | |
| **Degree of overlap** | **Very High overlap** | |

**(27)**

| **Topic:** Effect of diclofenac (***vs.*** placebo SN/placebo lactated Ringer’s solution/ceftazidime/glycerin/glyceryl trinitrate/saline solution /no treatment) on the incidence of PEP. | | | | | | | | | | |
| --- | --- | --- | --- | --- | --- | --- | --- | --- | --- | --- |
| **Systematic Reviews** | **Sethi S,**  **2014** | **Sajid MS, 2015** | **Shen C, 2017** | **Yang C, 2017** | **Yu LM, 2018** | **Serrano JPR, 2019** | **Yu S,**  **2021** | **Serrano JPR, 2020** | **Lyu Y,**  **2018** | **El Kurdi B, 2024** |
| **Primary Studies** |  |  |  |  |  |  |  |  |  |  |
| Cheon, 2007 |  | • |  |  |  | • |  | • | • | • |
| Hauser, 2016 |  |  |  |  |  | • |  | • |  | • |
| Ishiwatari, 2016 |  |  |  |  |  | • |  | • |  | • |
| Kato T, 2019 |  |  |  |  |  |  |  | • |  | • |
| Khoshbaten, 2008 | • | • |  | • | • | • |  | • | • |  |
| Leerhoy, 2016 |  |  |  |  |  | • |  | • |  |  |
| Lua, 2015 |  |  |  |  |  | • |  | • | • | • |
| Masjerdizadeh, 2017 |  |  |  |  |  |  |  | • |  |  |
| Montano, 2007 |  |  |  |  |  |  |  | • |  |  |
| Murray, 2003 | • | • |  | • | • | • |  | • | • | • |
| Otsuka, 2012 | • | • | • | • | • | • |  | • | • | • |
| Park, 2014 |  | • |  |  |  | • |  | • | • | • |
| Senol, 2009 |  | • |  |  |  | • |  | • |  | • |
| Uçar, 2016 |  |  | • |  |  | • | • | • | • | • |
| Katsinelos P, 2011 | • |  |  |  |  |  |  |  |  |  |
| Zhao, 2014 |  | • |  |  |  |  |  |  |  |  |
| Arain, 2013 |  |  | • |  |  |  | • |  |  |  |
| Shafque MS, 2016 |  |  |  |  | • |  | • |  |  |  |
| Abu-Safieh, 2014 |  |  |  |  |  |  |  |  | • | • |
| Katoh, 2019 |  |  |  |  |  |  |  |  |  | • |
| **Total** | **4** | **7** | **3** | **3** | **4** | **11** | **3** | **14** | **8** | **12** |
| **Grand Total (N)** | **69** | | | | | | | | | |
| **Rows (r)** | **20** | | | | | | | | | |
| **Columns (c)** | **10** | | | | | | | | | |
| **CCA** | **27.22%** | | | | | | | | | |
| **Degree of overlap** | **Very High overlap** | | | | | | | | | |

**(28)**

| **Topic:** Effect of indomethacin (***vs.*** placebo) on the incidence of mild PEP. | | |
| --- | --- | --- |
| **Systematic Reviews** | **Ahmad D, 2014** | **Wan J, 2017** |
| **Primary Studies** |  |  |
| Andrade-Dávila, 2015 |  | • |
| Dobronte, 2014 |  | • |
| Elmunzer, 2012 | • | • |
| Levenick, 2016 |  | • |
| Montano Loza, 2007 | • | • |
| Patai, 2015 |  | • |
| Sotoudehmanesh, 2007 | • | • |
| **Total** | **3** | **7** |
| **Grand Total (N)** | **10** | |
| **Rows (r)** | **7** | |
| **Columns (c)** | **2** | |
| **CCA** | **42.86%** | |
| **Degree of overlap** | **Very High overlap** | |

**(29)**

| **Topic:** Effect of indomethacin (***vs.*** placebo) on the incidence of PEP in high-risk patients. | | | | |
| --- | --- | --- | --- | --- |
| **Systematic Reviews** | **Inamdar S, 2017** | **Yaghoobi M, 2018** | **Lyu Y, 2018** | **Wan J, 2017** |
| **Primary Studies** |  |  |  |  |
| Andrade-Davila, 2015 | • | • | • | • |
| Elmunzer, 2012 | • | • | • | • |
| Palai, 2015 | • |  |  | • |
| **Total** | **3** | **2** | **2** | **3** |
| **Grand Total (N)** | **10** | | | |
| **Rows (r)** | **3** | | | |
| **Columns (c)** | **4** | | | |
| **CCA** | **77.78%** | | | |
| **Degree of overlap** | **Very High overlap** | | | |

**(30)**

| **Topic:** Effect of somatostatin (***vs.*** placebo/no treatment) on the incidence of PEP. | | | | | | |
| --- | --- | --- | --- | --- | --- | --- |
| **Systematic Reviews** | **Andriulli A, 2003** | **Qin X, 2015** | **Hu J, 2016** | **Wang G, 2018** | **Andriulli A, 2007** | **Rudin D, 2007** |
| **Primary Studies** |  |  |  |  |  |  |
| Andriuli A, 2004 |  | • | • | • | • | • |
| Bai Y, 2015 |  |  | • | • |  |  |
| Chan HH, 2008 |  | • | • | • |  |  |
| Cacepcion-wartin M, 2014 |  |  | • | • |  |  |
| LeeKT, 2008 |  | • | • | • |  |  |
| Poon RT, 1999 | • | • | • | • | • | • |
| Wang ZK, 2013 |  | • | • | • |  |  |
| Zhao LN, 2014 |  |  |  | • |  |  |
| Andriuli A, 2002 | • | • | • | • | • | • |
| Aranitids A, 2004 |  | • | • | • | • | • |
| Bordas JM, 1988 | • | • |  | • |  | • |
| Bordas JM, 1998 | • | • | • | • | • | • |
| Persson B, 1992 | • | • |  | • | • | • |
| Poon RT, 2003 |  | • | • | • | • | • |
| Deschner, 1989 | • |  |  |  |  | • |
| Saari, 1988 | • |  |  |  | • | • |
| Borsch, 1984 | • |  |  |  |  |  |
| Testoni, 1988 | • |  |  |  |  | • |
| Flati, 1988 | • |  |  |  |  |  |
| Guerlud, 1991 | • |  |  |  |  | • |
| Benvenuti, 2006 |  |  |  |  | • |  |
| **Total** | **11** | **11** | **11** | **14** | **9** | **12** |
| **Grand Total (N)** | **68** | | | | | |
| **Rows (r)** | **21** | | | | | |
| **Columns (c)** | **6** | | | | | |
| **CCA** | **44.76%** | | | | | |
| **Degree of overlap** | **Very High overlap** | | | | | |

**(31)**

| **Topic:** Effect of aggressive hydration with Lactated Ringer Solution (***vs.*** standard hydration with Lactated Ringer Solution) on the incidence of PEP. | | | | | |
| --- | --- | --- | --- | --- | --- |
| **Systematic Reviews** | **Zhang ZF, 2017** | **Radadiya D, 2019** | **Wu D, 2017** | **Wu M, 2021** | **Wang RC, 2021** |
| **Primary Studies** |  |  |  |  |  |
| Buxbaum, 2014 | • | • | • | • | • |
| Chuan, 2015 | • | • |  | • | • |
| M Alciva-Leon, 2018 |  | • |  | • |  |
| Park, 2017 |  | • |  | • | • |
| Rosa, 2016 | • | • |  | • | • |
| Shaygan, 2015 | • | • | • | • | • |
| Chang As, 2016 | • | • |  | • | • |
| Choi, 2016 | • | • | • | • | • |
| NCTO2050048, 2016 | • | • |  | • |  |
| Ramin, 2019 |  |  |  | • |  |
| **Total** | **7** | **9** | **3** | **10** | **7** |
| **Grand Total (N)** | **36** | | | | |
| **Rows (r)** | **10** | | | | |
| **Columns (c)** | **5** | | | | |
| **CCA** | **65.00%** | | | | |
| **Degree of overlap** | **Very High overlap** | | | | |

**(32)**

| **Topic:** Effect of nafamostat mesylate (***vs.*** placebo/no treatment) on the incidence of moderate PEP. | | |
| --- | --- | --- |
| **Systematic Reviews** | **Yu G, 2015** | **Xie Y, 2023** |
| **Primary Studies** |  |  |
| Cheol woong Choi, 2009 | • | • |
| Ji Young Park, 2014 |  | • |
| Jiro Ohuchida, 2015 |  | • |
| Kee Tae Park, 2011 | • | • |
| Kyo-Sang Yoo, 2011 | • | • |
| Takaaki Matsumoto, 2021 |  | • |
| Yong Hwan Kwon, 2012 | • | • |
| **Total** | **4** | **7** |
| **Grand Total (N)** | **11** | |
| **Rows (r)** | **7** | |
| **Columns (c)** | **2** | |
| **CCA** | **57.14%** | |
| **Degree of overlap** | **Very High overlap** | |

**(33)**

| **Topic:** Effect of nafamostat mesylate (***vs.*** placebo/no treatment) on the incidence of severe PEP. | | |
| --- | --- | --- |
| **Systematic Reviews** | **Yu G, 2015** | **Xie Y, 2023** |
| **Primary Studies** |  |  |
| Jiro Ohuchida, 2015 |  | • |
| Takaaki Matsumoto, 2021 |  | • |
| Yong Hwan Kwon, 2012 | • | • |
| **Total** | **1** | **3** |
| **Grand Total (N)** | **4** | |
| **Rows (r)** | **3** | |
| **Columns (c)** | **2** | |
| **CCA** | **33.33%** | |
| **Degree of overlap** | **Very High overlap** | |

**(34)**

| **Topic:** Effect of nafamostat mesylate (***vs.*** placebo/no treatment) on the incidence of PEP in high-risk patients. | | | |
| --- | --- | --- | --- |
| **Systematic Reviews** | **Yu G, 2015** | **Narumi K, 2023** | **Xie Y, 2023** |
| **Primary Studies** |  |  |  |
| Cheol Woong Choi, 2009 | • | • | • |
| Ji Young Park, 2014 |  |  | • |
| Jiro Ohuchida, 2015 |  | • | • |
| Kee Tae Park, 2011 | • | • | • |
| Kyo-Sang Yoo, 2011 | • | • | • |
| Takaaki Matsumoto, 2021 |  | • | • |
| **Total** | **3** | **5** | **6** |
| **Grand Total (N)** | **14** | | |
| **Rows (r)** | **6** | | |
| **Columns (c)** | **3** | | |
| **CCA** | **66.67%** | | |
| **Degree of overlap** | **Very High overlap** | | |

**(35)**

| **Topic:** Effect of ulinastatin (***vs.*** placebo) on the incidence of PEP. | | |
| --- | --- | --- |
| **Systematic Reviews** | **Chen S, 2010** | **Yuhara H, 2014** |
| **Primary Studies** |  |  |
| Tsujino, 2005 | • | • |
| Gong, 2004 | • | • |
| Song, 2004 | • | • |
| Chen, 2004 | • | • |
| Yoo, 2008 | • | • |
| Yoo, 2012 |  | • |
| **Total** | **5** | **6** |
| **Grand Total (N)** | **11** | |
| **Rows (r)** | **6** | |
| **Columns (c)** | **2** | |
| **CCA** | **83.33%** | |
| **Degree of overlap** | **Very High overlap** | |

**(36)**

| **Topic:** Effect of octreotide (***vs.*** placebo) on the incidence of PEP. | | |
| --- | --- | --- |
| **Systematic Reviews** | **Bai Y, 2008** | **Zhang Y, 2009** |
| **Primary Studies** |  |  |
| Binmoeller, 1992 | • | • |
| Russo, 1992 | • | • |
| Stemlieb, 1992 | • | • |
| Arcidiacono, 1994 | • | • |
| Armonis, 1994 | • | • |
| Baldazzi, 1994 |  | • |
| Testoni, 1994 | • | • |
| Ng, 1997 | • | • |
| Arvanitidis, 1998 | • | • |
| Manolakopoulos, 2002 | • | • |
| Testoni, 1996 | • | • |
| Duvnjak, 1999 | • | • |
| Badra, 2000 | • | • |
| Testoni, 2001 | • | • |
| Thomopoulos, 2006 | • | • |
| Li ZS, 2007 | • | • |
| **Total** | **15** | **16** |
| **Grand Total (N)** | **31** | |
| **Rows (r)** | **16** | |
| **Columns (c)** | **2** | |
| **CCA** | **93.75%** | |
| **Degree of overlap** | **Very High overlap** | |

**(37)**

| **Topic:** Effect of corticosteroid (***vs.*** placebo) on the incidence of PEP. | | |
| --- | --- | --- |
| **Systematic Reviews** | **Bai Y, 2008** | **Zheng M, 2008** |
| **Primary Studies** |  |  |
| Budzynska, 1997 |  | • |
| Dumot, 1998 | • | • |
| De Palma, 1999 | • | • |
| Budzynska, 2001 | • | • |
| Manolakopoulos, 2002 | • | • |
| Sherman, 2003 | • | • |
| Kwanngern, 2005 | • | • |
| **Total** | **6** | **7** |
| **Grand Total (N)** | **13** | |
| **Rows (r)** | **7** | |
| **Columns (c)** | **2** | |
| **CCA** | **85.71%** | |
| **Degree of overlap** | **Very High overlap** | |

**(38)**

| **Topic:** Effect of gabexate mesilate (***vs.*** placebo/no treatment) on the incidence of severe PEP. | | |
| --- | --- | --- |
| **Systematic Reviews** | **Zheng MH, 2007** | **Zheng MH, 2008** |
| **Primary Studies** |  |  |
| Andriulli, 2002 | • | • |
| Andriuli, 2004 | • | • |
| Benvenuti, 2006 |  | • |
| Manes, 2007 |  | • |
| **Total** | **2** | **4** |
| **Grand Total (N)** | **6** | |
| **Rows (r)** | **4** | |
| **Columns (c)** | **2** | |
| **CCA** | **50.00%** | |
| **Degree of overlap** | **Very High overlap** | |

**(39)**

| **Topic:** Effect of endocut (***vs.*** pure cut) on the incidence of PEP. | | |
| --- | --- | --- |
| **Systematic Reviews** | **Funari MP, 2020** | **Bicudo de Oliveira L, 2024** |
| **Primary Studies** |  |  |
| Ellahi, 2000 | • | • |
| Kida, 2004 | • | • |
| Norton, 2015 | • | • |
| Funari, 2023 |  | • |
| **Total** | **3** | **4** |
| **Grand Total (N)** | **7** | |
| **Rows (r)** | **4** | |
| **Columns (c)** | **2** | |
| **CCA** | **75.00%** | |
| **Degree of overlap** | **Very High overlap** | |

**(40)**

| **Topic:** Effect of early precut sphincterotomy (***vs.*** persistent cannulation attempts) on the incidence of PEP. | | | | | | | |
| --- | --- | --- | --- | --- | --- | --- | --- |
| **Systematic Reviews** | **Navaneethan U,**  **2014** | **Cennamo V,**  **2010** | **Gong B,**  **2010** | **Sundaralingam P,**  **2015** | **Chen J,**  **2018** | **Tang Z,**  **2018** | **Choudhary A,**  **2014** |
| **Primary Studies** |  |  |  |  |  |  |  |
| Cennamo, 2009 | • | • | • | • | • | • | • |
| de Weerth, 2006 | • | • | • |  |  |  | • |
| Khatibian, 2008 | • | • | • |  |  |  | • |
| Manes, 2009 | • | • | • | • | • | • | • |
| Swan, 2013 | • |  |  | • | • | • | • |
| Tang, 2005 | • | • | • | • | • | • | • |
| Zhou, 2006 | • | • | • | • | • | • | • |
| Mariani, 2016 |  |  |  |  | • | • |  |
| Zagalsky, 2016 |  |  |  |  |  | • |  |
| **Total** | **7** | **6** | **6** | **5** | **6** | **7** | **7** |
| **Grand Total (N)** | **44** | | | | | | |
| **Rows (r)** | **9** | | | | | | |
| **Columns (c)** | **7** | | | | | | |
| **CCA** | **64.81%** | | | | | | |
| **Degree of overlap** | **Very High overlap** | | | | | | |

**(41)**

| **Topic:** Effect of pancreatic stent placement (***vs.*** no pancreatic stent placement) on the incidence of mild PEP in high-risk patients. | | |
| --- | --- | --- |
| **Systematic Reviews** | **Shi QQ, 2014** | **da Cruz Portela, 2019** |
| **Primary Studies** |  |  |
| Cha, 2012 |  | • |
| Fazel A, 2003 | • | • |
| Kawaguchi, 2012 | • | • |
| Lee TH, 2012 | • | • |
| Smithline.A, 1993 |  | • |
| Sofuni, 2007 | • | • |
| Sofuni, 2011 | • | • |
| Tarnasky, 1998 | • | • |
| Tsuchiya, 2007 | • | • |
| Ito, 2010 | • |  |
| **Total** | **8** | **9** |
| **Grand Total (N)** | **17** | |
| **Rows (r)** | **10** | |
| **Columns (c)** | **2** | |
| **CCA** | **70.00%** | |
| **Degree of overlap** | **Very High overlap** | |

**(42)**

| **Topic:** Effect of pancreatic stent placement (***vs.*** no pancreatic stent placement) on the incidence of moderate PEP in high-risk patients. | | |
| --- | --- | --- |
| **Systematic Reviews** | **Shi QQ, 2014** | **da Cruz Portela, 2019** |
| **Primary Studies** |  |  |
| Cha, 2012 |  | • |
| Faze.A, 2003 | • | • |
| LeeTH, 2012 | • | • |
| Smmithline.A, 1993 |  | • |
| Sofuni, 2007 | • | • |
| Sofuni, 2011 | • | • |
| Tarnasky, 1998 | • | • |
| Tsuchiya, 2007 | • | • |
| **Total** | **6** | **8** |
| **Grand Total (N)** | **14** | |
| **Rows (r)** | **8** | |
| **Columns (c)** | **2** | |
| **CCA** | **75.00%** | |
| **Degree of overlap** | **Very High overlap** | |

**(43)**

| **Topic:** Effect of pancreatic stent placement (***vs.*** no pancreatic stent placement) on the incidence of severe PEP in high-risk patients. | | |
| --- | --- | --- |
| **Systematic Reviews** | **Shi QQ, 2014** | **da Cruz Portela, 2019** |
| **Primary Studies** |  |  |
| Cha, 2012 |  | • |
| Faze.A, 2003 | • | • |
| LeeTH, 2012 | • | • |
| Smmithline.A, 1993 |  | • |
| Sofuni, 2011 | • | • |
| Tarnasky, 1998 |  | • |
| Tsuchiya, 2007 | • | • |
| **Total** | **4** | **7** |
| **Grand Total (N)** | **11** | |
| **Rows (r)** | **7** | |
| **Columns (c)** | **2** | |
| **CCA** | **57.14%** | |
| **Degree of overlap** | **Very High overlap** | |

**(44)**

| **Topic:** Effect of endoscopic sphincterotomy associated with balloon dilation (***vs.*** endoscopic sphincterotomy) on the incidence of PEP. | | | |
| --- | --- | --- | --- |
| **Systematic Reviews** | **Liu Y, 2013** | **Yang XM, 2013** | **de Clemente Junior CC, 2018** |
| **Primary Studies** |  |  |  |
| Guo, 2015 |  |  | • |
| Heo, 2007 | • | • | • |
| Hong, 2009 |  | • | • |
| Karsenti, 2017 |  |  | • |
| Kim TH, 2009 | • | • | • |
| Li, 2013 |  |  | • |
| Qian, 2013 |  |  | • |
| Takeshi, 2015 |  |  | • |
| Teoh, 2013 |  | • | • |
| Stefanidis, 2011 |  | • |  |
| Liu, 2008 | • |  |  |
| **Total** | **3** | **5** | **9** |
| **Grand Total (N)** | **17** | | |
| **Rows (r)** | **11** | | |
| **Columns (c)** | **3** | | |
| **CCA** | **27.27%** | | |
| **Degree of overlap** | **Very High overlap** | | |

**(45)**

| **Topic:** Effect of pancreatic stent placement (***vs.*** no pancreatic stent placement) on the incidence of mild PEP. | | |
| --- | --- | --- |
| **Systematic Reviews** | **Zhu G, 2015** | **Fan JH, 2015** |
| **Primary Studies** |  |  |
| Cha, 2012 | • | • |
| Fazel, 2003 | • | • |
| Harewood, 2005 |  | • |
| Ito, 2010 | • | • |
| Karaguchi, 2012 | • | • |
| Lee, 2012 | • | • |
| Patel, 1999 |  | • |
| Sherman, 1996 |  | • |
| Smithline, 1993 | • | • |
| Sofuni, 2007 | • | • |
| Sofuni, 2011 | • | • |
| Thanasky, 1998 |  | • |
| Tsuchiya, 2007 | • | • |
| Conlgliaro, 2012 | • |  |
| **Total** | **10** | **13** |
| **Grand Total (N)** | **23** | |
| **Rows (r)** | **14** | |
| **Columns (c)** | **2** | |
| **CCA** | **64.29%** | |
| **Degree of overlap** | **Very High overlap** | |

**(46)**

| **Topic:** Effect of pancreatic stent placement (***vs.*** no pancreatic stent placement) on the incidence of severe PEP. | | | |
| --- | --- | --- | --- |
| **Systematic Reviews** | **Zhu G, 2015** | **Mazaki T, 2014** | **Fan JH, 2015** |
| **Primary Studies** |  |  |  |
| Cha, 2012 | • | • | • |
| Fazel, 2003 | • | • | • |
| Lee, 2012 | • | • | • |
| Patel, 1999 |  | • | • |
| Sherman, 1996 |  | • | • |
| Smithline, 1993 | • | • | • |
| Sofuni, 2011 | • | • | • |
| Tsuchiya, 2007 | • | • | • |
| Conlgliaro, 2012 | • |  |  |
| **Total** | **7** | **8** | **8** |
| **Grand Total (N)** | **23** | | |
| **Rows (r)** | **9** | | |
| **Columns (c)** | **3** | | |
| **CCA** | **77.78%** | | |
| **Degree of overlap** | **Very High overlap** | | |

**(47)**

| **Topic:** Effect of endocut (***vs.*** blend) on the incidence of PEP. | | |
| --- | --- | --- |
| **Systematic Reviews** | **Funari MP, 2020** | **Li DF, 2019** |
| **Primary Studies** |  |  |
| A.Kohler, 1998 | • | • |
| Hirotada Akiho, 2006 |  | • |
| Yoshiki Tanaka, 2015 | • | • |
| **Total** | **2** | **3** |
| **Grand Total (N)** | **5** | |
| **Rows (r)** | **3** | |
| **Columns (c)** | **2** | |
| **CCA** | **66.67%** | |
| **Degree of overlap** | **Very High overlap** | |

**(48)**

| **Topic:** Effect of endoscopic sphincterotomy (***vs.*** Non-endoscopic sphincterotomy) on the incidence of PEP. | | | |
| --- | --- | --- | --- |
| **Systematic Reviews** | **Cui PJ, 2014** | **Sofi AA, 2016** | **Martins OC, 2024** |
| **Primary Studies** |  |  |  |
| Giorgio, 2004 | • | • | • |
| Hayashi, 2015 |  | • | • |
| Kato, 2022 |  |  | • |
| Mavrogiannis, 2006 |  | • | • |
| Zhang, 2014 |  |  | • |
| Zhou, 2012 | • | • | • |
| **Total** | **2** | **4** | **6** |
| **Grand Total (N)** | **12** | | |
| **Rows (r)** | **6** | | |
| **Columns (c)** | **3** | | |
| **CCA** | **50.00%** | | |
| **Degree of overlap** | **Very High overlap** | | |

**(49)**

| **Topic:** Effect of pancreatic stent placement (***vs.*** no pancreatic stent placement) on the incidence of PEP in high-risk patients. | | | | |
| --- | --- | --- | --- | --- |
| **Systematic Reviews** | **Shi QQ, 2014** | **Vadalà di Prampero SF, 2016** | **da Cruz Portela, 2019** | **Mazaki T, 2014** |
| **Primary Studies** |  |  |  |  |
| Smithline, 1993 |  | • | • | • |
| Sherman, 1996 |  |  |  | • |
| Tarnasky, 1998 | • | • | • | • |
| Patel, 1999 |  |  |  | • |
| Fazel, 2003 | • | • | • | • |
| Harewood, 2005 | • | • |  | • |
| Ito, 2010 | • | • |  | • |
| Pan, 2011 | • | • |  | • |
| Sofuni, 2011 | • | • | • | • |
| Cha, 2012 |  | • | • | • |
| Kawaguchi, 2012 | • | • | • | • |
| Lee, 2012 | • | • | • | • |
| Sofuni, 2007 | • |  | • |  |
| Tsuchiya, 2007 | • |  | • |  |
| **Total** | **10** | **10** | **9** | **12** |
| **Grand Total (N)** | **41** | | | |
| **Rows (r)** | **14** | | | |
| **Columns (c)** | **4** | | | |
| **CCA** | **64.29%** | | | |
| **Degree of overlap** | **Very High overlap** | | | |

**(50)**

| **Topic:** Effect of early precut sphincterotomy (***vs.*** persistent cannulation attempts) on the incidence of PEP in patients with difficult biliary access. | | |
| --- | --- | --- |
| **Systematic Reviews** | **Gong B, 2010** | **Tang Z, 2018** |
| **Primary Studies** |  |  |
| Zhou, 2006 | • | • |
| Cennamo, 2009 | • | • |
| Zagalsky, 2016 |  | • |
| Tang, 2005 | • | • |
| Manes, 2009 | • | • |
| Swan, 2013 |  | • |
| Mariani, 2016 |  | • |
| **Total** | **4** | **7** |
| **Grand Total (N)** | **11** | |
| **Rows (r)** | **7** | |
| **Columns (c)** | **2** | |
| **CCA** | **57.14%** | |
| **Degree of overlap** | **Very High overlap** | |

**(51)**

| **Topic:** Effect of endoscopic papillary balloon dilation (***vs.*** endoscopic sphincterotomy) on the incidence of PEP. | | | |
| --- | --- | --- | --- |
| **Systematic Reviews** | **Xiang ZY, 2023** | **Tringali A, 2019** | **Matsubayashi CO, 2020** |
| **Primary Studies** |  |  |  |
| Minami, 1995 |  | • | • |
| Bergman, 1997 |  | • |  |
| Chen, 1998 |  | • |  |
| lwata, 1998 |  | • |  |
| Ochi, 1999 | • | • | • |
| Arnold, 2001 |  | • | • |
| Bergman, 2001 |  | • | • |
| Yasuda, 2001 |  | • | • |
| Natsui, 2002 |  | • | • |
| Fujita, 2003 |  | • | • |
| Vlavianos, 2003 | • | • | • |
| DiSario, 2004 |  | • | • |
| Tanaka, 2004 |  | • | • |
| Watanabe, 2007 |  | • | • |
| Oh, 2012 |  | • | • |
| Fu, 2013 |  | • | • |
| Minakari, 2013 |  | • | • |
| Seo, 2014 |  | • | • |
| Guo, 2015 |  | • | • |
| Kogure, 2015 |  | • | • |
| Omar, 2017 |  | • | • |
| Dong, 2015 | • |  |  |
| Yasuda, 2010 | • |  |  |
| Li, 2006 | • |  |  |
| El, 2014 | • |  |  |
| Chu, 2016 |  |  | • |
| **Total** | **6** | **21** | **19** |
| **Grand Total (N)** | **46** | | |
| **Rows (r)** | **26** | | |
| **Columns (c)** | **3** | | |
| **CCA** | **38.46%** | | |
| **Degree of overlap** | **Very High overlap** | | |

**(52)**

| **Topic:** Effect of guidewire-assisted cannulation (***vs.*** contrast-assisted cannulation) on the incidence of PEP. | | | | | | | |
| --- | --- | --- | --- | --- | --- | --- | --- |
| **Systematic Reviews** | **Cennamo V, 2009** | **Cheung J, 2009** | **Shao LM, 2009** | **Tse F, 2012** | **de Moura ET, 2016** | **Tse F, 2013** | **Tse F, 2022** |
| **Primary Studies** |  |  |  |  |  |  |  |
| Apostolopoulos, 2005 |  |  |  | • |  | • | • |
| Artifon, 2007 | • | • | • | • | • | • | • |
| Bailey, 2008 |  | • | • | • | • | • | • |
| Gruchy, 2007 |  | • |  | • |  | • | • |
| Katsinelos, 2008 |  | • |  | • | • | • | • |
| Kawakami, 2012 |  |  |  | • | • | • | • |
| Kobayashi, 2010 |  |  |  | • | • | • | • |
| Lee, 2009 | • | • | • | • | • | • | • |
| Lella, 2004 | • | • | • | • | • | • | • |
| Mangiavillano, 2007 |  | • |  | • |  | • | • |
| Mangiavillano, 2011 |  |  |  | • |  | • | • |
| Masci, 2015 |  |  |  |  |  |  | • |
| Nambu, 2011 |  |  |  | • | • | • | • |
| Savadkoohi, 2012 |  |  |  |  | • |  | • |
| Zhang, 2007 |  |  |  |  |  |  | • |
| **Total** | **3** | **7** | **4** | **12** | **9** | **12** | **15** |
| **Grand Total (N)** | **62** | | | | | | |
| **Rows (r)** | **15** | | | | | | |
| **Columns (c)** | **7** | | | | | | |
| **CCA** | **52.22%** | | | | | | |
| **Degree of overlap** | **Very High overlap** | | | | | | |

**(53)**

| **Topic:** Effect of guidewire-assisted cannulation (***vs.*** contrast-assisted cannulation) on the incidence of mild PEP. | | | |
| --- | --- | --- | --- |
| **Systematic Reviews** | **Shao LM, 2009** | **Tse F, 2012** | **Tse F, 2022** |
| **Primary Studies** |  |  |  |
| Apostolopoulos, 2005 |  | • | • |
| Artifon, 2007 | • | • | • |
| Bailey, 2008 | • | • | • |
| Katsinelos, 2008 |  | • | • |
| Kawakami, 2012 |  | • | • |
| Kobayashi, 2010 |  | • | • |
| Lee, 2009 | • | • | • |
| Lella, 2004 | • | • | • |
| Mangiavillano, 2007 |  | • | • |
| Masci, 2015 |  |  | • |
| Nambu, 2011 |  | • | • |
| **Total** | **4** | **10** | **11** |
| **Grand Total (N)** | **25** | | |
| **Rows (r)** | **11** | | |
| **Columns (c)** | **3** | | |
| **CCA** | **63.64%** | | |
| **Degree of overlap** | **Very High overlap** | | |

**(54)**

| **Topic:** Effect of guidewire-assisted cannulation (***vs.*** contrast-assisted cannulation) on the incidence of moderate PEP. | | | |
| --- | --- | --- | --- |
| **Systematic Reviews** | **Shao LM, 2009** | **Tse F, 2012** | **Tse F, 2022** |
| **Primary Studies** |  |  |  |
| Bailey, 2008 | • | • | • |
| Katsinelos, 2008 |  | • | • |
| Kawakami, 2012 |  | • | • |
| Kobayashi, 2010 |  | • | • |
| Lee, 2009 | • | • | • |
| Lella, 2004 | • | • | • |
| Mangiavillano, 2007 |  | • | • |
| Masci, 2015 |  |  | • |
| Nambu, 2011 |  | • | • |
| **Total** | **3** | **8** | **9** |
| **Grand Total (N)** | **20** | | |
| **Rows (r)** | **9** | | |
| **Columns (c)** | **3** | | |
| **CCA** | **61.11%** | | |
| **Degree of overlap** | **Very High overlap** | | |

**(55)**

| **Topic:** Effect of guidewire-assisted cannulation (***vs.*** contrast-assisted cannulation) on the incidence of severe PEP. | | | |
| --- | --- | --- | --- |
| **Systematic Reviews** | **Shao LM, 2009** | **Tse F, 2012** | **Tse F, 2022** |
| **Primary Studies** |  |  |  |
| Katsinelos, 2008 |  | • | • |
| Kawakami, 2012 |  | • | • |
| Kobayashi, 2010 |  | • | • |
| Lee, 2009 | • | • | • |
| Lella, 2004 | • | • | • |
| Mangiavillano, 2007 |  | • | • |
| Masci, 2015 |  |  | • |
| Nambu, 2011 |  | • | • |
| Zhang, 2007 |  |  | • |
| **Total** | **2** | **7** | **9** |
| **Grand Total (N)** | **18** | | |
| **Rows (r)** | **9** | | |
| **Columns (c)** | **3** | | |
| **CCA** | **50.00%** | | |
| **Degree of overlap** | **Very High overlap** | | |

**(56)**

| **Topic:** Effect of the double-guidewire technique (***vs.*** other methods) on the incidence of mild PEP in patients with difficult biliary cannulation. | | |
| --- | --- | --- |
| **Systematic Reviews** | **Tse F, 2016** | **Tse F, 2017** |
| **Primary Studies** |  |  |
| Herreros de Tejada, 2009 | • | • |
| lto, 2010 | • | • |
| Yoo, 2013 | • | • |
| Angsuwatcharakon, 2012 | • | • |
| Cote, 2012 | • | • |
| **Total** | **5** | **5** |
| **Grand Total (N)** | **10** | |
| **Rows (r)** | **5** | |
| **Columns (c)** | **2** | |
| **CCA** | **100.00%** | |
| **Degree of overlap** | **Very High overlap** | |

**(57)**

| **Topic:** Effect of the double-guidewire technique (***vs.*** other methods) on the incidence of moderate PEP in patients with difficult biliary cannulation. | | |
| --- | --- | --- |
| **Systematic Reviews** | **Tse F, 2016** | **Tse F, 2017** |
| **Primary Studies** |  |  |
| Herreros de Tejada, 2009 | • | • |
| Yoo, 2013 | • | • |
| Angsuwatcharakon, 2012 | • | • |
| **Total** | **3** | **3** |
| **Grand Total (N)** | **6** | |
| **Rows (r)** | **3** | |
| **Columns (c)** | **2** | |
| **CCA** | **100.00%** | |
| **Degree of overlap** | **Very High overlap** | |

**(58)**

| **Topic:** Effect of the double-guidewire technique (***vs.*** other methods) on the incidence of severe PEP in patients with difficult biliary cannulation. | | |
| --- | --- | --- |
| **Systematic Reviews** | **Tse F, 2016** | **Tse F, 2017** |
| **Primary Studies** |  |  |
| Herreros de Tejada, 2009 | • | • |
| **Total** | **1** | **1** |
| **Grand Total (N)** | **2** | |
| **Rows (r)** | **1** | |
| **Columns (c)** | **2** | |
| **CCA** | **100.00%** | |
| **Degree of overlap** | **Very High overlap** | |

**(59)**

| **Topic:** Effect of pure cut (***vs.*** blend) on the incidence of PEP. | | |
| --- | --- | --- |
| **Systematic Reviews** | **Funari MP, 2020** | **Verma D, 2007** |
| **Primary Studies** |  |  |
| Elta, 1998 | • | • |
| Stefanidis, 2003 | • | • |
| Macintosh, 2004 | • | • |
| Norton, 2005 |  | • |
| **Total** | **3** | **4** |
| **Grand Total (N)** | **7** | |
| **Rows (r)** | **4** | |
| **Columns (c)** | **2** | |
| **CCA** | **75.00%** | |
| **Degree of overlap** | **Very High overlap** | |

**(60)**

| **Topic:** Effect of pure cut (***vs.*** blend) on the incidence of mild PEP. | | |
| --- | --- | --- |
| **Systematic Reviews** | **Funari MP, 2020** | **Verma D, 2007** |
| **Primary Studies** |  |  |
| Elta, 1998 | • | • |
| Stefanidis, 2003 | • | • |
| Macintosh, 2004 | • | • |
| Norton, 2005 |  | • |
| **Total** | **3** | **4** |
| **Grand Total (N)** | **7** | |
| **Rows (r)** | **4** | |
| **Columns (c)** | **2** | |
| **CCA** | **75.00%** | |
| **Degree of overlap** | **Very High overlap** | |

**(61)**

| **Topic:** Effect of pure cut (***vs.*** blend) on the incidence of moderate PEP. | | |
| --- | --- | --- |
| **Systematic Reviews** | **Funari MP, 2020** | **Verma D, 2007** |
| **Primary Studies** |  |  |
| Elta, 1998 | • | • |
| Macintosh, 2004 | • | • |
| Norton, 2005 |  | • |
| **Total** | **2** | **3** |
| **Grand Total (N)** | **5** | |
| **Rows (r)** | **3** | |
| **Columns (c)** | **2** | |
| **CCA** | **66.67%** | |
| **Degree of overlap** | **Very High overlap** | |

**(62)**

| **Topic:** Effect of pure cut (***vs.*** blend) on the incidence of severe PEP. | | | |
| --- | --- | --- | --- |
| **Systematic Reviews** | **Funari MP, 2020** | **Verma D, 2007** |  |
| **Primary Studies** |  |  |  |
| Elta, 1998 | • | • |  |
| Macintosh, 2004 | • | • |  |
| **Total** | **2** | **2** |  |
| **Grand Total (N)** | **4** | |  |
| **Rows (r)** | **2** | |  |
| **Columns (c)** | **2** | |  |
| **CCA** | **100.00%** | |  |
| **Degree of overlap** | **Very High overlap** | |  |

**(63)**

| **Topic:** Effect of the double-guidewire technique (***vs.*** other methods) on the incidence of PEP in patients with difficult biliary cannulation. | | | | |
| --- | --- | --- | --- | --- |
| **Systematic Reviews** | **Tse F, 2016** | **Guzmán-Calderón E, 2021** | **Tse F, 2017** | **Wang L, 2024** |
| **Primary Studies** |  |  |  |  |
| Arthur Laquière, 2021 |  |  |  | • |
| Fengping Zheng, 2011 |  |  |  | • |
| Fuquan Wang, 2014 |  |  |  | • |
| Herreros, 2009 | • |  | • | • |
| Naoki Sasahira1, 2015 |  |  |  | • |
| Cha, 2012 |  | • |  | • |
| Harutoshi, 2017 |  | • |  | • |
| Jing Chen, 2016 |  |  |  | • |
| Leena, 2021 |  |  |  | • |
| Lin Lu, 2008 |  |  |  | • |
| Mohie, 2019 |  | • |  | • |
| Pan Li, 2017 |  |  |  | • |
| Renhu Sun, 2019 |  |  |  | • |
| Shangjun Tang, 2019 |  |  |  | • |
| Shuai Yuan, 2013 |  |  |  | • |
| Young, 2013 | • | • | • | • |
| Yuliang Wang, 2016 |  |  |  | • |
| Coté GA, 2012 | • |  | • | • |
| Kei ito, 2010 | • |  | • | • |
| Zheng, 2010 | • |  | • |  |
| Angsuwatcharakon, 2012 | • |  | • |  |
| **Total** | **6** | **4** | **6** | **19** |
| **Grand Total (N)** | **35** | | | |
| **Rows (r)** | **21** | | | |
| **Columns (c)** | **4** | | | |
| **CCA** | **22.22%** | | | |
| **Degree of overlap** | **Very High overlap** | | | |

**(64)**

| **Topic:** Effect of pancreatic stent placement (***vs.*** no pancreatic stent placement) on the incidence of PEP. | | | | |
| --- | --- | --- | --- | --- |
| **Systematic Reviews** | **Vadalà di Prampero SF, 2016** | **Zhu G, 2015** | **Mazaki T, 2014** | **Fan JH, 2015** |
| **Primary Studies** |  |  |  |  |
| Cha, 2013 | • | • | • | • |
| Conlglilaro, 2012 |  | • |  |  |
| Fazel, 2003 | • | • | • | • |
| Harewood, 2005 | • | • | • | • |
| lto, 2010 | • | • | • | • |
| Kawagachi, 2012 | • | • | • | • |
| Lee, 2012 | • | • | • | • |
| Pan, 2011 | • | • | • | • |
| Patel, 1999 |  | • | • | • |
| Sheman, 1996 |  | • | • | • |
| Smithline, 1993 | • | • | • | • |
| Sofuni, 2007 | • | • | • | • |
| Sofuni, 2011 | • | • | • | • |
| Tsuchtya, 2007 | • | • | • | • |
| Tarnasky, 1998 | • |  | • | • |
| **Total** | **12** | **14** | **14** | **14** |
| **Grand Total (N)** | **54** | | | |
| **Rows (r)** | **15** | | | |
| **Columns (c)** | **4** | | | |
| **CCA** | **86.67%** | | | |
| **Degree of overlap** | **Very High overlap** | | | |

**Table S4.** Association between pharmacological interventions or procedural technical modifications and the incidence of PEP, graded as not significant.

| **Author, year** | **Intervention** | **Comparator** | **Outcome** | **Studies (Participants)** | **Effect metrics** | **Random effects summary estimate (95% Cl)** | **Random effects**  **p value** | **I^2^ (%)** | **95% prediction interval** | **Random effects estimate of the largest study (95% Cl)** | **Egger's test**  **p value** | **Signifcant studies** | | |
| --- | --- | --- | --- | --- | --- | --- | --- | --- | --- | --- | --- | --- | --- | --- |
|  |  |  |  |  |  |  |  |  |  |  |  | **Observed** | **Expected** | **p value** |
| **Not signifcant (NS)** | | | | | | | | | | | | | | |
| **Pharmacological prevention** | | | | | | | | | | | | | | |
| Cheng, X. et al, 2022 | Aggressive hydration + rectal NSAIDs | rectal NSAIDs | PEP | 3 (1110) | OR | 0.43 (0.12-1.57) | 2.0 x 10^-1^ | 61.5% | 0.00-157987.38 | 0.83 (0.50-1.36) | 0.21 | 1 | 0.50 | 0.42 |
| El Hage Chehade, N. et al, 2023 | Epinephrine + NSAIDs | NSAIDs | PEP | 6 (4016) | OR | 1.00 (0.68, 1.45) | 9.8 x 10^-1^ | 42.2% | 0.38-2.62 | 1.65 (1.04-2.63) | 0.12 | 1 | 4.93 | NP |
| Cao, W. L. et al, 2014 | Allopurinol | Placebo | PEP | 6 (1974) | RR | 0.77 (0.41-1.42) | 4.0 x 10^-1^ | 67.8% | 0.13-4.81 | 1.07 (0.72-1.58) | 0.29 | 1 | 0.38 | 0.32 |
| Fuentes-Orozco, C. et al, 2015 | Antioxidants | Placebo/No intervention | PEP | 12 (3110) | RR | 0.84 (0.59-1.22) | 3.7 x 10^-1^ | 50.8% | 0.31-2.35 | 1.07 (0.72-1.58) | 0.16 | 2 | 0.72 | 0.16 |
| Yuhara, H. et al, 2014 | Ulinastatin | Placebo | PEP | 6 (1926) | RR | 0.71 (0.48-1.05) | 8.3 x 10^-2^ | 4.3% | 0.38-1.32 | 0.96 (0.56-1.65) | 0.19 | 0 | 0.32 | NP |
| Zhang, Y. et al, 2009 | Octreotide | Placebo | PEP | 16 (2721) | OR | 0.81 (0.54-1.23) | 3.2 x 10^-1^ | 25.8% | 0.30-2.19 | 0.45 (0.21-0.95) | 0.55 | 3 | 10.40 | NP |
| Zheng, M. et al, 2008 | Corticosteroid | Placebo | PEP | 7 (2632) | OR | 1.10 (0.77-1.55) | 6.0 x 10^-1^ | 29.9% | 0.50-2.40 | 1.27 (0.91-1.76) | 0.18 | 0 | 1.69 | NP |
| Lyu, Y. et al, 2018 | Diclofenac | Placebo | PEP (High-risk patients) | 4 (671) | RR | 0.63 (0.27-1.50) | 3.0 x 10^-1^ | 69.5% | 0.02-24.48 | 0.97 (0.53-1.80) | 0.61 | 2 | 0.21 | 0.02 |
| Wu, D. et al, 2017 | Aggressive hydration with Lactated Ringer Solution | Standard hydration with Lactated Ringer Solution | PEP (High-risk patients) | 2 (182) | OR | 0.31 (0.09-1.12) | 7.4 x 10^-2^ | 0% | NA | 0.39 (0.10-1.58) | NA | 0 | 1.20 | NP |
| Chen, Z. et al, 2024 | Epinephrine + NSAIDs | NSAIDs | Mild PEP | 4 (2714) | RR | 1.26 (0.82-1.94) | 2.8 x 10^-1^ | 14.0% | 0.31-4.90 | 1.50 (0.93-2.41) | 0.04 | 0 | 2.86 | NP |
| Cao, W. L. et al, 2014 | Allopurinol | Placebo | Mild PEP | 6 (1974) | RR | 1.00 (0.58-1.72) | 1.0 x 10^-0^ | 33.5% | 0.28-3.65 | 1.14 (0.67-1.92) | 0.54 | 0 | 0.60 | NP |
| Fuentes-Orozco, C. et al, 2015 | Antioxidants | Placebo/No intervention | Mild PEP | 9 (2864) | RR | 1.14 (0.82-1.57) | 4.4 x 10^-1^ | 1.6% | 0.77-1.68 | 1.14 (0.67-1.92) | 0.53 | 0 | 0.89 | NP |
| Cao, W. L. et al, 2014 | Allopurinol | Placebo | Moderate PEP | 5 (1804) | RR | 0.75 (0.36-1.57) | 4.5 x 10^-1^ | 27.7% | 0.19-3.33 | 0.97 (0.50-1.92) | 0.19 | 1 | 0.26 | 0.23 |
| Fuentes-Orozco, C. et al, 2015 | Antioxidants | Placebo/No intervention | Moderate PEP | 8 (2694) | RR | 0.98 (0.63-1.53) | 9.2 x 10^-1^ | 0% | 0.56-1.70 | 0.97 (0.50-1.92) | 0.44 | 1 | 0.42 | 0.35 |
| Wang, R. C. et al, 2021 | Aggressive hydration with Lactated Ringer Solution | Standard hydration with Lactated Ringer Solution | Moderate PEP | 4 (895) | OR | 0.66 (0.09-4.62) | 6.7 x 10^-1^ | 55.8% | 0.00-1480.25 | 0.20 (0.02-1.70) | 0.76 | 0 | 3.87 | NP |
| Xie, Y. et al, 2023 | Nafamostat mesylate | Placebo/No treatment | Moderate PEP | 7 (3110) | RR | 0.54 (0.26-1.12) | 9.8 x 10^-2^ | 8.6% | 0.18-1.61 | 0.77 (0.22-2.67) | 0.31 | 1 | 2.20 | NP |
| Cheng, X. et al, 2022 | Aggressive hydration + rectal NSAIDs | rectal NSAIDs | Severe PEP | 2 (909) | OR | 0.32 (0.10-1.08) | 6.7 x 10^-2^ | 0% | NA | 0.32 (0.09-1.18) | NA | 0 | 1.86 | NP |
| Cao, W. L. et al, 2014 | Allopurinol | Placebo | Severe PEP | 4 (1730) | RR | 0.90 (0.28-2.89) | 8.6 x 10^-1^ | 0% | 0.07-11.53 | 0.97 (0.14-6.88) | 0.90 | 0 | 0.21 | NP |
| Fuentes-Orozco, C. et al, 2015 | Antioxidants | Placebo/No intervention | Severe PEP | 6 (2371) | RR | 0.84 (0.31-2.25) | 7.2 x 10^-1^ | 0% | 0.21-3.36 | 0.97 (0.14-6.88) | 0.55 | 0 | 0.32 | NP |
| Serrano, J. P. R. et al, 2020 | NSAIDs | Placebo SN/Placebo lactated Ringer’s solution/Ceftazidime/Glycerin/Glyceryl trinitrate/Saline solution /No treatment | Severe PEP | 7 (3487) | RR | 0.72 (0.38-1.38) | 3.2 x 10^-1^ | 0% | 0.31-1.68 | 0.58 (0.20-1.71) | 0.71 | 0 | 6.00 | NP |
| Xie, Y. et al, 2023 | Nafamostat mesylate | Placebo/No treatment | Severe PEP | 3 (1419) | RR | 0.92 (0.24-3.50) | 9.0 x 10^-1^ | 0% | 0.00-5878.52 | 0.92 (0.13-6.38) | 0.48 | 0 | 0.23 | NP |
| Zheng, M. et al, 2008 | Corticosteroid | Placebo | Severe PEP | 4 (2029) | OR | 1.50 (0.67-3.36) | 3.2 x 10^-1^ | 0% | 0.26-8.80 | 1.21 (0.37-4.00) | 0.08 | 0 | 0.87 | NP |
| Zheng, M. H. et al, 2008 | Gabexate mesilate | Placebo/No treatment | Severe PEP | 4 (2216) | OR | 1.40 (0.34-5.73) | 6.4 x 10^-1^ | 0% | 0.06-31.43 | 0.99 (0.06-15.94) | 0.03 | 0 | 0.20 | NP |
| Chen, Z. et al, 2024 | Epinephrine + NSAIDs | NSAIDs | Moderate to severe PEP | 4 (2714) | RR | 0.73 (0.30-1.76) | 4.8 x 10^-1^ | 43.9% | 0.03-16.96 | 0.55 (0.28-1.07) | 0.95 | 0 | 3.42 | NP |
| Zhang, X. et al, 2024 | Glyceryl Trinitrate + NSAIDs | NSAIDs | Non-mild PEP | 2 (1186) | RR | 0.43 (0.17-1.11) | 8.0 x 10^-2^ | 0% | NA | 0.40 (0.13-1.26) | NA | 0 | 1.99 | NP |
| Chen, Z. et al, 2024 | Epinephrine | Placebo/No treatment | Moderate to severe PEP | 2 (1311) | RR | 0.38 (0.11-1.29) | 1.2 x 10^-1^ | 0% | NA | 0.39 (0.10-1.46) | NA | 0 | 2.00 | NP |
| Ding, J. et al, 2013 | Glyceryl Trinitrate | Placebo/Physiological saline/Vitamin C/No intervention | Moderate to severe PEP | 5 (1621) | RR | 0.70 (0.42-1.15) | 1.6 x 10^-1^ | 0% | 0.31-1.58 | 0.71 (0.36-1.38) | 0.58 | 0 | 1.84 | NP |
| El Kurdi, B. et al, 2024 | Diclofenac | No NSAIDs | Moderate to severe PEP | 10 (2349) | OR | 0.74 (0.43-1.26) | 2.7 x 10^-1^ | 0% | 0.39-1.39 | 0.86 (0.31-2.42) | 0.55 | 0 | 0.97 | NP |
| Narumi, K. et al, 2023 | Nafamostat mesylate | Placebo/No treatment | Moderate to severe PEP | 7 (3110) | RR | 0.61 (0.33-1.15) | 1.3 x 10^-1^ | 5.7% | 0.28-1.38 | 0.92 (0.31-2.69) | 0.06 | 1 | 0.53 | 0.42 |
| **Procedural technical modifications** | | | | | | | | | | | | | | |
| Aziz, Muhammad et al, 2022 | 0.035-inch guidewire | 0.025-inch guidewire | PEP | 3 (1061) | RR | 1.15 (0.72-1.81) | 5.6 x 10^-1^ | 0% | 0.06-22.24 | 1.18 (0.72-1.92) | 0.38 | 0 | 0.41 | NP |
| Choudhary, A. et al, 2014 | Early precut sphincterotomy | Persistent cannulation attempts | PEP | 7 (1032) | OR | 0.63 (0.34-1.18) | 1.5 x 10^-1^ | 0% | 0.28-1.46 | 1.20 (0.37-3.90) | 0.53 | 1 | 0.64 | 0.49 |
| de Clemente Junior, C. C. et al, 2018 | Endoscopic sphincterotomy associated with balloon dilation | Endoscopic sphincterotomy | PEP | 9 (1682) | RR | 0.82 (0.55-1.24) | 3.5 x 10^-1^ | 0% | 0.50-1.35 | 0.79 (0.38-1.66) | 0.31 | 0 | 1.29 | NP |
| Liu, P. et al, 2019 | Endoscopic papillary large balloon dilation | Endoscopic papillary large balloon dilation + Endoscopic sphincterotomy | PEP | 7 (736) | OR | 0.88 (0.43-1.78) | 7.2 x 10^-1^ | 0% | 0.35-2.23 | 0.60 (0.14-2.65) | 0.38 | 0 | 2.05 | NP |
| Tringali, Alberto et al, 2019 | Endoscopic papillary balloon dilation | Endoscopic sphincterotomy | PEP | 21 (2764) | OR | 1.36 (0.89-2.07) | 1.6 x 10^-1^ | 29.3% | 0.43-4.26 | 1.32 (0.47-3.74) | 0.73 | 4 | 2.97 | 0.53 |
| Martins, O. C. et al, 2024 | Endoscopic sphincterotomy | Non-Endoscopic sphincterotomy | PEP | 6 (938) | OR | 0.46 (0.19-1.09) | 7.9 x 10^-2^ | 59.2% | 0.04-5.62 | 0.16 (0.07-0.36) | 0.32 | 2 | 5.93 | NP |
| Mutneja, H. R. et al, 2021 | Needle knife fistulotomy | Conventional cannulation technique | PEP | 3 (491) | OR | 0.22 (0.04-1.04) | 5.7 x 10^-2^ | 33.2% | 0.00-85799.95 | 0.70 (0.11-4.27) | 0.31 | 1 | 0.72 | 0.56 |
| Li, D. F. et al, 2019 | Endocut | Blend | PEP | 3 (594) | RR | 0.64 (0.25-1.64) | 3.5 x 10^-1^ | 0% | 0.00-286.77 | 0.72 (0.23-2.23) | 0.94 | 0 | 0.73 | NP |
| Verma, Dharmendra et al, 2007 | Pure cut | Blend | PEP | 4 (804) | RR | 0.47 (0.18-1.20) | 1.1 x 10^-1^ | 47.1% | 0.01-14.99 | 1.26 (0.50-3.16) | 0.18 | 0 | 0.59 | NP |
| Lyu, Y. et al, 2023 | Needle-knife precut | Transpancreatic sphincterotomy | PEP (Patients with difficult cannulation) | 3 (264) | OR | 2.42 (0.72-8.20) | 1.6 x 10^-1^ | 0% | 0.00-6602.20 | 1.57 (0.24-10.24) | 0.19 | 0 | 0.63 | NP |
| Wang, L. et al, 2024 | Double-guidewire technique | Other methods  (Standard cannulation techniques, Pancreatic stent-assisted technique and Transpancreatic sphincterotomy technique) | PEP (Patients with difficult biliary cannulation) | 19 (1955) | RR | 0.99 (0.64-1.53) | 9.6 x 10^-1^ | 46.1% | 0.26-3.74 | 1.17 (0.71-1.94) | 0.14 | 3 | 137 | 0.15 |
| Tse, F. et al, 2017 | Double-guidewire technique | Other methods  (Persistent attempts with conventional cannulation techniques, Precut sphincterotomy, Pancreatic duct stent placement) | Mild PEP (Patients with difficult biliary cannulation) | 5 (460) | RR | 2.17 (1.00-4.72) | 5.0 x 10^-2^ | 27.8% | 0.31-15.11 | 2.35 (0.76-7.21) | 0.67 | 2 | 2.81 | NP |
| Tse, F. et al, 2017 | Double-guidewire technique | Other methods  (Persistent attempts with conventional cannulation techniques, Precut sphincterotomy, Pancreatic duct stent placement) | Moderate PEP (Patients with difficult biliary cannulation) | 3 (303) | RR | 0.95 (0.27-3.38) | 9.4 x 10^-1^ | 0% | 0.00-3565.64 | 0.63 (0.11-3.66) | 0.88 | 0 | 0.73 | NP |
| Tse, F. et al, 2017 | Double-guidewire technique | Other methods  (Persistent attempts with conventional cannulation techniques, Precut sphincterotomy, Pancreatic duct stent placement) | Severe PEP (Patients with difficult biliary cannulation) | 1 (188) | RR | 1.88 (0.17-20.34) | 6.1 x 10^-1^ | NA | NA | 1.88 (0.17-20.34) | NA | 0 | 0.66 | NP |
| Funari, M. P. et al, 2020 | Endocut | Blend | Mild PEP | 2 (460) | RR | 1.32 (0.26-6.63) | 7.4 x 10^-1^ | 48.1% | NA | 0.72 (0.23-2.23) | NA | 0 | 0.55 | NP |
| Verma, Dharmendra et al, 2007 | Pure cut | Blend | Mild PEP | 4 (804) | RR | 0.54 (0.26-1.12) | 1.0 x 10^-1^ | 0% | 0.11-2.65 | 1.12 (0.33-3.77) | 0.67 | 0 | 0.29 | NP |
| Bicudo de Oliveira, L. et al, 2024 | Endocut | Pure cut | Moderate PEP | 3 (903) | RR | 3.22 (0.68-15.33) | 1.4 x 10^-1^ | 0% | 0.00-80092.66 | 2.94 (0.31-28.04) | 0.69 | 0 | 2.74 | NP |
| Funari, M. P. et al, 2020 | Endocut | Blend | Moderate PEP | 1 (360) | RR | 3.03 (0.12-73.97) | 5.0 x 10^-1^ | NA | NA | 3.03 (0.12-73.97) | NA | 0 | 1.00 | NP |
| Verma, Dharmendra et al, 2007 | Pure cut | Blend | Moderate PEP | 3 (683) | RR | 0.43 (0.11-1.70) | 2.3 x 10^-1^ | 0% | 0.00-2856.83 | 0.75 (0.13-4.39) | 0.004 | 0 | 0.67 | NP |
| Tse, F. et al, 2022 | Guidewire-assisted cannulation | Contrast-assisted cannulation | Moderate PEP | 9 (2876) | RR | 0.76 (0.38-1.52) | 4.3 x 10^-1^ | 0% | 0.33-1.77 | 0.75 (0.17-3.30) | 0.04 | 0 | 2.64 | NP |
| Tse, F. et al, 2022 | Guidewire-assisted cannulation | Contrast-assisted cannulation | Severe PEP | 9 (2959) | RR | 0.69 (0.27-1.81) | 4.5 x 10^-1^ | 0% | 0.21-2.21 | 4.04 (0.46-35.83) | 0.09 | 0 | 9.00 | NP |
| Bicudo de Oliveira, L. et al, 2024 | Endocut | Pure cut | Severe PEP | 2 (170) | RR | 3.16 (0.53-18.71) | 2.1 x 10^-1^ | 0% | NA | 4.20 (0.49-35.98) | NA | 0 | 1.89 | NP |
| Verma, Dharmendra et al, 2007 | Pure cut | Blend | Severe PEP | 2 (416) | RR | 1.41 (0.09-23.03) | 8.1 x 10^-1^ | 38.1% | NA | 5.60 (0.27-115.47) | NA | 0 | 2.00 | NP |

NA=not available.

NP=not pertinent (because the number of expecied significan studies was larger than the number of observed significant studies).

**Table S5.** Association between pharmacological interventions or procedural technical modifications and the incidence of PEP: a standardized analysis using relative risk.

| **Author, year** | **Intervention** | **Comparator** | **Outcome** | **Studies (Participants)** | **Effect metrics** | **Random effects summary estimate (95% Cl)** | **Random effects**  **p value** | **I^2^ (%)** | **95% prediction interval** | **Random effects estimate of the largest study (95% Cl)** | **Egger's test**  **p value** | **Signifcant studies** | | |
| --- | --- | --- | --- | --- | --- | --- | --- | --- | --- | --- | --- | --- | --- | --- |
|  |  |  |  |  |  |  |  |  |  |  |  | **Observed** | **Expected** | **p value** |
| **Highly suggestive (Class II)** | | | | | | | | | | | | | | |
| Zhu, G. et al, 2015 | Pancreatic stent placement | No Pancreatic stent placement | PEP | 14 (1469) | RR | 0.37 (0.27-0.51) | 5.6 x 10^-10^ | 0% | 0.26-0.52 | 0.52 (0.29-0.92) | 0.001 | 6 | 5.54 | 0.79 |
| **Suggestive (Class III)** | | | | | | | | | | | | | | |
| **Pharmacological prevention** | | | | | | | | | | | | | | |
| El Kurdi, B. et al, 2024 | Indomethacin | No NSAIDs | PEP | 10 (4164) | RR | 0.56 (0.42-0.73) | 2.4 x 10^-5^ | 35.4% | 0.29-1.08 | 0.54 (0.35-0.84) | 0.58 | 6 | 8.60 | NP |
| Iqbal, U. et al, 2020 | Epinephrine | Placebo/No treatment | PEP | 3 (1456) | RR | 0.34 (0.18-0.62) | 3.1 x 10^-4^ | 0% | 0.01-15.99 | 0.30 (0.15-0.63) | 0.80 | 1 | 2.98 | NP |
| Narumi, K. et al, 2023 | Nafamostat mesylate | Placebo/No treatment | PEP | 8 (3210) | RR | 0.50 (0.38-0.66) | 1.1 x 10^-6^ | 0% | 0.35-0.71 | 0.35 (0.20-0.62) | 0.89 | 4 | 7.61 | NP |
| Serrano, J. P. R. et al, 2020 | NSAIDs | Placebo SN/Placebo lactated Ringer’s solution/Ceftazidime/Glycerin/Glyceryl trinitrate/Saline solution /No treatment | PEP | 26 (8143) | RR | 0.65 (0.52-0.81) | 1.1 x 10^-4^ | 52.9% | 0.28-1.50 | 0.54 (0.35-0.84) | 0.19 | 11 | 19.13 | NP |
| Wu, M. et al, 2021 | Aggressive hydration with Lactated Ringer Solution | Standard hydration with Lactated Ringer Solution | PEP | 10 (2200) | RR | 0.44 (0.29-0.66) | 8.9 x 10^-5^ | 32.4% | 0.17-1.13 | 0.39 (0.20-0.78) | 0.48 | 5 | 7.59 | NP |
| Wan, J. et al, 2017 | Indomethacin | Placebo | PEP (High-risk patients) | 3 (1161) | RR | 0.46 (0.32-0.65) | 6.9 x 10^-6^ | 3.9% | 0.05-4.13 | 0.54 (0.35-0.84) | 0.05 | 3 | 2.49 | 1.00 |
| Hou, Y. C. et al, 2017 | NSAIDs | Placebo/Glycerin/No treatment | Mild PEP | 14 (6208) | RR | 0.60 (0.47-0.77) | 7.1 x 10^-5^ | 18.9% | 0.35-1.02 | 0.47 (0.29-0.78) | 0.68 | 3 | 12.05 | NP |
| Narumi, K. et al, 2023 | Nafamostat mesylate | Placebo/No treatment | Mild PEP | 7 (3110) | RR | 0.49 (0.36-0.69) | 2.6 x 10^-5^ | 0% | 0.32-0.76 | 0.46 (0.25-0.84) | 0.63 | 3 | 6.32 | NP |
| **Procedural technical modifications** | | | | | | | | | | | | | | |
| Tse, F. et al, 2022 | Guidewire-assisted cannulation | Contrast-assisted cannulation | PEP | 15 (4426) | RR | 0.51 (0.36-0.72) | 1.3 x 10^-4^ | 35.5% | 0.21-1.26 | 1.23 (0.61-2.50) | 0.04 | 4 | 2.44 | 0.29 |
| Mazaki, T. et al, 2014 | Pancreatic stent placement | No Pancreatic stent placement | PEP (High-risk patients) | 12 (1276) | RR | 0.41 (0.30-0.56) | 2.5 x 10^-8^ | 0% | 0.29-0.59 | 0.65 (0.38-1.10) | 0.000 | 6 | 2.65 | 0.03 |
| da Cruz Portela, Joaquim Coelho et al, 2019 | Pancreatic stent placement | No Pancreatic stent placement | Mild PEP (High-risk patients) | 9 (1249) | RR | 0.48 (0.32-0.73) | 5.3 x 10^-4^ | 0% | 0.29-0.79 | 0.52 (0.26-1.02) | 0.25 | 1 | 4.44 | NP |
| Fan, J. H. et al, 2015 | Pancreatic stent placement | No Pancreatic stent placement | Mild PEP | 13 (1501) | RR | 0.45 (0.32-0.62) | 7.0 x 10^-7^ | 0% | 0.31-0.63 | 0.67 (0.39-1.14) | 0.003 | 4 | 2.77 | 0.49 |
| Mazaki, T. et al, 2014 | Pancreatic stent placement | No Pancreatic stent placement | Mild to moderate PEP | 13 (1501) | RR | 0.45 (0.32-0.62) | 8.0 x 10^-7^ | 0% | 0.31-0.64 | 0.67 (0.39-1.14) | 0.003 | 4 | 2.77 | 0.49 |
| **Weak (Class lV)** | | | | | | | | | | | | | | |
| **Pharmacological prevention** | | | | | | | | | | | | | | |
| Chiu, Y. J. et al, 2021 | Gabexate mesilate | Placebo/No treatment | PEP | 12 (3662) | RR | 0.56 (0.37-0.86) | 7.8 x 10^-3^ | 40.8% | 0.18-1.70 | 1.20 (0.66-2.18) | 0.02 | 3 | 1.71 | 0.40 |
| Ding, J. et al, 2013 | Glyceryl Trinitrate | Placebo/Physiological saline/Vitamin C/No intervention | PEP | 11 (2395) | RR | 0.68 (0.51-0.90) | 6.5 x 10^-3^ | 5.1% | 0.46-1.01 | 0.63 (0.35-1.11) | 0.77 | 1 | 4.47 | NP |
| Merchan, M. F. S. et al, 2022 | Antibiotics | Non-antibiotics | PEP | 4 (698) | RR | 0.51 (0.27-0.97) | 3.9 x 10^-2^ | 0% | 0.13-2.13 | 0.69 (0.26-1.80) | 0.64 | 1 | 1.05 | NP |
| Serrano, J. P. R. et al, 2020 | Diclofenac | Placebo SN/Placebo lactated Ringer’s solution/Ceftazidime/Glycerin/Glyceryl trinitrate/Saline solution /No treatment | PEP | 14 (3501) | RR | 0.70 (0.49-0.98) | 3.8 x 10^-2^ | 59.6% | 0.23-2.08 | 0.60 (0.38-0.93) | 0.08 | 5 | 7.40 | NP |
| Wang, G. et al, 2018 | Somatostatin | Placebo/No treatment | PEP | 14 (4593) | RR | 0.63 (0.44-0.90) | 1.2 x 10^-2^ | 55.6% | 0.20-1.95 | 0.53 (0.30-0.92) | 0.13 | 5 | 10.02 | NP |
| Zhang, X. et al, 2024 | Glyceryl Trinitrate + NSAIDs | NSAIDs | PEP | 3 (1254) | RR | 0.53 (0.36-0.78) | 1.4 x 10^-3^ | 0% | 0.04-6.62 | 0.59 (0.37-0.95) | 0.30 | 2 | 1.92 | 1.00 |
| Lyu, Y. et al, 2018 | NSAIDs | Placebo | PEP (High-risk patients) | 6 (1439) | RR | 0.54 (0.31-0.94) | 2.8 x 10^-2^ | 62.8% | 0.10-2.79 | 0.54 (0.35-0.84) | 0.63 | 4 | 3.89 | 1.00 |
| Wang, G. et al, 2018 | Somatostatin | Placebo/No treatment | PEP (High-risk patients) | 3 (1114) | RR | 0.67 (0.48-0.95) | 2.3 x 10^-2^ | 0% | 0.07-6.32 | 0.57 (0.32-1.03) | NA | 0 | 1.87 | NP |
| Xie, Y. et al, 2023 | Nafamostat mesylate | Placebo/No treatment | PEP (High-risk patients) | 6 (1294) | RR | 0.57 (0.35-0.94) | 2.7 x 10^-2^ | 40.6% | 0.16-2.07 | 0.99 (0.53-1.87) | 0.25 | 2 | 0.30 | 0.03 |
| Chen, Z. et al, 2024 | Epinephrine | Placebo/No treatment | Mild PEP | 2 (1311) | RR | 0.26 (0.11-0.60) | 1.8 x 10^-3^ | 0% | NA | 0.27 (0.11-0.66) | NA | 1 | 2.00 | NP |
| Wan, J. et al, 2017 | Indomethacin | Placebo | Mild PEP | 7 (3013) | RR | 0.61 (0.40-0.93) | 2.2 x 10^-2^ | 50.0% | 0.19-1.95 | 0.45 (0.25-0.81) | 0.53 | 3 | 6.56 | NP |
| Serrano, J. P. R. et al, 2020 | NSAIDs | Placebo SN/Placebo lactated Ringer’s solution/Ceftazidime/Glycerin/Glyceryl trinitrate/Saline solution /No treatment | Moderate PEP | 11 (4284) | RR | 0.62 (0.44-0.89) | 9.5 x 10^-3^ | 3.5% | 0.40-0.99 | 0.63 (0.33-1.17) | 0.92 | 1 | 7.18 | NP |
| El Kurdi, B. et al, 2024 | NSAIDs | No NSAIDs | Moderate to severe PEP | 19 (6281) | RR | 0.59 (0.42-0.82) | 1.7 x 10^-3^ | 0% | 0.41-0.84 | 0.50 (0.26-0.95) | 0.70 | 1 | 16.12 | NP |
| El Kurdi, B. et al, 2024 | Indomethacin | No NSAIDs | Moderate to severe PEP | 8 (3608) | RR | 0.51 (0.32-0.82) | 4.9 x 10^-3^ | 0% | 0.28-0.92 | 0.50 (0.26-0.95) | 0.66 | 1 | 7.45 | NP |
| Wu, D. et al, 2017 | Aggressive hydration with Lactated Ringer Solution | Standard hydration with Lactated Ringer Solution | Moderate to severe PEP | 3 (722) | RR | 0.49 (0.35-0.68) | 1.7 x 10^-5^ | 0% | 0.06-4.06 | 0.52 (0.32-0.84) | 0.73 | 2 | 1.85 | 1.00 |
| **Procedural technical modifications** | | | | | | | | | | | | | | |
| Bicudo de Oliveira, L. et al, 2024 | Endocut | Pure cut | PEP | 4 (987) | RR | 3.00 (1.40-6.45) | 4.9 x 10^-3^ | 0% | 0.56-16.04 | 2.61 (1.04-6.57) | 0.12 | 1 | 3.31 | NP |
| Yang, H. et al, 2022 | Covered self-expandable metallic stents | Multiple plastic stents | PEP | 4 (1154) | RR | 3.06 (1.21-7.72) | 1.8 x 10^-2^ | 7.7% | 0.30-31.55 | 6.27 (1.72-22.81) | 0.80 | 1 | 4.00 | NP |
| Tang, Z. et al, 2018 | Early precut sphincterotomy | Persistent cannulation attempts | PEP (Patients with difficult biliary access) | 7 (999) | RR | 0.57 (0.36-0.92) | 2.1 x 10^-2^ | 0% | 0.31-1.06 | 0.45 (0.22-0.91) | 0.92 | 2 | 4.38 | NP |
| da Cruz Portela, Joaquim Coelho et al, 2019 | Pancreatic stent placement | No Pancreatic stent placement | Moderate PEP (High-risk patients) | 8 (1129) | RR | 0.35 (0.17-0.72) | 4.8 x 10^-3^ | 0% | 0.14-0.89 | 0.48 (0.15-1.56) | 0.22 | 0 | 4.53 | NP |
| da Cruz Portela, Joaquim Coelho et al, 2019 | Pancreatic stent placement | No Pancreatic stent placement | Severe PEP (High-risk patients) | 7 (932) | RR | 0.31 (0.10-0.97) | 4.5 x 10^-2^ | 0% | 0.07-1.39 | 0.14 (0.01-2.53) | 0.27 | 0 | 6.98 | NP |
| Bicudo de Oliveira, L. et al, 2024 | Endocut | Pure cut | Mild PEP | 4 (987) | RR | 2.53 (1.24-5.16) | 1.0 x 10^-2^ | 0% | 0.53-12.14 | 2.52 (0.97-6.52) | 0.29 | 0 | 3.24 | NP |
| Tse, F. et al, 2022 | Guidewire-assisted cannulation | Contrast-assisted cannulation | Mild PEP | 11 (3306) | RR | 0.47 (0.26-0.83) | 9.8 x 10^-3^ | 49.0% | 0.10-2.24 | 1.44 (0.63-3.31) | 0.08 | 3 | 4.50 | NP |
| Zhu, G. et al, 2015 | Pancreatic stent placement | No Pancreatic stent placement | Moderate PEP | 8 (1087) | RR | 0.37 (0.17-0.79) | 1.0 x 10^-2^ | 0% | 0.14-0.96 | 0.52 (0.16-1.72) | 0.08 | 0 | 3.78 | NP |
| Fan, J. H. et al, 2015 | Pancreatic stent placement | No Pancreatic stent placement | Severe PEP | 8 (1009) | RR | 0.26 (0.09-0.76) | 1.4 x 10^-2^ | 0% | 0.06-1.00 | 0.13 (0.01-2.41) | 0.000 | 0 | 7.90 | NP |
| Matsubayashi, C. O. et al, 2020 | Endoscopic papillary balloon dilation | Endoscopic sphincterotomy | Severe PEP | 6 (1019) | RR | 3.32 (1.07-10.29) | 3.7 x 10^-2^ | 0% | 0.67-16.47 | 2.00 (0.19-21.62) | 0.33 | 0 | 4.04 | NP |
| **Not signifcant (NS)** | | | | | | | | | | | | | | |
| **Pharmacological prevention** | | | | | | | | | | | | | | |
| Cheng, X. et al, 2022 | Aggressive hydration + rectal NSAIDs | rectal NSAIDs | PEP | 3 (1110) | RR | 0.47 (0.14-1.56) | 2.1 x 10^-1^ | 60.7% | 0.00-100935.32 | 0.84 (0.53-1.33) | 0.21 | 1 | 0.46 | 0.39 |
| El Hage Chehade, N. et al, 2023 | Epinephrine + NSAIDs | NSAIDs | PEP | 6 (4016) | RR | 1.00 (0.71-1.42) | 9.2 x 10^-1^ | 41.2% | 0.37-2.59 | 1.60 (1.03-2.47) | 0.10 | 1 | 4.77 | NP |
| Cao, W. L. et al, 2014 | Allopurinol | Placebo | PEP | 6 (1974) | RR | 0.77 (0.41-1.42) | 4.0 x 10^-1^ | 67.8% | 0.13-4.81 | 1.07 (0.72-1.58) | 0.29 | 1 | 0.38 | 0.32 |
| Fuentes-Orozco, C. et al, 2015 | Antioxidants | Placebo/No intervention | PEP | 12 (3110) | RR | 0.84 (0.59-1.22) | 3.7 x 10^-1^ | 50.8% | 0.31-2.35 | 1.07 (0.72-1.58) | 0.16 | 2 | 0.72 | 0.16 |
| Yuhara, H. et al, 2014 | Ulinastatin | Placebo | PEP | 6 (1926) | RR | 0.71 (0.48-1.05) | 8.3 x 10^-2^ | 4.3% | 0.38-1.32 | 0.96 (0.56-1.65) | 0.19 | 0 | 0.32 | NP |
| Zhang, Y. et al, 2009 | Octreotide | Placebo | PEP | 16 (2721) | RR | 0.83 (0.57-1.21) | 3.2 x 10^-1^ | 24.6% | 0.33-2.04 | 0.75 (0.36-1.57) | 0.79 | 2 | 2.79 | NP |
| Zheng, M. et al, 2008 | Corticosteroid | Placebo | PEP | 7 (2632) | RR | 1.09 (0.81-1.48) | 6.0 x 10^-1^ | 27.4% | 0.54-2.17 | 1.22 (0.92-1.62) | 0.19 | 0 | 1.29 | NP |
| Lyu, Y. et al, 2018 | Diclofenac | Placebo | PEP (High-risk patients) | 4 (671) | RR | 0.63 (0.27-1.50) | 3.0 x 10^-1^ | 69.5% | 0.02-24.48 | 0.97 (0.53-1.80) | 0.61 | 2 | 0.21 | 0.02 |
| Wu, D. et al, 2017 | Aggressive hydration with Lactated Ringer Solution | Standard hydration with Lactated Ringer Solution | PEP (High-risk patients) | 2 (182) | RR | 0.34 (0.10-1.13) | 6.8 x 10^-2^ | 0% | NA | 0.42 (0.11-1.55) | NA | 0 | 1.10 | NP |
| Chen, Z. et al, 2024 | Epinephrine + NSAIDs | NSAIDs | Mild PEP | 4 (2714) | RR | 1.26 (0.82-1.94) | 2.8 x 10^-1^ | 14.0% | 0.31-4.90 | 1.50 (0.93-2.41) | 0.04 | 0 | 2.86 | NP |
| Cao, W. L. et al, 2014 | Allopurinol | Placebo | Mild PEP | 6 (1974) | RR | 1.00 (0.58-1.72) | 1.0 x 10^-0^ | 33.5% | 0.28-3.65 | 1.14 (0.67-1.92) | 0.54 | 0 | 0.60 | NP |
| Fuentes-Orozco, C. et al, 2015 | Antioxidants | Placebo/No intervention | Mild PEP | 9 (2864) | RR | 1.14 (0.82-1.57) | 4.4 x 10^-1^ | 1.6% | 0.77-1.68 | 1.14 (0.67-1.92) | 0.53 | 0 | 0.89 | NP |
| Cao, W. L. et al, 2014 | Allopurinol | Placebo | Moderate PEP | 5 (1804) | RR | 0.75 (0.36-1.57) | 4.5 x 10^-1^ | 27.7% | 0.19-3.33 | 0.97 (0.50-1.92) | 0.19 | 1 | 0.26 | 0.23 |
| Fuentes-Orozco, C. et al, 2015 | Antioxidants | Placebo/No intervention | Moderate PEP | 8 (2694) | RR | 0.98 (0.63-1.53) | 9.2 x 10^-1^ | 0% | 0.56-1.70 | 0.97 (0.50-1.92) | 0.44 | 1 | 0.42 | 0.35 |
| Wang, R. C. et al, 2021 | Aggressive hydration with Lactated Ringer Solution | Standard hydration with Lactated Ringer Solution | Moderate PEP | 4 (895) | RR | 0.67 (0.10-4.36) | 6.9 x 10^-1^ | 53.9% | 0.00-933.58 | 0.20 (0.02-1.70) | 0.76 | 0 | 3.87 | NP |
| Xie, Y. et al, 2023 | Nafamostat mesylate | Placebo/No treatment | Moderate PEP | 7 (3110) | RR | 0.54 (0.26-1.12) | 9.8 x 10^-2^ | 8.6% | 0.18-1.61 | 0.77 (0.22-2.67) | 0.31 | 1 | 2.20 | NP |
| Cheng, X. et al, 2022 | Aggressive hydration + rectal NSAIDs | rectal NSAIDs | Severe PEP | 2 (909) | RR | 0.33 (0.10-1.08) | 7.1 x 10^-2^ | 0% | NA | 0.33 (0.09-1.19) | NA | 0 | 1.84 | NP |
| Cao, W. L. et al, 2014 | Allopurinol | Placebo | Severe PEP | 4 (1730) | RR | 0.90 (0.28-2.89) | 8.6 x 10^-1^ | 0% | 0.07-11.53 | 0.97 (0.14-6.88) | 0.90 | 0 | 0.21 | NP |
| Fuentes-Orozco, C. et al, 2015 | Antioxidants | Placebo/No intervention | Severe PEP | 6 (2371) | RR | 0.84 (0.31-2.25) | 7.2 x 10^-1^ | 0% | 0.21-3.36 | 0.97 (0.14-6.88) | 0.55 | 0 | 0.32 | NP |
| Serrano, J. P. R. et al, 2020 | NSAIDs | Placebo SN/Placebo lactated Ringer’s solution/Ceftazidime/Glycerin/Glyceryl trinitrate/Saline solution /No treatment | Severe PEP | 7 (3487) | RR | 0.72 (0.38-1.38) | 3.2 x 10^-1^ | 0% | 0.31-1.68 | 0.58 (0.20-1.71) | 0.71 | 0 | 6.00 | NP |
| Xie, Y. et al, 2023 | Nafamostat mesylate | Placebo/No treatment | Severe PEP | 3 (1419) | RR | 0.92 (0.24-3.50) | 9.0 x 10^-1^ | 0% | 0.00-5878.52 | 0.92 (0.13-6.38) | 0.48 | 0 | 0.23 | NP |
| Zheng, M. et al, 2008 | Corticosteroid | Placebo | Severe PEP | 4 (2029) | RR | 1.48 (0.67-3.25) | 3.3 x 10^-1^ | 0% | 0.26-8.39 | 1.21 (0.37-3.94) | 0.08 | 0 | 0.87 | NP |
| Zheng, M. H. et al, 2008 | Gabexate mesilate | Placebo/No treatment | Severe PEP | 4 (2216) | RR | 1.40 (0.34-5.68) | 6.3 x 10^-1^ | 0% | 0.06-31.14 | 0.99 (0.06-15.74) | 0.03 | 0 | 0.20 | NP |
| Chen, Z. et al, 2024 | Epinephrine + NSAIDs | NSAIDs | Moderate to severe PEP | 4 (2714) | RR | 0.73 (0.30-1.76) | 4.8 x 10^-1^ | 43.9% | 0.03-16.96 | 0.55 (0.28-1.07) | 0.95 | 0 | 3.42 | NP |
| Zhang, X. et al, 2024 | Glyceryl Trinitrate + NSAIDs | NSAIDs | Non-mild PEP | 2 (1186) | RR | 0.43 (0.17-1.11) | 8.0 x 10^-2^ | 0% | NA | 0.40 (0.13-1.26) | NA | 0 | 1.99 | NP |
| Chen, Z. et al, 2024 | Epinephrine | Placebo/No treatment | Moderate to severe PEP | 2 (1311) | RR | 0.38 (0.11-1.29) | 1.2 x 10^-1^ | 0% | NA | 0.39 (0.10-1.46) | NA | 0 | 2.00 | NP |
| Ding, J. et al, 2013 | Glyceryl Trinitrate | Placebo/Physiological saline/Vitamin C/No intervention | Moderate to severe PEP | 5 (1621) | RR | 0.70 (0.42-1.15) | 1.6 x 10^-1^ | 0% | 0.31-1.58 | 0.71 (0.36-1.38) | 0.58 | 0 | 1.84 | NP |
| El Kurdi, B. et al, 2024 | Diclofenac | No NSAIDs | Moderate to severe PEP | 10 (2349) | RR | 0.75 (0.44-1.26) | 2.8 x 10^-1^ | 0% | 0.41-1.39 | 0.87 (0.32-2.35) | 0.53 | 0 | 0.90 | NP |
| Narumi, K. et al, 2023 | Nafamostat mesylate | Placebo/No treatment | Moderate to severe PEP | 7 (3110) | RR | 0.61 (0.33-1.15) | 1.3 x 10^-1^ | 5.7% | 0.28-1.38 | 0.92 (0.31-2.69) | 0.06 | 1 | 0.53 | 0.42 |
| **Procedural technical modifications** | | | | | | | | | | | | | | |
| Aziz, Muhammad et al, 2022 | 0.035-inch guidewire | 0.025-inch guidewire | PEP | 3 (1061) | RR | 1.15 (0.72-1.81) | 5.6 x 10^-1^ | 0% | 0.06-22.24 | 1.18 (0.72-1.92) | 0.38 | 0 | 0.41 | NP |
| Choudhary, A. et al, 2014 | Early precut sphincterotomy | Persistent cannulation attempts | PEP | 7 (1032) | RR | 0.68 (0.38-1.21) | 1.9 x 10^-1^ | 0% | 0.32-1.45 | 1.16 (0.45-3.02) | 0.40 | 1 | 0.54 | 0.43 |
| de Clemente Junior, C. C. et al, 2018 | Endoscopic sphincterotomy associated with balloon dilation | Endoscopic sphincterotomy | PEP | 9 (1682) | RR | 0.82 (0.55-1.24) | 3.5 x 10^-1^ | 0% | 0.50-1.35 | 0.79 (0.38-1.66) | 0.31 | 0 | 1.29 | NP |
| Liu, P. et al, 2019 | Endoscopic papillary large balloon dilation | Endoscopic papillary large balloon dilation + Endoscopic sphincterotomy | PEP | 7 (736) | RR | 0.87 (0.46-1.65) | 6.6 x 10^-1^ | 0% | 0.38-2.00 | 0.67 (0.21-2.14) | 0.35 | 0 | 1.41 | NP |
| Tringali, Alberto et al, 2019 | Endoscopic papillary balloon dilation | Endoscopic sphincterotomy | PEP | 21 (2764) | RR | 1.31 (0.89-1.93) | 1.7 x 10^-1^ | 27.2% | 0.47-3.63 | 1.29 (0.50-3.28) | 0.64 | 4 | 2.66 | 0.33 |
| Martins, O. C. et al, 2024 | Endoscopic sphincterotomy | Non-Endoscopic sphincterotomy | PEP | 6 (938) | RR | 0.49 (0.23-1.07) | 7.2 x 10^-2^ | 55.9% | 0.06-4.42 | 0.19 (0.09-0.41) | 0.32 | 2 | 5.85 | NP |
| Mutneja, H. R. et al, 2021 | Needle knife fistulotomy | Conventional cannulation technique | PEP | 3 (491) | RR | 0.23 (0.05-1.05) | 5.8 x 10^-2^ | 30.6% | 0.00-91575.05 | 0.70 (0.12-4.13) | 0.24 | 1 | 0.72 | 0.56 |
| Li, D. F. et al, 2019 | Endocut | Blend | PEP | 3 (594) | RR | 0.64 (0.25-1.64) | 3.5 x 10^-1^ | 0% | 0.00-286.77 | 0.72 (0.23-2.23) | 0.94 | 0 | 0.73 | NP |
| Verma, Dharmendra et al, 2007 | Pure cut | Blend | PEP | 4 (804) | RR | 0.47 (0.18-1.20) | 1.1 x 10^-1^ | 47.1% | 0.01-14.99 | 1.26 (0.50-3.16) | 0.18 | 0 | 0.59 | NP |
| Lyu, Y. et al, 2023 | Needle-knife precut | Transpancreatic sphincterotomy | PEP (Patients with difficult cannulation) | 3 (264) | RR | 2.25 (0.72-7.08) | 1.6 x 10^-1^ | 0% | 0.00-3809.28 | 1.50 (0.27-8.25) | 0.20 | 0 | 0.54 | NP |
| Wang, L. et al, 2024 | Double-guidewire technique | Other methods  (Standard cannulation techniques, Pancreatic stent-assisted technique and Transpancreatic sphincterotomy technique) | PEP (Patients with difficult biliary cannulation) | 19 (1955) | RR | 0.99 (0.64-1.53) | 9.6 x 10^-1^ | 46.1% | 0.26-3.74 | 1.17 (0.71-1.94) | 0.14 | 3 | 137 | 0.15 |
| Tse, F. et al, 2017 | Double-guidewire technique | Other methods  (Persistent attempts with conventional cannulation techniques, Precut sphincterotomy, Pancreatic duct stent placement) | Mild PEP (Patients with difficult biliary cannulation) | 5 (460) | RR | 2.17 (1.00-4.72) | 5.0 x 10^-2^ | 27.8% | 0.31-15.11 | 2.35 (0.76-7.21) | 0.67 | 2 | 2.81 | NP |
| Tse, F. et al, 2017 | Double-guidewire technique | Other methods  (Persistent attempts with conventional cannulation techniques, Precut sphincterotomy, Pancreatic duct stent placement) | Moderate PEP (Patients with difficult biliary cannulation) | 3 (303) | RR | 0.95 (0.27-3.38) | 9.4 x 10^-1^ | 0% | 0.00-3565.64 | 0.63 (0.11-3.66) | 0.88 | 0 | 0.73 | NP |
| Tse, F. et al, 2017 | Double-guidewire technique | Other methods  (Persistent attempts with conventional cannulation techniques, Precut sphincterotomy, Pancreatic duct stent placement) | Severe PEP (Patients with difficult biliary cannulation) | 1 (188) | RR | 1.88 (0.17-20.34) | 6.1 x 10^-1^ | NA | NA | 1.88 (0.17-20.34) | NA | 0 | 0.66 | NP |
| Funari, M. P. et al, 2020 | Endocut | Blend | Mild PEP | 2 (460) | RR | 1.32 (0.26-6.63) | 7.4 x 10^-1^ | 48.1% | NA | 0.72 (0.23-2.23) | NA | 0 | 0.55 | NP |
| Verma, Dharmendra et al, 2007 | Pure cut | Blend | Mild PEP | 4 (804) | RR | 0.54 (0.26-1.12) | 1.0 x 10^-1^ | 0% | 0.11-2.65 | 1.12 (0.33-3.77) | 0.67 | 0 | 0.29 | NP |
| Bicudo de Oliveira, L. et al, 2024 | Endocut | Pure cut | Moderate PEP | 3 (903) | RR | 3.22 (0.68-15.33) | 1.4 x 10^-1^ | 0% | 0.00-80092.66 | 2.94 (0.31-28.04) | 0.69 | 0 | 2.74 | NP |
| Funari, M. P. et al, 2020 | Endocut | Blend | Moderate PEP | 1 (360) | RR | 3.03 (0.12-73.97) | 5.0 x 10^-1^ | NA | NA | 3.03 (0.12-73.97) | NA | 0 | 1.00 | NP |
| Verma, Dharmendra et al, 2007 | Pure cut | Blend | Moderate PEP | 3 (683) | RR | 0.43 (0.11-1.70) | 2.3 x 10^-1^ | 0% | 0.00-2856.83 | 0.75 (0.13-4.39) | 0.004 | 0 | 0.67 | NP |
| Tse, F. et al, 2022 | Guidewire-assisted cannulation | Contrast-assisted cannulation | Moderate PEP | 9 (2876) | RR | 0.76 (0.38-1.52) | 4.3 x 10^-1^ | 0% | 0.33-1.77 | 0.75 (0.17-3.30) | 0.04 | 0 | 2.64 | NP |
| Tse, F. et al, 2022 | Guidewire-assisted cannulation | Contrast-assisted cannulation | Severe PEP | 9 (2959) | RR | 0.69 (0.27-1.81) | 4.5 x 10^-1^ | 0% | 0.21-2.21 | 4.04 (0.46-35.83) | 0.09 | 0 | 9.00 | NP |
| Bicudo de Oliveira, L. et al, 2024 | Endocut | Pure cut | Severe PEP | 2 (170) | RR | 3.16 (0.53-18.71) | 2.1 x 10^-1^ | 0% | NA | 4.20 (0.49-35.98) | NA | 0 | 1.89 | NP |
| Verma, Dharmendra et al, 2007 | Pure cut | Blend | Severe PEP | 2 (416) | RR | 1.41 (0.09-23.03) | 8.1 x 10^-1^ | 38.1% | NA | 5.60 (0.27-115.47) | NA | 0 | 2.00 | NP |

NA=not available.

NP=not pertinent (because the number of expecied significan studies was larger than the number of observed significant studies).

**Table S6.** Quality assessment of evidence using the GRADE framework.

| **Certainty assessment** | | | | | | | | **№ of patients** | | **Effect** | | **Certainty** |
| --- | --- | --- | --- | --- | --- | --- | --- | --- | --- | --- | --- | --- |
| **№ of studies** | **Study design** | **Risk of bias** | **Inconsistency** | **Indirectness** | **Imprecision** | **Other considerations** | | **Intervention** | **Comparator** | **Relative (95% CI)** | **Absolute (95% CI)** |  |
| **Allopurinol (vs. Placebo) - PEP** | | | | | | |  |  |  |  |  |  |
| 6 | randomised trials | serious: selection bias | serious: I squared 67.8 | not serious | not serious | none | | 83/986 (8.4%) | 96/988 (9.7%) | RR 0.77 (0.41 to 1.42) | **22 fewer per 1,000** (from 57 fewer to 41 more) | ⨁⨁◯◯ Low |
| **Allopurinol (vs. Placebo) - Mild PEP** | | | | | | |  | | | | | |
| 6 | randomised trials | serious: selection bias | not serious | not serious | serious: few participants | none | | 53/986 (5.4%) | 52/988 (5.3%) | RR 1.00 (0.58 to 1.72) | **0 fewer per 1,000** (from 22 fewer to 38 more) | ⨁⨁◯◯ Low |
| **Allopurinol (vs. Placebo) - Moderate PEP** | | | | | | |  | | | | | |
| 5 | randomised trials | serious: selection bias | not serious | not serious | serious: few participants | none | | 25/901 (2.8%) | 38/903 (4.2%) | RR 0.75 (0.36 to 1.57) | **11 fewer per 1,000** (from 27 fewer to 24 more) | ⨁⨁◯◯ Low |
| **Allopurinol (vs. Placebo) - Severe PEP** | | | | | | |  | | | | | |
| 4 | randomised trials | serious: selection bias | not serious | not serious | very serious: few participants, wide confidence intervals | none | | 5/872 (0.6%) | 6/858 (0.7%) | RR 0.90 (0.28 to 2.89) | **1 fewer per 1,000** (from 5 fewer to 13 more) | ⨁◯◯◯ Very low |
| **Epinephrine (vs. Placebo/No treatment) - Mild PEP** | | | | | | | | | | | | |
| 2 | randomised trials | not serious | not serious | not serious | serious: few participants | none | | 6/646 (0.9%) | 26/665 (3.9%) | RR 0.26 (0.11 to 0.60) | **29 fewer per 1,000** (from 35 fewer to 16 fewer) | ⨁⨁⨁◯ Moderate |
| **Epinephrine (vs. Placebo/No treatment) - Moderate to severe PEP** | | | | | | | | | | | | |
| 2 | randomised trials | not serious | not serious | not serious | serious: few participants | none | | 3/646 (0.5%) | 9/665 (1.4%) | RR 0.38 (0.11 to 1.29) | **8 fewer per 1,000** (from 12 fewer to 4 more) | ⨁⨁⨁◯ Moderate |
| **Epinephrine + NSAIDs (vs. NSAIDs) - Mild PEP** | | | | | | | | | | | | |
| 4 | randomised trials | serious: performance bias | not serious | not serious | serious: few participants | publication bias strongly suspected: Egger’s p value 0.04 | | 63/1346 (4.7%) | 51/1368 (3.7%) | RR 1.26 (0.82 to 1.94) | **10 more per 1,000** (from 7 fewer to 35 more) | ⨁◯◯◯ Very low |
| **Epinephrine + NSAIDs (vs. NSAIDs) - Moderate to severe PEP** | | | | | | | | | | | | |
| 4 | randomised trials | serious: performance bias | not serious | not serious | serious: few participants | none | | 24/1346 (1.8%) | 35/1368 (2.6%) | RR 0.73 (0.30 to 1.76) | **7 fewer per 1,000** (from 18 fewer to 19 more) | ⨁⨁◯◯ Low |
| **Aggressive hydration + rectal NSAIDs (vs. rectal NSAIDs) - PEP** | | | | | | | | | | | | |
| 3 | randomised trials | serious: performance bias | serious: I squared 61.5 | not serious | serious: few participants | none | | 33/537 (6.1%) | 56/573 (9.8%) | OR 0.43 (0.12 to 1.57) | **53 fewer per 1,000** (from 85 fewer to 48 more) | ⨁◯◯◯ Very low |
| **Aggressive hydration + rectal NSAIDs (vs. rectal NSAIDs) - Severe PEP** | | | | | | | | | | | | |
| 2 | randomised trials | serious: performance bias | not serious | not serious | serious: few participants | none | | 3/436 (0.7%) | 11/473 (2.3%) | OR 0.32 (0.10 to 1.08) | **16 fewer per 1,000** (from 21 fewer to 2 more) | ⨁⨁◯◯ Low |
| **Gabexate mesilate (vs. Placebo/No treatment) - PEP** | | | | | | | | | | | | |
| 12 | randomised trials | serious: selection bias, performance bias | not serious | not serious | not serious | publication bias strongly suspected: Egger’s p value 0.02 | | 72/1816 (4.0%) | 118/1846 (6.4%) | RR 0.56 (0.37 to 0.86) | **28 fewer per 1,000** (from 40 fewer to 9 fewer) | ⨁⨁◯◯ Low |
| **Glyceryl Trinitrate (vs. Placebo/Physiological saline/Vitamin C/No intervention) - PEP** | | | | | | | | | | | | |
| 11 | randomised trials | serious: selection bias | not serious | not serious | not serious | none | | 84/1189 (7.1%) | 127/1206 (10.5%) | RR 0.68 (0.51 to 0.90) | **34 fewer per 1,000** (from 52 fewer to 11 fewer) | ⨁⨁⨁◯ Moderate |
| **Glyceryl Trinitrate (vs. Placebo/Physiological saline/Vitamin C/No intervention) - Moderate to severe PEP** | | | | | | | | | | | | |
| 5 | randomised trials | serious: selection bias | not serious | not serious | serious: few participants | none | | 25/805 (3.1%) | 36/816 (4.4%) | RR 0.70 (0.42 to 1.15) | **13 fewer per 1,000** (from 26 fewer to 7 more) | ⨁⨁◯◯ Low |
| **Epinephrine + NSAIDs (vs. NSAIDs) - PEP** | | | | | | | | | | | | |
| 6 | randomised trials | serious: attrition bias | not serious | not serious | not serious | none | | 127/1987 (6.4%) | 126/2029 (6.2%) | OR 1.00 (0.68 to 1.45) | **0 fewer per 1,000** (from 19 fewer to 25 more) | ⨁⨁⨁◯ Moderate |
| **NSAIDs (vs. No NSAIDs) - Moderate to severe PEP** | | | | | | | | | | | | |
| 19 | randomised trials | serious: performance bias, detection bias | not serious | not serious | not serious | none | | 55/3176 (1.7%) | 98/3105 (3.2%) | OR 0.57 (0.40 to 0.81) | **13 fewer per 1,000** (from 19 fewer to 6 fewer) | ⨁⨁⨁◯ Moderate |
| **Indomethacin (vs. No NSAIDs) - PEP** | | | | | | | | | | | | |
| 10 | randomised trials | serious: performance bias, detection bias | not serious | not serious | not serious | none | | 130/2101 (6.2%) | 231/2063 (11.2%) | OR 0.52 (0.39 to 0.71) | **50 fewer per 1,000** (from 65 fewer to 30 fewer) | ⨁⨁⨁◯ Moderate |
| **Indomethacin (vs. No NSAIDs) - Moderate to severe PEP** | | | | | | | | | | | | |
| 8 | randomised trials | serious: performance bias, detection bias | not serious | not serious | serious: few participants | none | | 25/1825 (1.4%) | 53/1783 (3.0%) | OR 0.50 (0.30 to 0.81) | **15 fewer per 1,000** (from 21 fewer to 6 fewer) | ⨁⨁◯◯ Low |
| **Diclofenac (vs. No NSAIDs) - Moderate to severe PEP** | | | | | | | | | | | | |
| 10 | randomised trials | serious: performance bias, detection bias | not serious | not serious | serious: few participants | none | | 26/1189 (2.2%) | 35/1160 (3.0%) | OR 0.74 (0.43 to 1.26) | **8 fewer per 1,000** (from 17 fewer to 8 more) | ⨁⨁◯◯ Low |
| **Antioxidants (vs. Placebo/No intervention) - PEP** | | | | | | | | | | | | |
| 12 | randomised trials | very serious: no risk of bias assessment was reported. | serious: I squared 50.8 | not serious | not serious | none | | 132/1534 (8.6%) | 153/1576 (9.7%) | RR 0.84 (0.59 to 1.22) | **16 fewer per 1,000** (from 40 fewer to 21 more) | ⨁◯◯◯ Very low |
| **Antioxidants (vs. Placebo/No intervention) - Mild PEP** | | | | | | | | | | | | |
| 9 | randomised trials | very serious: no risk of bias assessment was reported. | not serious | not serious | serious: few participants | none | | 77/1409 (5.5%) | 72/1455 (4.9%) | RR 1.14 (0.82 to 1.57) | **7 more per 1,000** (from 9 fewer to 28 more) | ⨁◯◯◯ Very low |
| **Antioxidants (vs. Placebo/No intervention) - Moderate PEP** | | | | | | | | | | | | |
| 8 | randomised trials | very serious: no risk of bias assessment was reported. | not serious | not serious | serious: few participants | none | | 37/1324 (2.8%) | 47/1370 (3.4%) | RR 0.98 (0.63 to 1.53) | **1 fewer per 1,000** (from 13 fewer to 18 more) | ⨁◯◯◯ Very low |
| **Antioxidants (vs. Placebo/No intervention) - Severe PEP** | | | | | | | | | | | | |
| 6 | randomised trials | very serious: no risk of bias assessment was reported. | not serious | not serious | serious: few participants | none | | 7/1171 (0.6%) | 11/1200 (0.9%) | RR 0.84 (0.31 to 2.25) | **1 fewer per 1,000** (from 6 fewer to 11 more) | ⨁◯◯◯ Very low |
| **NSAIDs (vs. Placebo/Glycerin/No treatment) - Mild PEP** | | | | | | | | | | | | |
| 14 | randomised trials | serious: selection bias, attrition bias | not serious | not serious | not serious | none | | 129/3101 (4.2%) | 219/3107 (7.0%) | RR 0.60 (0.47 to 0.77) | **28 fewer per 1,000** (from 37 fewer to 16 fewer) | ⨁⨁⨁◯ Moderate |
| **Epinephrine (vs. Placebo/No treatment) - PEP** | | | | | | | | | | | | |
| 3 | randomised trials | serious: one RCT had a Jadad score below 3. | not serious | not serious | serious: few participants | none | | 13/720 (1.8%) | 42/736 (5.7%) | RR 0.34 (0.18 to 0.62) | **38 fewer per 1,000** (from 47 fewer to 22 fewer) | ⨁⨁◯◯ Low |
| **NSAIDs (vs. Placebo) - PEP in high-risk patients** | | | | | | | | | | | | |
| 6 | randomised trials | serious: performance bias, detection bias | serious: I squared 62.8 | not serious | not serious | none | | 64/711 (9.0%) | 120/728 (16.5%) | RR 0.54 (0.31 to 0.94) | **76 fewer per 1,000** (from 114 fewer to 10 fewer) | ⨁⨁◯◯ Low |
| **Diclofenac (vs. Placebo) - PEP in high-risk patients** | | | | | | | | | | | | |
| 4 | randomised trials | serious: performance bias, detection bias | serious: I squared 69.5 | not serious | serious: few participants | none | | 33/334 (9.9%) | 51/337 (15.1%) | RR 0.63 (0.27 to 1.50) | **56 fewer per 1,000** (from 110 fewer to 76 more) | ⨁◯◯◯ Very low |
| **Antibiotics (vs. Non-antibiotics) - PEP** | | | | | | | | | | | | |
| 4 | randomised trials | serious: detection bias | not serious | not serious | serious: few participants | none | | 13/321 (4.0%) | 30/377 (8.0%) | RR 0.51 (0.27 to 0.97) | **39 fewer per 1,000** (from 58 fewer to 2 fewer) | ⨁⨁◯◯ Low |
| **Nafamostat mesylate (vs. Placebo/No treatment) - PEP** | | | | | | | | | | | | |
| 8 | randomised trials | serious: attrition bias | not serious | not serious | not serious | none | | 81/1772 (4.6%) | 123/1438 (8.6%) | RR 0.50 (0.38 to 0.66) | **43 fewer per 1,000** (from 53 fewer to 29 fewer) | ⨁⨁⨁◯ Moderate |
| **Nafamostat mesylate (vs. Placebo/No treatment) - Mild PEP** | | | | | | | | | | | | |
| 7 | randomised trials | serious: selection bias | not serious | not serious | not serious | none | | 60/1730 (3.5%) | 90/1380 (6.5%) | RR 0.49 (0.36 to 0.69) | **33 fewer per 1,000** (from 42 fewer to 20 fewer) | ⨁⨁⨁◯ Moderate |
| **Nafamostat mesylate (vs. Placebo/No treatment) - Moderate to severe PEP** | | | | | | | | | | | | |
| 7 | randomised trials | serious: selection bias | not serious | not serious | serious: few participants | publication bias strongly suspected: Egger’s p value 0.06 | | 20/1730 (1.2%) | 29/1380 (2.1%) | RR 0.61 (0.33 to 1.15) | **8 fewer per 1,000** (from 14 fewer to 3 more) | ⨁◯◯◯ Very low |
| **NSAIDs (vs. Placebo SN/Placebo lactated Ringer’s solution/Ceftazidime/Glycerin/Glyceryl trinitrate/Saline solution /No treatment) - PEP** | | | | | | | | | | | | |
| 26 | randomised trials | not serious | serious: I squared 52.9 | not serious | not serious | none | | 298/4020 (7.4%) | 484/4123 (11.7%) | RR 0.65 (0.52 to 0.81) | **41 fewer per 1,000** (from 56 fewer to 22 fewer) | ⨁⨁⨁◯ Moderate |
| **NSAIDs (vs. Placebo SN/Placebo lactated Ringer’s solution/Ceftazidime/Glycerin/Glyceryl trinitrate/Saline solution /No treatment) - Moderate PEP** | | | | | | | | | | | | |
| 11 | randomised trials | not serious | not serious | not serious | serious: few participants | none | | 54/2134 (2.5%) | 89/2150 (4.1%) | RR 0.62 (0.44 to 0.89) | **16 fewer per 1,000** (from 23 fewer to 5 fewer) | ⨁⨁⨁◯ Moderate |
| **NSAIDs (vs. Placebo SN/Placebo lactated Ringer’s solution/Ceftazidime/Glycerin/Glyceryl trinitrate/Saline solution /No treatment) - Severe PEP** | | | | | | | | | | | | |
| 7 | randomised trials | not serious | not serious | not serious | serious: few participants | none | | 16/1740 (0.9%) | 23/1747 (1.3%) | RR 0.72 (0.38 to 1.38) | **4 fewer per 1,000** (from 8 fewer to 5 more) | ⨁⨁⨁◯ Moderate |
| **Diclofenac (vs. Placebo SN/Placebo lactated Ringer’s solution/Ceftazidime/Glycerin/Glyceryl trinitrate/Saline solution /No treatment) - PEP** | | | | | | | | | | | | |
| 14 | randomised trials | not serious | serious: I squared 59.6 | not serious | not serious | publication bias strongly suspected: Egger’s p value 0.08 | | 150/1709 (8.8%) | 229/1792 (12.8%) | RR 0.70 (0.49 to 0.98) | **38 fewer per 1,000** (from 65 fewer to 3 fewer) | ⨁⨁◯◯ Low |
| **Indomethacin (vs. Placebo) - Mild PEP** | | | | | | | | | | | | |
| 7 | randomised trials | serious: detection bias, reporting bias | serious: I squared 50.0 | not serious | not serious | none | | 75/1513 (5.0%) | 122/1500 (8.1%) | RR 0.61 (0.40 to 0.93) | **32 fewer per 1,000** (from 49 fewer to 6 fewer) | ⨁⨁◯◯ Low |
| **Indomethacin (vs. Placebo) - PEP in high-risk patients** | | | | | | | | | | | | |
| 3 | randomised trials | serious: reporting bias | not serious | not serious | not serious | publication bias strongly suspected: Egger’s p value 0.05 | | 45/577 (7.8%) | 101/584 (17.3%) | RR 0.46 (0.32 to 0.65) | **93 fewer per 1,000** (from 118 fewer to 61 fewer) | ⨁⨁◯◯ Low |
| **Somatostatin (vs. Placebo/No treatment) - PEP** | | | | | | | | | | | | |
| 14 | randomised trials | serious: the specific assessment results of each RCT across the bias dimensions were not individually disclosed. | serious: I squared 55.8 | not serious | not serious | none | | 142/2411 (5.9%) | 184/2182 (8.4%) | OR 0.60 (0.41 to 0.89) | **32 fewer per 1,000** (from 48 fewer to 9 fewer) | ⨁⨁◯◯ Low |
| **Somatostatin (vs. Placebo/No treatment) - PEP in high-risk patients** | | | | | | | | | | | | |
| 3 | randomised trials | serious: the specific assessment results of each RCT across the bias dimensions were not individually disclosed. | not serious | not serious | serious: few participants | none | | 59/628 (9.4%) | 54/486 (11.1%) | OR 0.62 (0.41 to 0.93) | **39 fewer per 1,000** (from 62 fewer to 7 fewer) | ⨁⨁◯◯ Low |
| **Aggressive hydration with Lactated Ringer Solution (vs. Standard hydration with Lactated Ringer Solution) - Moderate PEP** | | | | | | | | | | | | |
| 4 | randomised trials | serious: detection bias | serious: I squared 55.8 | not serious | serious: few participants, wide confidence intervals | none | | 6/448 (1.3%) | 11/447 (2.5%) | OR 0.66 (0.09 to 4.62) | **8 fewer per 1,000** (from 22 fewer to 80 more) | ⨁◯◯◯ Very low |
| **Aggressive hydration with Lactated Ringer Solution (vs. Standard hydration with Lactated Ringer Solution) - Moderate to severe PEP** | | | | | | | | | | | | |
| 3 | randomised trials | serious: attrition bias | not serious | not serious | not serious | none | | 43/369 (11.7%) | 83/353 (23.5%) | OR 0.38 (0.25 to 0.59) | **131 fewer per 1,000** (from 164 fewer to 82 fewer) | ⨁⨁⨁◯ Moderate |
| **Aggressive hydration with Lactated Ringer Solution (vs. Standard hydration with Lactated Ringer Solution) - PEP in high-risk patients** | | | | | | | | | | | | |
| 2 | randomised trials | serious: attrition bias | not serious | not serious | serious: few participants | none | | 3/97 (3.1%) | 9/85 (10.6%) | OR 0.31 (0.09 to 1.12) | **70 fewer per 1,000** (from 95 fewer to 11 more) | ⨁⨁◯◯ Low |
| **Aggressive hydration with Lactated Ringer Solution (vs. Standard hydration with Lactated Ringer Solution) - PEP** | | | | | | | | | | | | |
| 10 | randomised trials | serious: performance bias, attrition bias | not serious | not serious | not serious | none | | 55/1111 (5.0%) | 127/1089 (11.7%) | OR 0.40 (0.26 to 0.63) | **66 fewer per 1,000** (from 83 fewer to 40 fewer) | ⨁⨁⨁◯ Moderate |
| **Nafamostat mesylate (vs. Placebo/No treatment) - Moderate PEP** | | | | | | | | | | | | |
| 7 | randomised trials | serious: detection bias, reporting bias | not serious | not serious | serious: few participants, wide confidence intervals | none | | 15/1730 (0.9%) | 25/1380 (1.8%) | RR 0.54 (0.26 to 1.12) | **8 fewer per 1,000** (from 13 fewer to 2 more) | ⨁⨁◯◯ Low |
| **Nafamostat mesylate (vs. Placebo/No treatment) - Severe PEP** | | | | | | | | | | | | |
| 3 | randomised trials | serious: detection bias, reporting bias | not serious | not serious | very serious: few participants, wide confidence intervals | none | | 5/785 (0.6%) | 4/634 (0.6%) | RR 0.92 (0.24 to 3.50) | **1 fewer per 1,000** (from 5 fewer to 16 more) | ⨁◯◯◯ Very low |
| **Nafamostat mesylate (vs. Placebo/No treatment) - PEP in high-risk patients** | | | | | | | | | | | | |
| 6 | randomised trials | serious: detection bias, reporting bias | not serious | not serious | serious: few participants | none | | 56/760 (7.4%) | 60/534 (11.2%) | RR 0.57 (0.35 to 0.94) | **48 fewer per 1,000** (from 73 fewer to 7 fewer) | ⨁⨁◯◯ Low |
| **Ulinastatin (vs. Placebo) - PEP** | | | | | | | | | | | | |
| 6 | randomised trials | very serious: four RCTs had a Jadad score below 3. | not serious | not serious | not serious | none | | 39/752 (5.2%) | 89/1174 (7.6%) | RR 0.71 (0.48 to 1.05) | **22 fewer per 1,000** (from 39 fewer to 4 more) | ⨁⨁◯◯ Low |
| **Glyceryl Trinitrate + NSAIDs (vs. NSAIDs) - PEP** | | | | | | | | | | | | |
| 3 | randomised trials | serious: selection bias | not serious | not serious | serious: few participants | none | | 35/629 (5.6%) | 67/625 (10.7%) | RR 0.53 (0.36 to 0.78) | **50 fewer per 1,000** (from 69 fewer to 24 fewer) | ⨁⨁◯◯ Low |
| **Glyceryl Trinitrate + NSAIDs (vs. NSAIDs) - Non-mild PEP** | | | | | | | | | | | | |
| 2 | randomised trials | serious: selection bias | not serious | not serious | serious: few participants | none | | 6/594 (1.0%) | 14/592 (2.4%) | RR 0.43 (0.17 to 1.11) | **13 fewer per 1,000** (from 20 fewer to 3 more) | ⨁⨁◯◯ Low |
| **Octreotide (vs. Placebo) - PEP** | | | | | | | | | | | | |
| 16 | randomised trials | serious: performance bias, detection bias | not serious | not serious | not serious | none | | 73/1351 (5.4%) | 95/1370 (6.9%) | OR 0.81 (0.54 to 1.23) | **12 fewer per 1,000** (from 31 fewer to 15 more) | ⨁⨁⨁◯ Moderate |
| **Corticosteroid (vs. Placebo) - PEP** | | | | | | | | | | | | |
| 7 | randomised trials | serious: two RCTs had a Jadad score below 3. | not serious | not serious | not serious | none | | 157/1312 (12.0%) | 142/1320 (10.8%) | OR 1.10 (0.77 to 1.55) | **9 more per 1,000** (from 23 fewer to 50 more) | ⨁⨁⨁◯ Moderate |
| **Corticosteroid (vs. Placebo) - Severe PEP** | | | | | | | | | | | | |
| 4 | randomised trials | serious: two RCTs had a Jadad score below 3. | not serious | not serious | serious: few participants, wide confidence intervals | publication bias strongly suspected: Egger’s p value 0.08 | | 16/1009 (1.6%) | 10/1020 (1.0%) | OR 1.50 (0.67 to 3.36) | **5 more per 1,000** (from 3 fewer to 22 more) | ⨁◯◯◯ Very low |
| **Gabexate mesilate (vs. Placebo/No treatment) - Severe PEP** | | | | | | | | | | | | |
| 4 | randomised trials | serious: one RCT had a Jadad score below 3. | not serious | not serious | serious: few participants, wide confidence intervals | publication bias strongly suspected: Egger’s p value 0.03 | | 7/1203 (0.6%) | 3/1013 (0.3%) | OR 1.40 (0.34 to 5.73) | **1 more per 1,000** (from 2 fewer to 14 more) | ⨁◯◯◯ Very low |
| **0.035-inch guidewire (vs. 0.025-inch guidewire) - PEP** | | | | | | | | | | | | |
| 3 | randomised trials | serious: performance bias | not serious | not serious | serious: few participants | none | | 36/545 (6.6%) | 32/516 (6.2%) | RR 1.15 (0.72 to 1.81) | **9 more per 1,000** (from 17 fewer to 50 more) | ⨁⨁◯◯ Low |
| **Endocut (vs. Pure cut) - PEP** | | | | | | | | | | | | |
| 4 | randomised trials | serious: selection bias | not serious | not serious | serious: few participants, wide confidence intervals | none | | 28/507 (5.5%) | 8/480 (1.7%) | RR 3.00 (1.40 to 6.45) | **33 more per 1,000** (from 7 more to 91 more) | ⨁⨁◯◯ Low |
| **Endocut (vs. Pure cut) - Mild PEP** | | | | | | | | | | | | |
| 4 | randomised trials | serious: selection bias | not serious | not serious | serious: few participants, wide confidence intervals | none | | 24/507 (4.7%) | 9/480 (1.9%) | RR 2.53 (1.24 to 5.16) | **29 more per 1,000** (from 5 more to 78 more) | ⨁⨁◯◯ Low |
| **Endocut (vs. Pure cut) - Moderate PEP** | | | | | | | | | | | | |
| 3 | randomised trials | serious: selection bias | not serious | not serious | very serious: few participants, wide confidence intervals | none | | 7/466 (1.5%) | 1/437 (0.2%) | RR 3.22 (0.68 to 15.33) | **5 more per 1,000** (from 1 fewer to 33 more) | ⨁◯◯◯ Very low |
| **Endocut (vs. Pure cut) - Severe PEP** | | | | | | | | | | | | |
| 2 | randomised trials | serious: selection bias | not serious | not serious | very serious: few participants, wide confidence intervals | none | | 5/96 (5.2%) | 1/74 (1.4%) | RR 3.16 (0.53 to 18.71) | **29 more per 1,000** (from 6 fewer to 239 more) | ⨁◯◯◯ Very low |
| **Early precut sphincterotomy (vs. Persistent cannulation attempts) - PEP** | | | | | | | | | | | | |
| 7 | randomised trials | serious: two RCTs had a Jadad score below 3. | not serious | not serious | serious: few participants | none | | 19/478 (4.0%) | 34/554 (6.1%) | OR 0.63 (0.34 to 1.18) | **22 fewer per 1,000** (from 40 fewer to 10 more) | ⨁⨁◯◯ Low |
| **Pancreatic stent placement (vs. No Pancreatic stent placement) - Mild PEP in high-risk patients** | | | | | | | | | | | | |
| 9 | randomised trials | serious: three RCTs had a Jadad score below 3. | not serious | not serious | not serious | none | | 30/617 (4.9%) | 70/632 (11.1%) | RR 0.48 (0.32 to 0.73) | **58 fewer per 1,000** (from 75 fewer to 30 fewer) | ⨁⨁⨁◯ Moderate |
| **Pancreatic stent placement (vs. No Pancreatic stent placement) - Moderate PEP in high-risk patients** | | | | | | | | | | | | |
| 8 | randomised trials | serious: three RCTs had a Jadad score below 3. | not serious | not serious | serious: few participants | none | | 8/557 (1.4%) | 28/572 (4.9%) | RR 0.35 (0.17 to 0.72) | **32 fewer per 1,000** (from 41 fewer to 14 fewer) | ⨁⨁◯◯ Low |
| **Pancreatic stent placement (vs. No Pancreatic stent placement) - Severe PEP in high-risk patients** | | | | | | | | | | | | |
| 7 | randomised trials | serious: three RCTs had a Jadad score below 3. | not serious | not serious | serious: few participants | none | | 1/463 (0.2%) | 12/469 (2.6%) | RR 0.31 (0.10 to 0.97) | **18 fewer per 1,000** (from 23 fewer to 1 fewer) | ⨁⨁◯◯ Low |
| **Endoscopic sphincterotomy associated with balloon dilation (vs. Endoscopic sphincterotomy) - PEP** | | | | | | | | | | | | |
| 9 | randomised trials | serious: detection bias | not serious | not serious | not serious | none | | 40/851 (4.7%) | 48/831 (5.8%) | RR 0.82 (0.55 to 1.24) | **10 fewer per 1,000** (from 26 fewer to 14 more) | ⨁⨁⨁◯ Moderate |
| **Pancreatic stent placement (vs. No Pancreatic stent placement) - Mild PEP** | | | | | | | | | | | | |
| 13 | randomised trials | serious: performance bias, detection bias | not serious | not serious | not serious | publication bias strongly suspected: Egger’s p value 0.004 | | 49/740 (6.6%) | 120/761 (15.8%) | OR 0.39 (0.27 to 0.56) | **90 fewer per 1,000** (from 110 fewer to 63 fewer) | ⨁⨁◯◯ Low |
| **Pancreatic stent placement (vs. No Pancreatic stent placement) - Severe PEP** | | | | | | | | | | | | |
| 8 | randomised trials | serious: performance bias, detection bias | not serious | not serious | not serious | publication bias strongly suspected: Egger’s p value ＜0.001 | | 0/493 (0.0%) | 13/516 (2.5%) | OR 0.25 (0.08 to 0.75) | **19 fewer per 1,000** (from 23 fewer to 6 fewer) | ⨁⨁◯◯ Low |
| **Endocut (vs. Blend) - Mild PEP** | | | | | | | | | | | | |
| 2 | randomised trials | serious: performance bias | not serious | not serious | very serious: wide confidence intervals | none | | 9/229 (3.9%) | 8/231 (3.5%) | RR 1.32 (0.26 to 6.63) | **11 more per 1,000** (from 26 fewer to 195 more) | ⨁◯◯◯ Very low |
| **Endocut (vs. Blend) - Moderate PEP** | | | | | | | | | | | | |
| 1 | randomised trials | serious: performance bias | not serious | not serious | very serious: wide confidence intervals | none | | 1/179 (0.6%) | 0/181 (0.0%) | RR 3.03 (0.12 to 73.97) | **0 fewer per 1,000** (from 0 fewer to 0 fewer) | ⨁◯◯◯ Very lo |
| **Endocut (vs. Blend) - PEP** | | | | | | | | | | | | |
| 3 | randomised trials | serious: performance bias, detection bias | not serious | not serious | serious: few participants | none | | 7/284 (2.5%) | 12/310 (3.9%) | RR 0.64 (0.25 to 1.64) | **14 fewer per 1,000** (from 29 fewer to 25 more) | ⨁⨁◯◯ Low |
| **Endoscopic papillary large balloon dilation (vs. Endoscopic papillary large balloon dilation + Endoscopic sphincterotomy) - PEP** | | | | | | | | | | | | |
| 7 | randomised trials | not serious | not serious | not serious | serious: few participants | none | | 19/369 (5.1%) | 18/367 (4.9%) | OR 0.88 (0.43 to 1.78) | **6 fewer per 1,000** (from 27 fewer to 35 more) | ⨁⨁⨁◯ Moderate |
| **Needle-knife precut (vs. Transpancreatic sphincterotomy) - PEP in patients with difficult cannulation** | | | | | | | | | | | | |
| 3 | randomised trials | not serious | not serious | not serious | very serious: few participants, wide confidence intervals | none | | 10/136 (7.4%) | 4/128 (3.1%) | OR 2.42 (0.72 to 8.20) | **41 more per 1,000** (from 9 fewer to 178 more) | ⨁⨁◯◯ Low |
| **Endoscopic sphincterotomy (vs. Non-Endoscopic sphincterotomy) - PEP** | | | | | | | | | | | | |
| 6 | randomised trials | not serious | serious: I squared 59.2 | not serious | not serious | none | | 26/474 (5.5%) | 65/464 (14.0%) | OR 0.46 (0.19 to 1.09) | **70 fewer per 1,000** (from 110 fewer to 11 more) | ⨁⨁⨁◯ Moderate |
| **Endoscopic papillary balloon dilation (vs. Endoscopic sphincterotomy) - Severe PEP** | | | | | | | | | | | | |
| 6 | randomised trials | serious: reporting bias | not serious | not serious | serious: wide confidence intervals | none | | 14/513 (2.7%) | 2/506 (0.4%) | RR 3.32 (1.07 to 10.29) | **9 more per 1,000** (from 0 fewer to 37 more) | ⨁⨁◯◯ Low |
| **Pancreatic stent placement (vs. No Pancreatic stent placement) - Mild to Moderate PEP** | | | | | | | | | | | | |
| 13 | randomised trials | not serious | not serious | not serious | not serious | publication bias strongly suspected: Egger’s p value 0.003 | | 49/740 (6.6%) | 120/761 (15.8%) | RR 0.45 (0.32 to 0.62) | **87 fewer per 1,000** (from 107 fewer to 60 fewer) | ⨁⨁⨁◯ Moderate |
| **Pancreatic stent placement (vs. No Pancreatic stent placement) - PEP in high-risk patients** | | | | | | | | | | | | |
| 12 | randomised trials | not serious | not serious | not serious | not serious | publication bias strongly suspected: Egger’s p value ＜0.001 | | 49/630 (7.8%) | 130/646 (20.1%) | RR 0.41 (0.30 to 0.56) | **119 fewer per 1,000** (from 141 fewer to 89 fewer) | ⨁⨁⨁◯ Moderate |
| **Needle knife fistulotomy (vs. Conventional cannulation technique) - PEP** | | | | | | | | | | | | |
| 3 | randomised trials | serious: selection bias | not serious | not serious | serious: few participants | none | | 3/253 (1.2%) | 16/238 (6.7%) | OR 0.22 (0.04 to 1.04) | **52 fewer per 1,000** (from 64 fewer to 3 more) | ⨁⨁◯◯ Low |
| **Early precut sphincterotomy (vs. Persistent cannulation attempts) - PEP in patients with difficult biliary access** | | | | | | | | | | | | |
| 7 | randomised trials | serious: other bias | not serious | not serious | serious: few participants | none | | 26/462 (5.6%) | 52/537 (9.7%) | RR 0.57 (0.36 to 0.92) | **42 fewer per 1,000** (from 62 fewer to 8 fewer) | ⨁⨁◯◯ Low |
| **Endoscopic papillary balloon dilation (vs. Endoscopic sphincterotomy) - PEP** | | | | | | | | | | | | |
| 21 | randomised trials | serious: selection bias | not serious | not serious | not serious | none | | 105/1369 (7.7%) | 72/1395 (5.2%) | OR 1.36 (0.89 to 2.07) | **17 more per 1,000** (from 5 fewer to 50 more) | ⨁⨁⨁◯ Moderate |
| **Double-guidewire technique (vs. Other methods) - Mild PEP in patients with difficult biliary cannulation** | | | | | | | | | | | | |
| 5 | randomised trials | serious: performance bias | not serious | not serious | serious: few participants, wide confidence intervals | none | | 33/231 (14.3%) | 14/229 (6.1%) | RR 2.17 (1.00 to 4.72) | **72 more per 1,000** (from 0 fewer to 227 more) | ⨁⨁◯◯ Low |
| **Double-guidewire technique (vs. Other methods) - Moderate PEP in patients with difficult biliary cannulation** | | | | | | | | | | | | |
| 3 | randomised trials | serious: performance bias | not serious | not serious | serious: few participants, wide confidence intervals | none | | 5/154 (3.2%) | 5/149 (3.4%) | RR 0.95 (0.27 to 3.38) | **2 fewer per 1,000** (from 24 fewer to 80 more) | ⨁⨁◯◯ Low |
| **Double-guidewire technique (vs. Other methods) - Severe PEP in patients with difficult biliary cannulation** | | | | | | | | | | | | |
| 1 | randomised trials | serious: performance bias | not serious | not serious | very serious: few participants, wide confidence intervals | none | | 2/97 (2.1%) | 1/91 (1.1%) | RR 1.88 (0.17 to 20.34) | **10 more per 1,000** (from 9 fewer to 213 more) | ⨁◯◯◯ Very lo |
| **Guidewire-assisted cannulation (vs. Contrast-assisted cannulation) - PEP** | | | | | | | | | | | | |
| 15 | randomised trials | serious: performance bias | not serious | not serious | not serious | publication bias strongly suspected: Egger’s p value 0.04 | | 88/2351 (3.7%) | 159/2075 (7.7%) | RR 0.51 (0.36 to 0.72) | **38 fewer per 1,000** (from 49 fewer to 21 fewer) | ⨁⨁◯◯ Low |
| **Guidewire-assisted cannulation (vs. Contrast-assisted cannulation) - Mild PEP** | | | | | | | | | | | | |
| 11 | randomised trials | serious: performance bias | not serious | not serious | not serious | publication bias strongly suspected: Egger’s p value 0.08 | | 43/1657 (2.6%) | 93/1649 (5.6%) | RR 0.47 (0.26 to 0.83) | **30 fewer per 1,000** (from 42 fewer to 10 fewer) | ⨁⨁◯◯ Low |
| **Guidewire-assisted cannulation (vs. Contrast-assisted cannulation) - Moderate PEP** | | | | | | | | | | | | |
| 9 | randomised trials | serious: performance bias | not serious | not serious | serious: few participants | publication bias strongly suspected: Egger’s p value 0.04 | | 14/1440 (1.0%) | 19/1436 (1.3%) | RR 0.76 (0.38 to 1.52) | **3 fewer per 1,000** (from 8 fewer to 7 more) | ⨁◯◯◯ Very low |
| **Guidewire-assisted cannulation (vs. Contrast-assisted cannulation) - Severe PEP** | | | | | | | | | | | | |
| 9 | randomised trials | serious: performance bias | not serious | not serious | serious: few participants | publication bias strongly suspected: Egger’s p value 0.09 | | 7/1567 (0.4%) | 11/1392 (0.8%) | RR 0.69 (0.27 to 1.81) | **2 fewer per 1,000** (from 6 fewer to 6 more) | ⨁◯◯◯ Very low |
| **Pure cut (vs. Blend) - PEP** | | | | | | | | | | | | |
| 4 | randomised trials | serious: the details of the CONSORT score were not reported. | not serious | not serious | serious: few participants | none | | 15/396 (3.8%) | 30/408 (7.4%) | RR 0.47 (0.18 to 1.20) | **39 fewer per 1,000** (from 60 fewer to 15 more) | ⨁⨁◯◯ Lo |
| **Pure cut (vs. Blend) - Mild PEP** | | | | | | | | | | | | |
| 4 | randomised trials | serious: the details of the CONSORT score were not reported. | not serious | not serious | serious: few participants | none | | 11/396 (2.8%) | 22/408 (5.4%) | RR 0.54 (0.26 to 1.12) | **25 fewer per 1,000** (from 40 fewer to 6 more) | ⨁⨁◯◯ Low |
| **Pure cut (vs. Blend) - Moderate PEP** | | | | | | | | | | | | |
| 3 | randomised trials | serious: the details of the CONSORT score were not reported. | not serious | not serious | serious: few participants, wide confidence intervals | publication bias strongly suspected: Egger’s p value 0.004 | | 2/336 (0.6%) | 7/347 (2.0%) | RR 0.43 (0.11 to 1.70) | **11 fewer per 1,000** (from 18 fewer to 14 more) | ⨁◯◯◯ Very low |
| **Pure cut (Blend) - Severe PEP** | | | | | | | | | | | | |
| 2 | randomised trials | serious: the details of the CONSORT score were not reported. | not serious | not serious | very serious: few participants, wide confidence intervals | none | | 2/202 (1.0%) | 1/214 (0.5%) | RR 1.41 (0.09 to 23.03) | **2 more per 1,000** (from 4 fewer to 103 more) | ⨁◯◯◯ Very low |
| **Double-guidewire technique (vs. Other methods) - PEP in patients with difficult biliary cannulation** | | | | | | | | | | | | |
| 19 | randomised trials | serious: performance bias, detection bias | not serious | not serious | not serious | none | | 111/973 (11.4%) | 103/982 (10.5%) | RR 0.99 (0.64 to 1.53) | **1 fewer per 1,000** (from 38 fewer to 56 more) | ⨁⨁⨁◯ Moderate |
| **Covered self-expandable metallic stents (vs. Multiple plastic stents) - PEP** | | | | | | | | | | | | |
| 4 | randomised trials | not serious | not serious | not serious | very serious: few participants, wide confidence intervals | none | | 14/418 (3.3%) | 9/736 (1.2%) | OR 3.17 (1.18 to 8.52) | **26 more per 1,000** (from 2 more to 83 more) | ⨁⨁◯◯ Low |
| **Pancreatic stent placement (vs. No Pancreatic stent placement) - PEP** | | | | | | | | | | | | |
| 14 | randomised trials | not serious | not serious | not serious | not serious | publication bias strongly suspected: Egger’s p value 0.001 | | 46/714 (6.4%) | 141/755 (18.7%) | RR 0.37 (0.27 to 0.51) | **118 fewer per 1,000** (from 136 fewer to 92 fewer) | ⨁⨁⨁◯ Moderate |
| **Pancreatic stent placement (vs. No Pancreatic stent placement) - Moderate PEP** | | | | | | | | | | | | |
| 8 | randomised trials | not serious | not serious | not serious | not serious | publication bias strongly suspected: Egger’s p value 0.08 | | 7/521 (1.3%) | 26/566 (4.6%) | RR 0.37 (0.17 to 0.79) | **29 fewer per 1,000** (from 38 fewer to 10 fewer) | ⨁⨁⨁◯ Moderate |

**CI:** confidence interval; **OR:** odds ratio; **RR:** risk ratio

**Table S7.** Re-evaluation of the evidence credibility for the association between pancreatic stent placement and the incidence of PEP using a meta-analysis with higher methodological quality (Mazaki et al., 2014).

| **Author, year** | **Intervention** | **Comparator** | **Outcome** | **Studies (Participants)** | **Effect metrics** | **Random effects summary estimate (95% Cl)** | **Random effects**  **p value** | **I^2^ (%)** | **95% prediction interval** | **Random effects estimate of the largest study (95% Cl)** | **Egger's test**  **p value** | **Signifcant studies** | | | **AMSTAR2** |
| --- | --- | --- | --- | --- | --- | --- | --- | --- | --- | --- | --- | --- | --- | --- | --- |
|  |  |  |  |  |  |  |  |  |  |  |  | **Observed** | **Expected** | **p value** |  |
| **Highly suggestive (Class II)** | | | | | | | | | | | | | | | |
| Mazaki, T. et al, 2014 | Pancreatic stent placement | No Pancreatic stent placement | PEP | 14 (1511) | RR | 0.37 (0.27-0.50) | 2.2 x 10^-10^ | 0% | 0.26-0.52 | 0.52 (0.29-0.92) | 0.003 | 7 | 5.71 | 0.59 | Low |

**Table S8.** Subgroup Analysis of timing, route, dose, regimen, and operator expertise in highly suggestive and suggestive evidence.

| **Intervention** | **Studies groups** | **Author, year** | **Comparator** | **Outcome** | **Studies** | **Participants** | **Random effects model** | | | **Heterogeneity** | |
| --- | --- | --- | --- | --- | --- | --- | --- | --- | --- | --- | --- |
|  |  |  |  |  |  |  | **Effect metrics** | **Summary effects (95%Cl)** | **p value** | **I^2^ (%)** | **p value** |
| **Group by timing** | | | | | | | | | | | |
| NSAIDs | Before ERCP | Serrano, J. P. R. et al, 2020 | Placebo SN/Placebo lactated Ringer’s solution/Ceftazidime/Glycerin/Glyceryl trinitrate/Saline solution /No treatment | PEP | 13 | 3098 | RR | 0.55 (0.38-0.79) | 1.0 x 10^-3^ | 60.2% | 0.003 |
|  | After ERCP |  |  |  | 10 | 3959 | RR | 0.64 (0.48-0.86) | 2.9 x 10^-3^ | 36.2% | 0.118 |
|  | Before and After ERCP |  |  |  | 2 | 637 | RR | 1.01 (0.66-1.55) | 9.8 x 10^-1^ | 0% | 0.871 |
|  | During ERCP |  |  |  | 1 | 449 | RR | 1.47 (0.70-3.11) | 3.1 x 10^-1^ | NA | NA |
| Indomethacin | Before ERCP | Lyu, Y. et al, 2018 | Placebo | PEP | 6 | 2229 | RR | 0.60 (0.45-0.80) | 5.2 x 10^-4^ | 0% | 0.594 |
|  | After ERCP |  |  |  | 2 | 768 | RR | 0.42 (0.20-0.88) | 2.1 x 10^-2^ | 49.2% | 0.161 |
|  | During ERCP |  |  |  | 1 | 449 | RR | 1.47 (0.70-3.11) | 3.1 x 10^-1^ | NA | NA |
| Diclofenac | Before ERCP | Lyu, Y. et al, 2018 | Placebo | PEP | 4 | 486 | RR | 0.32 (0.16-0.63) | 9.0 x 10^-4^ | 0% | 0.449 |
|  | After ERCP |  |  |  | 4 | 807 | RR | 0.65 (0.27-1.59) | 3.3 x 10^-1^ | 72.1% | 0.013 |
|  | Before and After ERCP |  |  |  | 1 | 207 | RR | 0.97 (0.53-1.80) | 9.2 x 10^-1^ | NA | NA |
| **Group by route** | | | | | | | | | | | |
| NSAIDs | Rectal | Serrano, J. P. R. et al, 2020 | Placebo SN/Placebo lactated Ringer’s solution/Ceftazidime/Glycerin/Glyceryl trinitrate/Saline solution /No treatment | PEP | 19 | 6107 | RR | 0.57 (0.44-0.75) | 4.9 x 10^-5^ | 56.4% | 0.001 |
|  | Intravenous |  |  |  | 3 | 811 | RR | 0.86 (0.49-1.54) | 6.2 x 10^-1^ | 0% | 0.477 |
|  | Oral |  |  |  | 3 | 807 | RR | 0.94 (0.65-1.37) | 7.6 x 10^-1^ | 0% | 0.824 |
|  | Intramuscular | Lyu, Y. et al, 2018 | Placebo |  | 3 | 625 | RR | 0.62 (0.25-1.52) | 2.8 x 10^-1^ | 57.3% | 0.096 |
| Diclofenac | Rectal | Lyu, Y. et al, 2018 | Placebo | PEP | 5 | 668 | RR | 0.37 (0.14-0.94) | 3.3 x 10^-2^ | 60.7% | 0.038 |
|  | Intramuscular |  |  |  | 3 | 625 | RR | 0.62 (0.25-1.52) | 2.8 x 10^-1^ | 57.3% | 0.096 |
|  | Oral |  |  |  | 1 | 207 | RR | 0.97 (0.53-1.80) | 9.2 x 10^-1^ | NA | NA |
| **Group by dose** | | | | | | | | | | | |
| Nafamostat mesylate | 20mg | Narumi, K. et al, 2023 | Placebo/No treatment | PEP | 5 | 2458 | RR | 0.50 (0.35-0.73) | 2.8 x 10^-4^ | 20.0% | 0.287 |
|  | 50mg |  |  |  | 4 | 952 | RR | 0.43 (0.26-0.72) | 1.6 x 10^-3^ | 0% | 0.699 |
| **Group by regimen** | | | | | | | | | | | |
| Aggressive hydration with Lactated Ringer Solution | Regimen A | Wang, R. C. et al, 2021 | Standard hydration  with Lactated Ringer Solution | PEP | 4 | 537 | RR | 0.21 (0.10-0.43) | 3.0 x 10^-5^ | 0% | 0.660 |
|  | Regimen B |  |  |  | 2 | 570 | RR | 0.62 (0.21-1.85) | 4.0 x 10^-1^ | 40.9% | 0.194 |
|  | Regimen C |  |  |  | 1 | 120 | RR | 1.26 (0.62-2.59) | 5.3 x 10^-1^ | NA | NA |
| **Group by operator expertise** | | | | | | | | | | | |
| Guidewire-assisted cannulation | ERCP performed by experienced endoscopists | Tse, F. et al, 2022 | Contrast-assisted cannulation | PEP | 6 | 1782 | RR | 0.34 (0.20-0.58) | 3.7 x 10^-5^ | 23.3% | 0.259 |
|  | ERCP performed by trainees first then by experienced endoscopists (trainee involvement) |  |  |  | 5 | 1700 | RR | 0.93 (0.60-1.46) | 7.4 x 10^-1^ | 5.0% | 0.378 |
|  | No information provided about trainee involvement |  |  |  | 4 | 944 | RR | 0.44 (0.26-0.73) | 1.5 x 10^-3^ | 0% | 0.864 |

NA=not available.

NP=not pertinent (because the number of expecied significan studies was larger than the number of observed significant studies).

**Figures S1-S91.** Forest plot of 91 associations between pharmacological interventions or procedural technical modifications and the incidence of PEP.

**Fig. S1.** Forest plot demonstrating the effects of allopurinol on the incidence of PEP.

**Fig. S2.** Forest plot demonstrating the effects of allopurinol on the incidence of mild PEP.

**Fig. S3.** Forest plot demonstrating the effects of allopurinol on the incidence of moderate PEP.

**Fig. S4.** Forest plot demonstrating the effects of allopurinol on the incidence of severe PEP.

**Fig. S5.** Forest plot demonstrating the effects of epinephrine monotherapy on the incidence of mild PEP.

**Fig. S6.** Forest plot demonstrating the effects of epinephrine monotherapy on the incidence of moderate to severe PEP.

**Fig. S7.** Forest plot demonstrating the effects of the combination of epinephrine and NSAIDs versus the use of NSAIDs alone on the incidence of mild PEP.

**Fig. S8.** Forest plot demonstrating the effects of the combination of epinephrine and NSAIDs versus the use of NSAIDs alone on the incidence of moderate to severe PEP.

**Fig. S9.** Forest plot demonstrating the effects of the combination of aggressive hydration and rectal NSAIDs versus the use of rectal NSAIDs alone on the incidence of PEP.

**Fig. S10.** Forest plot demonstrating the effects of the combination of aggressive hydration and rectal NSAIDs versus the use of rectal NSAIDs alone on the incidence of severe PEP.

**Fig. S11.** Forest plot demonstrating the effects of gabexate mesilate on the incidence of PEP.

**Fig. S12.** Forest plot demonstrating the effects of glyceryl trinitrate on the incidence of PEP.

**Fig. S13.** Forest plot demonstrating the effects of glyceryl trinitrate on the incidence of moderate to severe PEP.

**Fig. S14.** Forest plot demonstrating the effects of the combination of epinephrine and NSAIDs versus the use of NSAIDs alone on the incidence of PEP.

**Fig. S15.** Forest plot demonstrating the effects of NSAIDs on the incidence of moderate to severe PEP.

**Fig. S16.** Forest plot demonstrating the effects of indomethacin on the incidence of PEP.

**Fig. S17.** Forest plot demonstrating the effects of indomethacin on the incidence of moderate to severe PEP.

**Fig. S18.** Forest plot demonstrating the effects of diclofenac on the incidence of moderate to severe PEP.

**Fig. S19.** Forest plot demonstrating the effects of antioxidants on the incidence of PEP.

**Fig. S20.** Forest plot demonstrating the effects of antioxidants on the incidence of mild PEP.

**Fig. S21.** Forest plot demonstrating the effects of antioxidants on the incidence of moderate PEP.

**Fig. S22.** Forest plot demonstrating the effects of antioxidants on the incidence of severe PEP.

**Fig. S23.** Forest plot demonstrating the effects of NSAIDs on the incidence of mild PEP.

**Fig. S24.** Forest plot demonstrating the effects of epinephrine monotherapy on the incidence of PEP.

**Fig. S25.** Forest plot demonstrating the effects of NSAIDs on the incidence of PEP in high-risk patients.

**Fig. S26.** Forest plot demonstrating the effects of diclofenac on the incidence of PEP in high-risk patients.

**Fig. S27.** Forest plot demonstrating the effects of antibiotics on the incidence of PEP.

**Fig. S28.** Forest plot demonstrating the effects of nafamostat mesylate on the incidence of PEP.

**Fig. S29.** Forest plot demonstrating the effects of nafamostat mesylate on the incidence of mild PEP.

**Fig. S30.** Forest plot demonstrating the effects of nafamostat mesylate on the incidence of moderate to severe PEP.

**Fig. S31.** Forest plot demonstrating the effects of NSAIDs on the incidence of PEP.

**Fig. S32.** Forest plot demonstrating the effects of NSAIDs on the incidence of moderate PEP.

**Fig. S33.** Forest plot demonstrating the effects of NSAIDs on the incidence of severe PEP.

**Fig. S34.** Forest plot demonstrating the effects of diclofenac on the incidence of PEP.

**Fig. S35.** Forest plot demonstrating the effects of indomethacin on the incidence of mild PEP.

**Fig. S36.** Forest plot demonstrating the effects of indomethacin on the incidence of PEP in high-risk patients.

**Fig. S37.** Forest plot demonstrating the effects of somatostatin on the incidence of PEP.

**Fig. S38.** Forest plot demonstrating the effects of somatostatin on the incidence of PEP in high-risk patients.

**Fig. S39.** Forest plot demonstrating the effects of aggressive hydration with Lactated Ringer Solution versus standard hydration with Lactated Ringer Solution on the incidence of moderate PEP.

**Fig. S40.** Forest plot demonstrating the effects of aggressive hydration with Lactated Ringer Solution versus standard hydration with Lactated Ringer Solution on the incidence of moderate to severe PEP.

**Fig. S41.** Forest plot demonstrating the effects of aggressive hydration with Lactated Ringer Solution versus standard hydration with Lactated Ringer Solution on the incidence of PEP in high-risk patients.

**Fig. S42.** Forest plot demonstrating the effects of aggressive hydration with Lactated Ringer Solution versus standard hydration with Lactated Ringer Solution on the incidence of PEP.

**Fig. S43.** Forest plot demonstrating the effects of nafamostat mesylate on the incidence of moderate PEP.

**Fig. S44.** Forest plot demonstrating the effects of nafamostat mesylate on the incidence of severe PEP.

**Fig. S45.** Forest plot demonstrating the effects of nafamostat mesylate on the incidence of PEP in high-risk patients.

**Fig. S46.** Forest plot demonstrating the effects of ulinastatin on the incidence of PEP.

**Fig. S47.** Forest plot demonstrating the effects of the combination of glyceryl trinitrate and NSAIDs versus the use of NSAIDs alone on the incidence of PEP.

**Fig. S48.** Forest plot demonstrating the effects of the combination of glyceryl trinitrate and NSAIDs versus the use of NSAIDs alone on the incidence of non-mild PEP.

**Fig. S49.** Forest plot demonstrating the effects of octreotide on the incidence of PEP.

**Fig. S50.** Forest plot demonstrating the effects of corticosteroid on the incidence of PEP.

**Fig. S51.** Forest plot demonstrating the effects of corticosteroid on the incidence of severe PEP.

**Fig. S52.** Forest plot demonstrating the effects of gabexate mesilate on the incidence of severe PEP.

**Fig. S53.** Forest plot demonstrating the effects of the 0.035-inch guidewire versus the 0.025-inch guidewire on the incidence of PEP.

**Fig. S54.** Forest plot demonstrating the effects of the endocut versus pure cut on the incidence of PEP.

**Fig. S55.** Forest plot demonstrating the effects of the endocut versus pure cut on the incidence of mild PEP.

**Fig. S56.** Forest plot demonstrating the effects of the endocut versus pure cut on the incidence of moderate PEP.

**Fig. S57.** Forest plot demonstrating the effects of the endocut versus pure cut on the incidence of severe PEP.

**Fig. S58.** Forest plot demonstrating the effects of early precut sphincterotomy on the incidence of PEP.

**Fig. S59.** Forest plot demonstrating the effects of pancreatic stent placement on the incidence of mild PEP in high-risk patients.

**Fig. S60.** Forest plot demonstrating the effects of pancreatic stent placement on the incidence of moderate PEP in high-risk patients.

**Fig. S61.** Forest plot demonstrating the effects of pancreatic stent placement on the incidence of severe PEP in high-risk patients.

**Fig. S62.** Forest plot demonstrating the effects of endoscopic sphincterotomy associated with balloon dilation versus endoscopic sphincterotomy on the incidence of PEP.

**Fig. S63.** Forest plot demonstrating the effects of pancreatic stent placement on the incidence of mild PEP.

**Fig. S64.** Forest plot demonstrating the effects of pancreatic stent placement on the incidence of severe PEP.

**Fig. S65.** Forest plot demonstrating the effects of endocut versus blend on the incidence of mild PEP.

**Fig. S66.** Forest plot demonstrating the effects of endocut versus blend on the incidence of moderate PEP.

**Fig. S67.** Forest plot demonstrating the effects of endocut versus blend on the incidence of PEP.

**Fig. S68.** Forest plot demonstrating the effects of the endoscopic papillary large balloon dilation versus combination of endoscopic papillary large balloon dilation and endoscopic sphincterotomy on the incidence of PEP.

**Fig. S69.** Forest plot demonstrating the effects of needle-knife precut versus transpancreatic sphincterotomy on the incidence of PEP in patients with difficult cannulation.

 **Fig. S70.** Forest plot demonstrating the effects of endoscopic sphincterotomy on the incidence of PEP.

**Fig. S71.** Forest plot demonstrating the effects of endoscopic papillary balloon dilation versus endoscopic sphincterotomy on the incidence of severe PEP.

**Fig. S72.** Forest plot demonstrating the effects of pancreatic stent placement on the incidence of mild to moderate PEP.

**Fig. S73.** Forest plot demonstrating the effects of pancreatic stent placement on the incidence of PEP in high-risk patients.

**Fig. S74.** Forest plot demonstrating the effects of needle knife fistulotomy on the incidence of PEP.

**Fig. S75.** Forest plot demonstrating the effects of early precut sphincterotomy on the incidence of PEP in patients with difficult biliary access

**Fig. S76.** Forest plot demonstrating the effects of endoscopic papillary balloon dilation versus endoscopic sphincterotomy on the incidence of PEP.

**Fig. S77.** Forest plot demonstrating the effects of guidewire-assisted cannulation on the incidence of PEP.

**Fig. S78.** Forest plot demonstrating the effects of guidewire-assisted cannulation on the incidence of mild PEP.

**Fig. S79.** Forest plot demonstrating the effects of guidewire-assisted cannulation on the incidence of moderate PEP.

**Fig. S80.** Forest plot demonstrating the effects of guidewire-assisted cannulation on the incidence of severe PEP.

**Fig. S81.** Forest plot demonstrating the effects of double-guidewire technique versus other methods on the incidence of mild PEP in patients with difficult biliary cannulation.

**Fig. S82.** Forest plot demonstrating the effects of double-guidewire technique versus other methods on the incidence of moderate PEP in patients with difficult biliary cannulation.

**Fig. S83.** Forest plot demonstrating the effects of double-guidewire technique versus other methods on the incidence of severe PEP in patients with difficult biliary cannulation.

**Fig. S84.** Forest plot demonstrating the effects of pure cut versus blend on the incidence of PEP.

**Fig. S85.** Forest plot demonstrating the effects of pure cut versus blend on the incidence of mild PEP.

**Fig. S86.** Forest plot demonstrating the effects of pure cut versus blend on the incidence of moderate PEP.

**Fig. S87.** Forest plot demonstrating the effects of pure cut versus blend on the incidence of severe PEP.

**Fig. S88.** Forest plot demonstrating the effects of double-guidewire technique versus other methods on the incidence of PEP in patients with difficult biliary cannulation.

**Fig. S89.** Forest plot demonstrating the effects of covered self-expandable metallic stents versus multiple plastic stents on the incidence of PEP.

**Fig. S90.** Forest plot demonstrating the effects of pancreatic stent placement on the incidence of PEP.

**Fig. S91.** Forest plot demonstrating the effects of pancreatic stent placement on the incidence of moderate PEP.
